# Over-optimism in unsupervised microbiome analysis: Insights from network learning and clustering

**DOI:** 10.1371/journal.pcbi.1010820

**Published:** 2023-01-06

**Authors:** Theresa Ullmann, Stefanie Peschel, Philipp Finger, Christian L. Müller, Anne-Laure Boulesteix

**Affiliations:** 1 Institute for Medical Information Processing, Biometry, and Epidemiology, Ludwig-Maximilians-Universität München, München, Germany; 2 Munich Center for Machine Learning (MCML), München, Germany; 3 Institute for Asthma and Allergy Prevention, Helmholtz Zentrum München, Neuherberg, Germany; 4 Department of Statistics, Ludwig-Maximilians-Universität München, München, Germany; 5 Institute of Computational Biology, Helmholtz Zentrum München, Neuherberg, Germany; 6 Center for Computational Mathematics, Flatiron Institute, New York, New York, United States of America; Fudan University, CHINA

## Abstract

In recent years, unsupervised analysis of microbiome data, such as microbial network analysis and clustering, has increased in popularity. Many new statistical and computational methods have been proposed for these tasks. This multiplicity of analysis strategies poses a challenge for researchers, who are often unsure which method(s) to use and might be tempted to try different methods on their dataset to look for the “best” ones. However, if only the best results are selectively reported, this may cause over-optimism: the “best” method is overly fitted to the specific dataset, and the results might be non-replicable on validation data. Such effects will ultimately hinder research progress. Yet so far, these topics have been given little attention in the context of unsupervised microbiome analysis. In our illustrative study, we aim to quantify over-optimism effects in this context. We model the approach of a hypothetical microbiome researcher who undertakes four unsupervised research tasks: clustering of bacterial genera, hub detection in microbial networks, differential microbial network analysis, and clustering of samples. While these tasks are unsupervised, the researcher might still have certain expectations as to what constitutes interesting results. We translate these expectations into concrete evaluation criteria that the hypothetical researcher might want to optimize. We then randomly split an exemplary dataset from the American Gut Project into discovery and validation sets multiple times. For each research task, multiple method combinations (e.g., methods for data normalization, network generation, and/or clustering) are tried on the discovery data, and the combination that yields the best result according to the evaluation criterion is chosen. While the hypothetical researcher might only report this result, we also apply the “best” method combination to the validation dataset. The results are then compared between discovery and validation data. In all four research tasks, there are notable over-optimism effects; the results on the validation data set are worse compared to the discovery data, averaged over multiple random splits into discovery/validation data. Our study thus highlights the importance of validation and replication in microbiome analysis to obtain reliable results and demonstrates that the issue of over-optimism goes beyond the context of statistical testing and fishing for significance.

## Author summary

Microbiome research focuses on communities of microbes, for example, those living in the human gut. To identify the structure of such communities, constructing microbial networks that represent associations between different microbes has become popular. The microbial associations are often further analyzed by applying cluster algorithms, i.e., researchers try to find groups (clusters) of microbes that are strongly associated with each other. Likewise, researchers are also interested in finding clusters of samples that are similar in bacterial compositions, often referred to as enterotypes. To produce broader and more reliable insights, networks and clustering results that have been constructed based on one specific dataset should generalize to other datasets as well. However, this may be compromised by the large number of statistical methods available for network learning and clustering. Due to uncertainty about which method to use, researchers might try multiple approaches on their dataset and pick the method which yields the “best” result (e.g., the network that has the highest number of strongly connected microbes). When many such methods are tried, the “best” method may be overly fitted to the specific dataset at hand, and the result may not generalize to new data. Our study demonstrates such over-optimism effects and gives recommendations for detecting and/or avoiding over-optimistic bias. We aim to generate greater awareness around this issue and to increase reliability of future microbiome studies.

This is a *PLOS Computational Biology* Methods paper.

## 1 Introduction

The popularity of microbiome research has surged in recent decades. Many hypotheses about the human microbiome, as well as the microbiome of other species or in various environments, are postulated and tested each year. At the same time, new statistical and computational methods for analyzing microbiome data are continually introduced. Microbiome analysis has yielded exciting results, leading to high hopes for new treatment and prevention options in medicine [1, 2].

In such a fast-moving and promising research field, validation is of vital importance to ensure the reliability of new results. Yet such practices may sometimes be neglected in favor of chasing new hypotheses. There is a certain danger of *over-optimism* in the field: New and exciting results might turn out to be non-replicable, i.e., they cannot be confirmed in studies with independent data. While a discussion about validation and replication has emerged in microbiome research in recent years [3, 4], it is not as advanced as in other fields such as psychology, where the so-called “replication crisis” has received considerable attention [5]. There is a lack of studies which illustrate the validation process in microbiome analysis and quantify over-optimism and (non)replicability. In particular, scant attention has been given to these topics in relation to *unsupervised* microbiome data analysis, e.g., network analysis and clustering.

In the present paper, we take a step toward filling this gap. We illustrate how over-optimism can arise in unsupervised microbiome analysis using four unsupervised “research tasks” as examples: clustering bacterial genera, finding hubs in microbial networks, differential network analysis, and clustering samples. The underlying idea is to model the approach of a “hypothetical researcher” who has these research tasks in mind and is confronted with a variety of methods to choose from. Due to uncertainty about the appropriate method to apply in the present case, the researcher might be tempted to try different analysis strategies and pick the “optimal result” for each task. We quantify the over-optimistic bias that can arise out of choosing the “best” method in this way, by validating the optimized results on validation data (which we will define shortly). Our primary interest does not lie in any of the four specific research tasks, but rather in demonstrating the importance of validation and the necessity of avoiding questionable research practices. Through this illustrative study, we aim to raise awareness for these topics in microbiome analysis.

We now explain our usage of the terms “over-optimism”, “validation”, and “replication”. Broadly speaking, over-optimism may result from two sources of *multiplicity*: a) multiplicity of (tested) hypotheses or b) multiplicity of analysis strategies. It is well known that *multiple testing* (i.e., testing multiple hypotheses on a dataset) can lead to false-positive results due to the accumulation of the type I-error probability. Such problems may appear in microbiome research, e.g., when testing many associations of microbiome-related variables with health-related variables and only reporting the significant results [3]. However, even when considering only a single hypothesis, the *multiplicity of analysis strategies* [6]—which we focus on in this paper—may lead to varied results and the potential for selectively reporting only the best ones. Researchers must make several choices about their analysis strategy (a mechanism known as “researcher degrees of freedom”, [7]), including data preprocessing (e.g., normalization) and statistical analysis in a narrower sense. Often, multiple analysis strategies are possible and sensible, which leads to *method uncertainty* [8] because it is not necessarily clear which analysis choice is the best one. In microbiome analysis, for example, a large number of methods for estimating and analysing microbial association networks exists [9], from which the researcher must choose.

In such situations, there is a temptation for the researcher to try different methods and then pick the one that yields the best result. This approach might be considered sensible: Finding the “best” method for the data appears to be a natural goal. However, when the number of tried methods is high, there is a substantial danger of “overfitting” the analysis to the present dataset. The best-performing method might thus perform well on the data currently used, but perhaps not as well on a validation dataset due to sampling variability—in other words, the optimized result cannot be (fully) validated or replicated on the validation data. Here, we define “replication” as applying the same methods of a study to new data [4]; see [10] for a more extensive discussion of the concept of replication. “Validation”, as we use it, is a broader term: A result is reappraised on a validation dataset, which may be either genuinely new data, or a dataset obtained by splitting the original data into two parts (discovery and validation data) [11]. We use the latter approach in our study.

The connection between the multiplicity of analysis strategies and over-optimism is occasionally mentioned in the literature, mostly in relation to significance testing [12]. For example, it is well known that trying different analysis choices can make it easier to find a statistically significant result [7, 13]. If the researcher does this in an intentional manner (i.e., tweaking the analysis choices sequentially until a “significant” *p*-value is reached), this is called *p-hacking* [14]. However, over-optimistic bias might also appear without conscious “hacking”: A researcher may try different methods with the best intentions but then proceed to *selective reporting* (reporting only the method that yields the best result). Additionally, such effects do not only pertain to significance testing, but may appear whenever the result of a statistical analysis is quantified (e.g., with a performance measure or an index value).

In this paper we focus on over-optimism in the context of unsupervised microbiome analysis, outside of the classical setting of significance testing. We illustrate the over-optimistic effects caused by the multiplicity of analysis strategies in combination with selective reporting, as quantified by the subsequent validation of the optimistic results. As exemplary data, we use OTU count data from the American Gut Project (AGP) obtained with 16S amplicon sequencing [15]. It is well known that technical variation in amplicon sequencing (e.g., batch effects with respect to different labs or different machines) or using different methods for clustering sequences to obtain OTUs may lead to variation in the generation of the OTU count data and the results of subsequent statistical analysis [4, 16–18]. In the present work, however, we focus on the multiplicity of the statistical analysis methods (starting from the processed OTU count table), which has received somewhat less attention than multiplicity stemming from different technical methods. Recently, some studies have highlighted that different statistical analysis methods or modeling strategies may yield inconsistent results, namely in the context of microbiome-disease association modeling [19], microbiome differential abundance methods [20], and analyzing microbiome intervention design studies [21]. In contrast to these studies, a) we focus on the multiplicity of *unsupervised* statistical methods, i.e., methods for network learning and clustering, and b) our main goal is not to compare the results of different methods, but rather to quantify over-optimism effects that stem from picking the “best” result. The range of the statistical methods we consider includes 1) normalization to make read counts comparable across samples and to account for compositionality (if required by the subsequent analysis steps), 2) estimation of microbial networks, sample networks, and (dis)similarity matrices, and 3) methods to further process the network/(dis)similarity information such as clustering.

**Fig 1:**
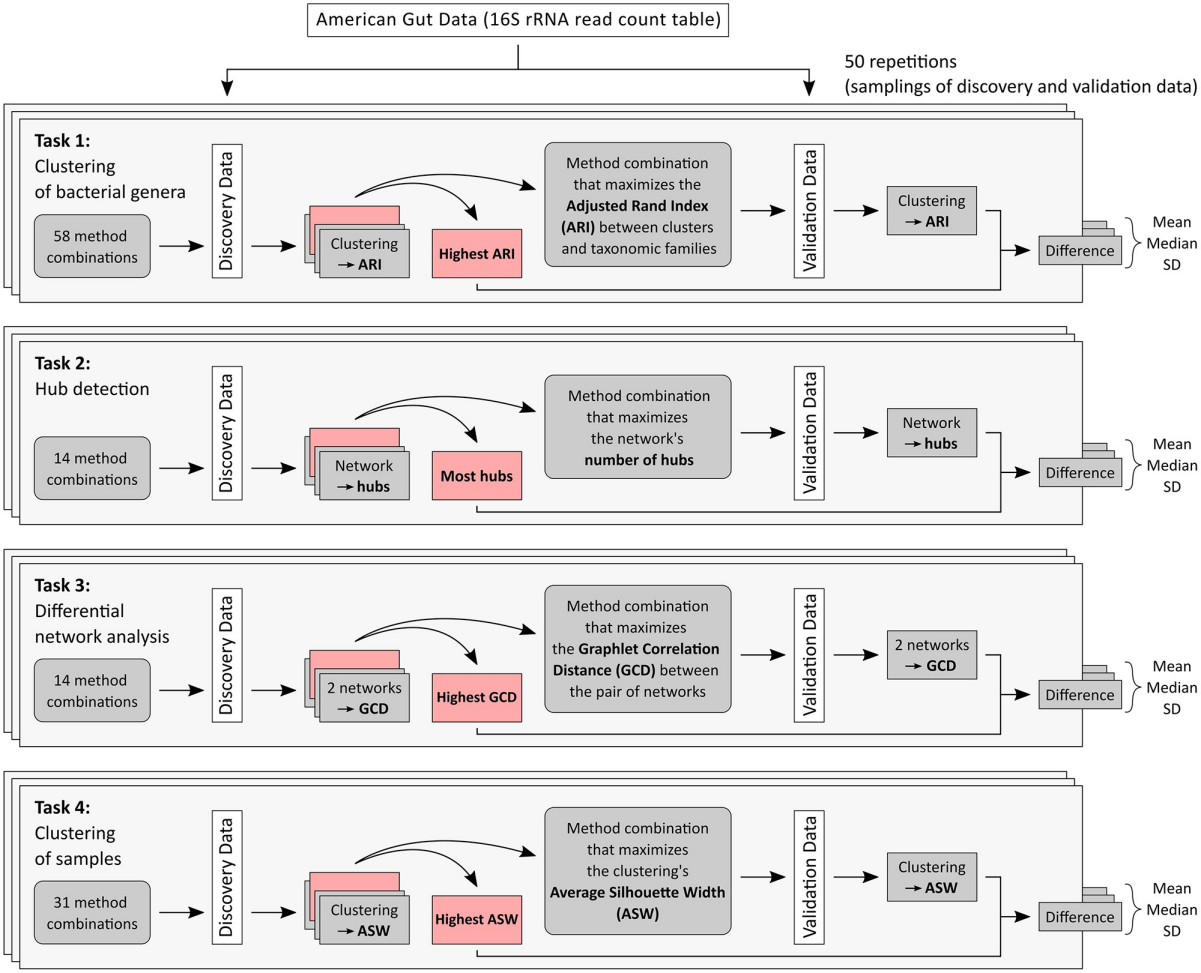
Graphical overview of our study. The process of drawing 50 samplings of discovery and validation data is repeated for different sample sizes: *n* ∈ {100, 250, 500, 1000, 4000} for tasks 1 and 2, *n* ∈ {100, 250, 500} for task 3, and *n* ∈ {100, 250, 500, 1000, 3500} for task 4.

The key idea of our illustrative study consists of splitting the whole dataset into a discovery and a validation set, trying out different methods for each of these three analysis steps on the discovery data, choosing the combination of methods that yields the best result on the discovery data according to an evaluation criterion, and applying this combination to the validation data to check whether the evaluation criterion takes a similar value. Fig 1 gives an overview of this approach, which we now describe in more detail.

We use four exemplary “research tasks” to illustrate the effects of the multiplicity of analysis strategies. Imagine a researcher who wishes to perform an unsupervised analysis of microbiome data. Even though the analysis is unsupervised and might be performed for exploratory purposes, the researcher usually has some hopes for the results. While these expectations could be vague at first, the researcher might eventually focus on a concrete evaluation criterion that represents these hopes in order to judge the results. The researcher tries different statistical methods and chooses the method that yields the best result according to the evaluation criterion. We now detail the four research tasks, the hopes that our hypothetical researcher might have, and the concrete evaluation criteria they might use (and which we therefore choose for our illustrative study):

1. **Clustering of bacterial genera:** Bacterial genera can be clustered based on their associations such that highly associated genera are likely to belong to the same cluster. Hence, the assignment of two genera to the same cluster indicates shared variation over the samples, which in turn might suggest a shared functionality. We assume that the hypothetical researchers hopes to find a clustering of bacterial genera that yields good agreement with the taxonomic categorization of the genera into families. As concrete evaluation criterion, we choose the Adjusted Rand Index (ARI, [22]), a measure for comparing two partitions, normalized for chance agreement. The ARI ranges in [−1, 1], with higher values indicating higher similarity of the partitions. In this research task, one partition is given by the clustering as calculated by the researcher, the other one by the taxonomic categorization of the genera into families. The higher the ARI (i.e., the closer to 1), the more similar the calculated clustering is to the taxonomic categorization, which indicates a “better” clustering. While it is typically not realistic to find a clustering that is *perfectly* aligned with the taxonomic categorization (i.e., where the ARI is equal to 1), some agreement with the taxonomy is often considered as a good property of a bacterial clustering [23]. While we perform the clustering at the genus level, the same logic would apply at any taxonomic level. This remark also holds for the other research tasks.
2. **Hub detection:** A researcher might hope to find a microbial network with interesting keystone taxa (also called “microbial hubs”), i.e., highly connected taxa which are assumed to have a strong impact on the rest of the network. Detecting and analyzing keystone taxa in order to better understand microbial interactions has become popular in recent years [24–26]. Taxa that are identified as hubs based on network centrality measures (see Section 4.3.2 for details) are not automatically *biologically* important keystone taxa [25]. Still, hub detection can serve as a starting point to carry out further analyses about the role of the detected hubs [27]. For example, a recent study [28] analyzed microbiome data from aquatic environments where many microbes are “unknown taxa”, i.e., uncharacterized. The authors generated microbial networks and performed hub detection. Frequently, the detected hubs were unknown taxa, which in turn serves to prioritize these specific taxa for further analyses.
  In our illustrative example, we assume that our hypothetical researcher is interested in generating as many interesting hypotheses and directions for further research as possible. Therefore, we assume that the researcher chooses a method that yields a relatively high number of hubs, to maximize the “hubbiness” of the network. Thus, the number of hubs is used as the concrete evaluation criterion. Of course, other criteria to choose an “interesting” network with hubs are also feasible.
3. **Differential network analysis:** Microbiome researchers are often interested in the effects of treatments, such as antibiotics, on the gut microbial community (see, e.g., [29, 30] for background). When generating microbial association networks for two groups (one for persons who did not take antibiotics in the last year, and one for persons who took antibiotics in the last month), a researcher might expect that the networks (as proxies for microbial community structure) potentially change. As concrete evaluation criterion we measure the dissimilarity between the networks with the Graphlet Correlation Distance (GCD) between the networks [31]. The method that yields the largest GCD between the two networks is chosen. The GCD has been used in previous studies to compare microbial networks [32–34].
4. **Clustering of samples:** The three previous research tasks are all based on associations between microbes. In contrast, the fourth task focuses on similarities between *samples* (individuals). The goal is to find a clustering of samples such that samples within the same cluster have a similar bacterial composition, while the composition differs between samples of different clusters. This task is inspired by the popular concept of “enterotypes”. In 2011, a study [35] argued that individuals can be clustered into three distinct groups which represent different gut microbiome types (enterotypes). Whether enterotypes truly exist (and if they do, how many there are) has since become a topic of controversial discussion [36–40]. Some studies have already noted that using different methods for clustering the samples (e.g., different methods for calculating the similarities between the samples) may lead to different enterotype results [37, 41]. However, to the best of our knowledge, the relation between the multiplicity of analysis strategies and over-optimism has not yet been explicitly studied. For this exemplary research task, we assume that the hypothetical researcher is interested in finding enterotypes in the AGP dataset. As concrete evaluation criterion, we use the Average Silhouette Width (ASW [42]). The ASW is a cluster validation index that measures the homogeneity as well as the separation of the clusters. The index ranges in [−1, 1], with higher values indicating a better clustering. The ASW has been previously used in enterotype studies to evaluate the quality of sample clusterings [35, 37, 41].

For each of the four research tasks, we imitate our “hypothetical researcher” by trying different methods (i.e., methods for estimating microbial networks, calculating similarities between samples, and/or clustering) and looking for the best result. The hypothetical researcher might stop at this point, and only report the best result according to the respective criterion. In contrast, we are interested in whether the best result can be confirmed on *validation data*: The result obtained by the “best” method on the discovery data (i.e., the “best” ARI, number of hubs, GCD, or ASW, respectively) is compared with the result obtained by this method on the validation data. The discovery and validation datasets are obtained by randomly sampling two disjoint subsets from the full AGP dataset, a process which is repeated multiple times.

Note that our analysis serves only illustrative purposes to study over-optimism effects. It is not our aim to systematically evaluate or compare the chosen method combinations. Moreover, we do not claim that researchers typically apply multiple methods to a dataset as systematically as we do this here, nor that they “optimize” for the best method with malicious intent. Nevertheless, during a longer research process, researchers will often try multiple methods on a dataset, and even if this happens with the best intentions, it might still cause over-optimism effects.

So far, we have spoken of imitating the behavior of a single hypothetical researcher or research team. Our study might also be interpreted as modeling the behavior of *multiple* research teams. Each team tries a different analysis strategy and only the team with the “best” result is able to publish their findings (e.g., due to publication bias).

We present the results of our analysis in Section 2. Section 3 contains a discussion. In Section 4, we give a detailed overview of the exemplary dataset, our study design, and the different statistical methods that we applied to the discovery data.

## 2 Results

### 2.1 Quantifying over-optimism effects

For each research task, we drew discovery and validation sets (each with sample size *n*) of varying sizes: *n* ∈ {100, 250, 500, 1000, 4000} for the first two research tasks, *n* ∈ {100, 250, 500} for the third research task, and *n* ∈ {100, 250, 500, 1000, 3500} for the fourth research task. For the third task, the maximal sample size was reduced due to the required information about antibiotics usage. For the fourth task, the maximal sample size was 3500 instead of 4000 because only samples from adults were kept for the analysis. More details are given in Section 4.2.

For each *n*, the process of drawing discovery and validation sets was repeated 50 times. As sampling variability decreases with increasing *n*, the performances of a method on both discovery and validation data should become more and more similar. We thus expected over-optimistic effects to decrease with increasing *n*.

For each research task, we applied multiple method combinations to the discovery data. For the first three research tasks which were based on microbial associations, this involved *normalization methods* (clr [43], mclr [44], and VST [45]), *association estimation* (Pearson correlation, Spearman correlation, latentcor [46], SPRING [44], and proportionality [47]), *sparsification* (*t*-test, threshold method, and neighborhood selection), and, for the first research task, *clustering* (hierarchical clustering, spectral clustering [48], fast greedy modularity optimization [49], the Louvain method for community detection [50], and manta [51]). For the fourth research task where samples were clustered based on their similarities, we applied *normalization methods* (clr, mclr, and VST), *similarity calculation* (Aitchison distance [52], Euclidean distance, compositional Kullback-Leibler divergence (cKLD) [53], and Bray-Curtis dissimilarity [54]), *sparsification* (threshold method, *K*-nearest neighbors), and *clustering* (Dirichlet multinomial mixtures (DMM) [55], spectral clustering, partitioning around medoids (PAM) [56], fast greedy modularity optimization, and the Louvain method for community detection). Detailed descriptions of the combinations are given in Section 4.3.

Supplementary figures in the Supporting Information S1, S2, S3 and S4 Text show the results of applying the different method combinations to the discovery data for the varying sample sizes (task 1: Fig A-J in S1 Text, task 2: Fig A-E in S2 Text, task 3: Fig A-C in S3 Text, task 4: Fig A-J in S4 Text). Notably, there is some change in the selected “best” method combination with respect to sample size. In particular, the performance of the sparsification methods is dependent on the sample size. These results are discussed in detail in S1, S2, S3 and S4 Text.

**Fig 2:**
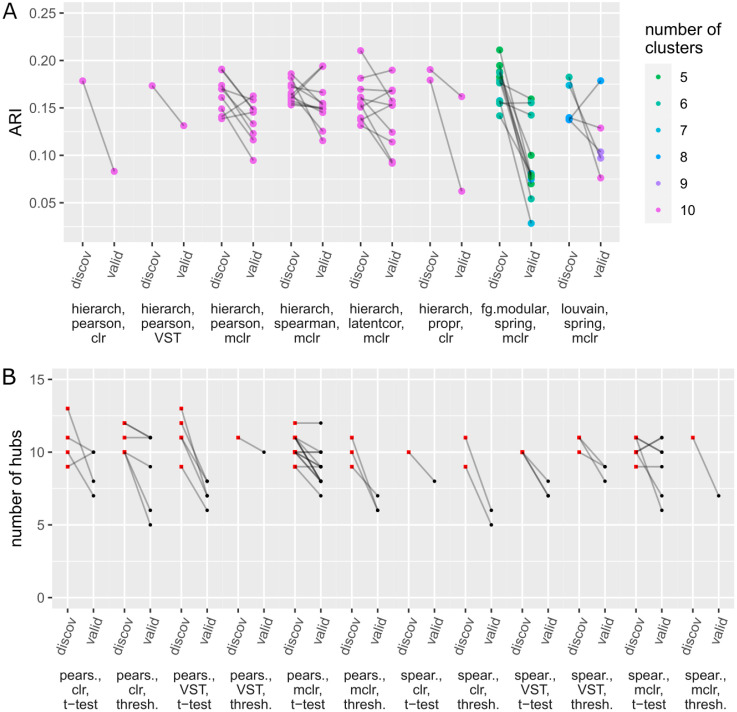
Research tasks 1 & 2: For *n* = 250, values of the evaluation criteria resulting from the “best” method combinations on the discovery data are compared to the corresponding results on the validation data. On the *x*-axis, the method combinations that performed best in at least one of the 50 samplings are shown. For each of the 50 samplings, the value of the evaluation criterion on the discovery data (belonging to the best method combination) and the corresponding value on the validation data are connected by a line, resulting in 50 lines overall. As the lines are slightly transparent, overlapping lines appear in a darker shade. a) ARI values for the task of clustering bacterial genera, b) numbers of hubs for the hub detection task.

**Fig 3:**
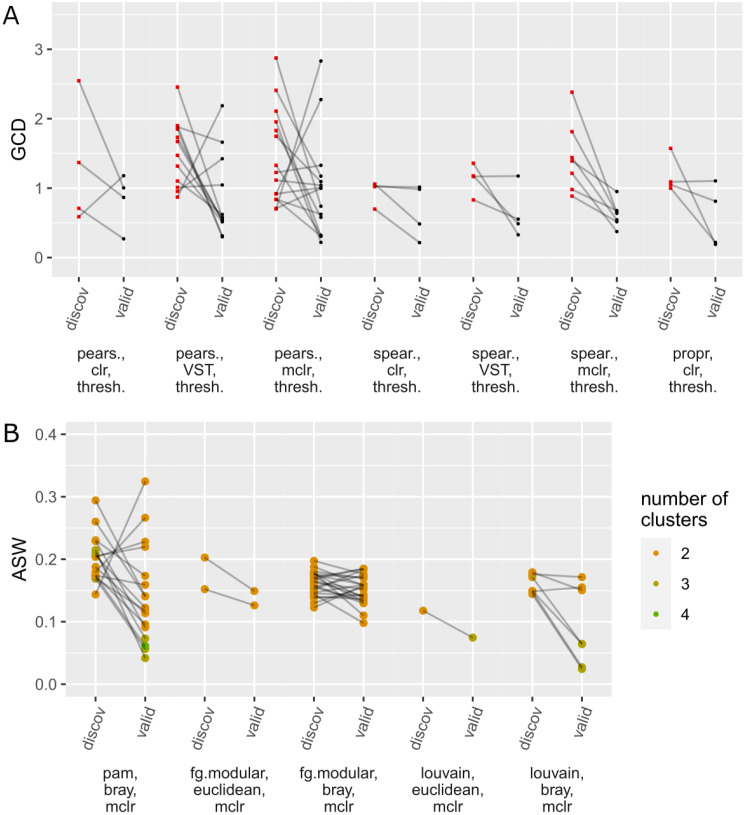
Research tasks 3 & 4: Analogously to Fig 2 (see the description there), values of the evaluation criteria are compared between discovery and validation data for *n* = 250. a) GCD values for the differential network analysis task, b) ASW values for the task of clustering samples.

Our main interest lies in choosing the method combination that yields the maximum value of the evaluation criterion (ARI, number of hubs, GCD, and ASW) on the discovery data, applying it to the validation data, and checking whether the values of the evaluation criteria can be validated. Over-optimism is indicated if the value of the evaluation criterion is lower on the validation data compared to the result on the discovery data. Exemplary results for *n* = 250 are shown in Fig 2 (research tasks 1 & 2) and Fig 3 (research tasks 3 & 4). The corresponding figures for all other sample sizes *n* are given in the Supporting Information (task 1: Fig K-O in S1 Text, task 2: Fig F-J in S2 Text, task 3: Fig D-F in S3 Text, task 4: Fig K-O in S4 Text).

On the *x*-axis, only the method combinations that performed best in at least one of the 50 samplings are shown (that is, *not all* tried method combinations; the method combinations that did not perform best in at least one of the samplings do not appear in the plot because these were never applied to the validation data). For each sampling, the value of the evaluation criterion on the discovery data (belonging to the best method combination) and the corresponding value on the validation data are connected by a line. For the first and fourth task, the dots representing the ARI/ASW values are colored according to the number *k* of clusters in the respective clustering result. Details about the procedures for determining *k* are given in Sections 4.3.1 and 4.3.4. For the other two research tasks, the results are shown as red squares for the discovery data and black dots for the validation data.

The lines point downwards in most cases, i.e., the results for the validation data are usually slightly worse than for the discovery data. This indicates over-optimism effects. To further quantify these effects, Table 1 (tasks 1 & 2) and Table 2 (tasks 3 & 4) show the mean, median, and standard deviation of the difference as well as the scaled difference between the value of the evaluation criterion on the validation data and the value on the discovery data (over the 50 samplings of discovery/validation data). While it might be interesting to test the differences between discovery and validation data for significance (to assess whether the results on the validation data are “significantly worse”), a suitable procedure for that purpose has not yet been proposed, to the best of our knowledge, and would need to be explored in further work. For cluster analysis, challenges related to this issue have been recently discussed [11]. Instead of calculating *p*-values, we report the “effect size” (mean divided by standard deviation) in Tables 1 and 2.

**Table 1:** For research tasks 1 and 2: Mean, median, and standard deviation (over 50 samplings of discovery/validation data) of the difference (both unscaled and scaled) between the value of the evaluation criterion on the validation data and the corresponding value on the discovery data. Additionally, the effect size (mean divided by standard deviation) is reported. *ARI*_*discov*_ denotes the best ARI on the discovery data and *ARI*_*valid*_ the ARI resulting from the corresponding method combination on the validation data. The quantities #*hubs*_*discov*_, #*hubs*_*valid*_ (number of hubs) are defined analogously.

**Research task 1: clustering of bacterial genera**								
	*ARI*_*valid*_ − *ARI*_*discov*_				$\frac{ARI_{valid}-ARI_{discov}}{ARI_{discov}}$			
*n*	mean	median	sd	mean/sd	mean	median	sd	mean/sd
100	-0.054	-0.046	0.044	-1.22	-30.0%	-26.7%	24.1%	-1.24
250	-0.039	-0.035	0.046	-0.84	-22.0%	-21.8%	26.3%	-0.84
500	-0.038	-0.037	0.038	-1.01	-21.4%	-20.6%	21.6%	-0.99
1000	-0.042	-0.035	0.037	-1.13	-23.7%	-20.1%	20.3%	-1.16
4000	-0.035	-0.033	0.035	-1.00	-19.0%	-18.3%	18.8%	-1.01
**Research task 2: hub detection**								
	#*hubs*_*valid*_ − #*hubs*_*discov*_				$\frac{\#hubs_{valid}-\#hubs_{discov}}{\#hubs_{discov}}$			
*n*	mean	median	sd	mean/sd	mean	median	sd	mean/sd
100	-2.44	-3	2.35	-1.04	-21.6%	-24.0%	21.3%	-1.02
250	-2.18	-2	1.78	-1.22	-20.5%	-20.0%	16.5%	-1.24
500	-2.12	-2	1.88	-1.13	-20.8%	-20.0%	17.9%	-1.16
1000	-1.64	-2	1.52	-1.08	-16.3%	-18.2%	15.4%	-1.06
4000	-1.12	-1	1.32	-0.85	-11.5%	-11.1%	13.8%	-0.83

**Table 2:** For research tasks 3 and 4: Mean, median, and standard deviation (over 50 samplings of discovery/validation data) of the difference (both unscaled and scaled) between the value of the evaluation criterion on the validation data and the corresponding value on the discovery data. Additionally, the effect size (mean divided by standard deviation) is reported. *GCD*_*discov*_ denotes the largest GCD on the discovery data and *GCD*_*valid*_ the GCD resulting from the corresponding method combination on the validation data. The quantities *ASW*_*discov*_, *ASW*_*valid*_ (average silhouette width) are defined analogously.

**Research task 3: differential network analysis**								
	*GCD*_*valid*_ − *GCD*_*discov*_				$\frac{GCD_{valid}-GCD_{discov}}{GCD_{discov}}$			
*n*	mean	median	sd	mean/sd	mean	median	sd	mean/sd
100	-0.481	-0.463	0.829	-0.58	-25.0%	-30.0%	55.5%	-0.45
250	-0.555	-0.516	0.856	-0.65	-26.7%	-52.8%	72.5%	-0.37
500	-0.305	-0.417	0.605	-0.50	-18.6%	-45.1%	63.5%	-0.29
**Research task 4: clustering of samples**								
	*ASW*_*valid*_ − *ASW*_*discov*_				$\frac{ASW_{valid}-ASW_{discov}}{ASW_{discov}}$			
*n*	mean	median	sd	mean/sd	mean	median	sd	mean/sd
100	-0.055	-0.043	0.088	-0.63	-20.0%	-22.6%	35.0%	-0.57
250	-0.036	-0.027	0.065	-0.55	-18.3%	-16.0%	36.8%	-0.50
500	-0.020	-0.017	0.041	-0.48	-10.6%	-10.1%	24.7%	-0.43
1000	-0.019	-0.002	0.039	-0.48	-11.2%	-1.6%	25.5%	-0.44
3500	-0.010	-0.010	0.017	-0.58	-7.2%	-8.0%	13.3%	-0.54

As expected, the means and medians of the differences are negative for all four research tasks and all sample sizes, demonstrating that the results on the discovery data were somewhat over-optimistic. The effect sizes (mean divided by standard deviation) are notable for all research tasks, albeit slightly smaller for the third and fourth research task. We now discuss the behavior of the average differences over the varying sample sizes *n* in more detail for each research task in turn.

*Research task 1 (clustering of bacterial genera)*: The average absolute decline of the ARI on the validation data is not drastic, but when considering the scaled difference, the ARI is reduced on the validation data by about 20–30% on average. Note that the absolute value of the mean/median ARI difference (both unscaled and scaled) is largest for *n* = 100, and smallest for *n* = 4000. This fits with our previously mentioned hypothesis that over-optimism effects are less pronounced when *n* is large. However, between 100 and 4000, there is no clear linearly decreasing tendency in the absolute mean/median ARI differences. Moreover, there is no clear tendency with respect to the effect sizes.

*Research task 2 (hub detection)*: The absolute values of the means and medians of the differences tend to decrease with increasing sample size. Again, this fits with our hypothesis that the over-optimistic bias decreases with increasing *n*. This tendency also largely holds for the effect sizes, although the absolute value of the effect size is slightly larger at *n* = 250 compared to *n* = 100, due to the larger standard deviation at *n* = 100.

*Research task 3 (differential network analysis)*: The absolute values of the means and medians do not monotonically decrease with increasing *n*: for *n* = 250, these are slightly larger than for *n* = 100. This is perhaps due to the fact that the sampling variability is still rather large at *n* = 250. At *n* = 500, however, the over-optimism effect appears to decrease, as evidenced by the drops in the absolute values of the average differences (both unscaled and scaled). For even higher sample sizes, we would expect to see a continuing decline of the over-optimistic bias, although we cannot confirm this due to the limited data availability.

*Research task 4 (clustering of samples)*: Similar to the first research task, the average absolute decline of the evaluation criterion (here, the ASW) on the validation data is not drastic. When considering the relative decline, the ASW values decrease on the validation data by about 20% on average for smaller sample sizes. Over-optimistic bias tends to be less pronounced for larger sample sizes. With respect to the median differences and effect sizes, the bias slightly increases again at the largest sample size of *n* = 3500, but the mean and median differences are quite small.

We not only analyzed the relation of over-optimistic bias with the sample size, but also expected over-optimistic bias to decrease if fewer method combinations were tried. To investigate this hypothesis, we repeated our analyses with a reduced number of method combinations: five instead of 58 for the first research task, three instead of 14 for the second and third research tasks, and five instead of 31 for the fourth research task. The chosen subsets of combinations as well as the results are described in detail in the Supporting Information S5 Text. The means and medians of the differences mostly remain negative for the different research tasks and sample sizes (indicating that some over-optimistic bias still exists), but as expected, the absolute values of the mean/median differences as well as the effect sizes tend to be smaller. This supports our hypothesis that over-optimistic bias is more pronounced the more method combinations are tried. Of course, the exact amount of over-optimistic bias still depends on the chosen (subset of) method combinations.

### 2.2 Additional stability analyses

While our main focus was to compare the “best” result on the discovery data to the corresponding result on the validation data (with respect to the evaluation criteria), we also report some additional stability results for the first two research tasks to further demonstrate that the methods do not necessarily yield stable results on discovery vs. validation data. For the task of clustering bacterial genera, we compared the clusterings on discovery vs. validation data with the ARI (while the agreement with the taxonomic categorization was ignored). This measure is denoted as *ARI*_*stab*_. The results are reported in Table 3. For the hub detection task, we compared the sets of hubs on discovery vs. validation data with the Jaccard index (on the genus level) and cosine similarity index (on the family level), as reported in Table 4. The indices are described in more detail in Section 4.3.

**Table 3:** Mean, median, and standard deviation of *ARI*_*stab*_, i.e., the ARI between the clusterings of bacterial genera on discovery and validation data, over 50 samplings of discovery/validation data.

	*ARI*_*stab*_		
*n*	mean	median	sd
100	0.361	0.329	0.111
250	0.509	0.491	0.166
500	0.604	0.574	0.168
1000	0.600	0.568	0.166
4000	0.763	0.792	0.140

**Table 4:** Mean, median, and standard deviation (over 50 samplings of discovery/validation data) of a) the Jaccard index which compares the set of hubs obtained on the discovery data with the set of hubs on the validation data, and b) the cosine similarity which compares these sets of hubs, but on the level of families.

	Jaccard			Cosine similarity		
*n*	mean	median	sd	mean	median	sd
100	0.236	0.250	0.109	0.881	0.911	0.112
250	0.359	0.357	0.119	0.922	0.955	0.078
500	0.443	0.429	0.116	0.948	0.969	0.060
1000	0.546	0.538	0.139	0.946	0.974	0.068
4000	0.709	0.727	0.147	0.975	0.984	0.026

For the clustering task, Table 3 shows that for smaller sample sizes, the mean ARIs are rather far away from 1, which indicates notable differences between the clusterings of the bacteria based on discovery vs. validation data. The clusterings tend to become more similar with increasing sample size, but even for *n* = 4000, the mean ARI of about 0.8 indicates that the clusterings are still different to some extent. This shows that the chosen clustering on the discovery data is not necessarily stable regarding cluster memberships when the result is validated on the validation data.

For the hub detection task, Table 4 demonstrates that the sets of hubs can be quite different between discovery and validation data, as measured with the Jaccard index (which ranges between 0 and 1). For smaller sample sizes, the similarity is particularly small. The Jaccard values increase with increasing sample size, but even at *n* = 4000, a mean value of about 0.7 shows that there are still notable dissimilarities between the sets of hubs. For the similarity on *family* level, we expected higher values (given that two hubs from the same family which differ on the genus level are counted as not equal for the Jaccard index and as equal for the cosine similarity). Indeed, the values of the cosine similarity (which ranges between -1 and 1), are generally quite high. Therefore, if one only interprets the hubs on family level (e.g., with respect to typical functions of the bacterial families), there is less danger of instability between discovery and validation data, compared to an interpretation on genus level.

We repeated the stability analyses with reduced numbers of tried methods combinations as described in the previous section. The results are reported in the Supporting Information S5 Text. Overall, the stability results are rather similar to the ones obtained with the full sets of method combinations.

## 3 Discussion

We have quantified over-optimism effects resulting from the multiplicity of analysis strategies coupled with selective reporting, using four exemplary microbiome research questions. Our results indicate an over-optimistic bias for all four research tasks. That is, when choosing the “best” method on the discovery data according to the maximization of an evaluation criterion, this criterion then tends to attain lower (“worse”) values on the validation data when the same method is applied. The exact size of the over-optimistic bias depends on the research task and sample size. Generally speaking, the over-optimistic bias tends to be more pronounced at smaller sample sizes, although the relation between sample size and optimistic bias is not always strictly monotonically decreasing in our analyses. Moreover, the over-optimistic bias also depends on the number of tried method combinations. When we tried fewer combinations, we still detected some over-optimistic bias, but the bias was less pronounced.

Additional stability analyses for the first two research tasks have illustrated that clustering solutions and sets of hubs—which have been yielded by a method on discovery data—do not necessarily remain stable when the same method is applied to validation data.

In summary, our study has demonstrated that the issue of over-optimism and instability of results goes beyond the context of statistical testing and fishing for significance, and pertains to unsupervised analysis strategies as well.

The number of tried method combinations in the analyses with all combinations (58 for the clustering of bacterial genera, 14 for hub detection and differential network analysis, 31 for the clustering of samples) may seem quite large for a single researcher to attempt. However, we would argue that these numbers are not that unrealistic. The method combinations are not independent of each other. Rather, the combinations are obtained by varying methods along the analysis pipeline (e.g., the type of sparsification). Modern software packages make it very easy to quickly switch from one method choice to another. Moreover, as mentioned in the introduction, our study might also be interpreted as modeling the behavior of *multiple* research teams. Large public datasets, such as the AGP data, are studied by many researchers. While a single researcher or research team might only try a few analysis strategies, the strategies tried by multiple teams could sum up to a much larger number.

In order to quantify over-optimism, we deliberately split a single dataset into two parts instead of using an independent dataset as validation data. With the latter approach, we could not have determined whether worse performance on the validation data indeed stemmed from the multiplicity of analysis strategies combined with selective reporting (which is the focus of our work), or was simply due to substantial differences between discovery and validation data (e.g., different populations). Of course, beyond the context of our study, using external data is generally important to check the validity and generalizability of results.

A constraint of our study is that for each research task in turn, we translated expectations of the “hypothetical researcher” into a single fixed evaluation criterion. Of course, researchers might have various expectations and thus multiple criteria in mind. On the one hand, it is likely more difficult for researchers to find a result that is simultaneously good with respect to *multiple* criteria, thus potentially reducing over-optimism effects. On the other hand, considering multiple criteria might allow researchers to pick one or a few criteria based on obtaining good results. This constitutes another source of multiplicity (adding to the sources of multiplicity considered in the present study), which in turn might increase over-optimistic bias. It would be interesting to analyze the effects of considering multiple criteria in future work.

Over-optimism can lead to unreliable results and might ultimately hinder research progress. We now discuss some strategies which may help researchers avoid over-optimistic bias in their application studies.

As illustrated by our analyses with a reduced number of method combinations, over-optimistic bias tends to decrease if fewer methods are tried. Therefore, the first option is to reduce the multiplicity of analysis strategies *before* the start of the analysis. Researchers should carefully consider which method is most suitable for their application. Here, guidance from *neutral comparison studies* can be relevant. Such studies compare existing methods (instead of introducing a novel method), and the authors of the study are neutral, i.e., they do not have a vested interest in a particular method showing better performance than the others and are as a group approximately equally familiar with all considered methods. We refer to [57, 58] for a more detailed discussion of this concept. It would be desirable if more neutral comparison studies were published in the context of methodological research on microbiome analysis. For example, two recent studies already provide such a welcome effort in the context of microbial differential abundance testing [20, 59], and guidelines for benchmarking microbiome analysis methods have been proposed as well [60].

An additional strategy is *preregistration* of the researchers’ analysis plan. Preregistering refers to defining the research hypotheses and analysis plan, and posting this plan to a registry, *before* observing the results. This concept has gained plenty of attention in recent years [61]. Once their analysis plan is registered, researchers might shy away from trying many other analysis strategies and selectively reporting only the best results.

However, preregistration might not always be possible or sensible: for example, in exploratory research, researchers typically cannot pin down the exact analysis strategy in advance, and trying out different methods sequentially is quite natural [4]. Indeed, unsupervised analysis methods, on which we have focused in our study, are often used for exploratory purposes. In such cases, when the multiplicity of analysis strategies cannot be avoided, researchers should honestly report that their study is exploratory and that multiple methods were tried. They should not present their analyses as if a single analysis pipeline was fixed in advance, nor should they report only the “best” results.

In general, we would advise researchers to use validation data to validate their results whenever possible. While we have included validation data in our study to quantify over-optimism effects, researchers can also use validation data in their applied research, to check whether the best results on the discovery data still hold on the validation data. This is particularly relevant when the multiplicity of possible analysis strategies cannot be reduced beforehand, e.g., in the absence of relevant neutral comparison studies for the methods of interest. For the topic of cluster analysis (research tasks 1 and 4), different strategies for validating clustering results on validation data have been previously discussed in detail [11]. More awareness for the importance of validation data has also emerged in microbiome research (see, e.g., in the context of supervised analysis [62, 63] and large-scale cohort studies [64]). Using validation data does not directly *prevent* over-optimism on the discovery data, but helps to *detect* over-optimistic results. The evaluation on the validation data can be considered as a more realistic assessment of the quality of the result, thus correcting for over-optimistic bias.

Sometimes, validation data is not available, e.g., because the dataset is too small to be split into discovery and validation sets, and a suitable independent validation set does not exist. For such cases, it would be interesting to find other indicators of potential over-optimism. Researchers might check, for instance, whether the results from the different tested methods coincide. Similarity of the results indicates robustness with respect to method choice. However, lack of robustness does not automatically imply that the results (or the “best” result) will also be over-optimistic, in the sense that they cannot be validated on validation data. Vice versa, if the results are robust, it is not entirely clear to which extent this is an indicator of nonexistent or small over-optimistic bias (although a reduced extent of over-optimism might be somewhat likely because obtaining very similar results would not allow researchers to pick a single result that is notably better than the other ones). It might be interesting to study the relation between robustness and replicability on validation data in further work.

The present study does not aim at systematically evaluating the performance of any chosen method combination. In particular, we do not give recommendations about which methods to use. In future research, it might be interesting to explore whether the design used in this study could be adapted to method evaluation and comparison. More precisely, one might repeatedly sample discovery and validation datasets as in our study, and evaluate methods based on whether they a) have a good performance on the discovery data and b) have a similar performance on the validation data, i.e., do not tend to overfit to the discovery data.

In summary, we hope that our study helps raise awareness of the important problem of over-optimism in microbiome research, and that it motivates more widespread implementation of strategies to avoid over-optimistic bias. If researchers adhere to good research practices, the results of microbiome analyses will likely become more reliable and replicable in the future.

## 4 Materials and methods

### 4.1 Dataset

We used data from the American Gut Project [15], a large citizen-science initiative. The project collected (mainly) fecal samples from participants in the United States, United Kingdom, and Australia. The researchers also collected metadata on the participants, e.g., health status, disease history, and lifestyle variables. Bacterial abundances were obtained using high-throughput amplicon sequencing, targeting the V4 region of the 16S rRNA marker gene with subsequent variant calling.

We downloaded an OTU count table for unrarefied bacterial fecal samples (dating from 2017) from the project website http://ftp.microbio.me/AmericanGut/ag-2017-12-04/, together with metadata about the samples. The OTU count table originally contained *p* = 35511 OTUs and *N* = 15148 samples. Following [23], we performed three preprocessing steps: 1) removing samples with a sequencing depth of less than 10000 counts, 2) removing OTUs which were present in less than 30% of the remaining samples, 3) removing 10% of the remaining samples, namely the samples with a sequencing depth under the 10%-percentile. The resulting OTU count table comprises *p* = 531 OTUs and *N* = 9631 samples.

For all four research tasks, the analysis was performed on the taxonomic rank of genera, to which the data were agglomerated. OTUs with unknown genus were assigned their own individual genus, which resulted in *p* = 323 genera overall.

### 4.2 Sampling of discovery and validation datasets

We obtained discovery and validation datasets by randomly sampling two disjoint subsets from the full AGP dataset. For each research task, the process of sampling discovery and validation data was performed along the *samples* of the AGP data (i.e., the subjects), not along the bacteria. This is because in each task, the bacteria formed a fixed set of entities of specific interest. This set thus remained constant for both discovery and validation data. For clustering, this is discussed in more detail in [11].

Discovery and validation sets (each with sample size *n*) were drawn of varying sizes: *n* ∈ {100, 250, 500, 1000, 4000} for the first two research tasks (clustering of bacterial genera and hub detection), *n* ∈ {100, 250, 500} for the third research task (differential network analysis), and *n* ∈ {100, 250, 500, 1000, 3500} for the fourth research task (clustering of samples). For differential network analysis, the maximal sample size was reduced because we only considered samples that did not take antibiotics in the last year as well as samples that took antibiotics in the last month. There were 6901 samples that fulfilled these criteria. Moreover, the sampling was stratified according to antibiotics use; for discovery and validation data each, we drew *n*/2 samples that did not take antibiotics in the last year and *n*/2 samples that took antibiotics in the last month. Because there are only 544 persons who took antibiotics in the last month, the maximum *n* is reduced to 500. For sample clustering, the maximum *n* is 3500 instead of 4000 because we only kept samples from adults between ages 20–65 (7145 samples overall). We focused on this age group because previous studies have shown that the composition of the gut microbiome varies across age [65–67], with potentially more extreme “enterotypes” in children and the elderly [40, 68].

### 4.3 Methods for unsupervised microbiome analysis

In this section, we discuss which method combinations were applied to the discovery data, and how the results were evaluated on the validation data.

#### 4.3.1 Research task 1: Clustering bacterial genera

We varied different steps of the cluster analysis process, resulting in 58 method combinations that were tried on the discovery data. In this section we explain how the 58 combinations were obtained.

We used cluster algorithms from two categories. Algorithms from the first category are based on (dis)similarity matrices: hierarchical clustering and spectral clustering [48]. Algorithms from the second category are based on networks with weighted edges: fast greedy modularity optimization [49], the Louvain method for community detection [50], and the manta algorithm [51].

To generate either (dis)similarity matrices or weighted networks, associations (*r*_*ij*_)_*i*,*j*_ between the microbes must be calculated. Beforehand, often zero handling and normalization of the data are required. Table 5 gives an overview of the method combinations used for calculating the associations *r*_*ij*_ for later use in (dis)similarity based clustering, i.e., for generating (dis)similarity matrices which will later be used as input for hierarchical and spectral clustering. We used four different association measures. The first ones are the Pearson and Spearman correlations, which require normalization to account for compositionality. Here we used either the centered log-ratio transformation (clr, [43]), the modified clr transformation (mclr, [44]), or the variance-stabilizing transformation (VST, [45]). As the clr and VST methods cannot handle zeros in the count data, a pseudo count of 1 was added to the count data before normalizing with these methods (mclr, on the other hand, can deal with zeros). Apart from the Pearson and Spearman correlations, we used the semi-parametric rank-based correlation, which is based on estimating the latent correlation matrix of a truncated Gaussian copula model (latentcor, [46, 69]). Since the latentcor method requires normalized counts that are strictly non-negative, it was only combined with the mclr transformation. The final association measure is proportionality [47, 70]. Proportionality is a compositionally aware method that measures associations between log-ratio transformed variables [47]. We thus used the clr transformation as proposed in [47] and replaced zero counts by a pseudo count.

**Table 5:** Method combinations for generating microbial associations, which are then transformed into (dis)similarity matrices. The (dis)similarity matrices were used as input for hierarchical and spectral clustering.

Zero handling	Normalization	Association estimation
pseudo	clr	Pearson
pseudo	VST	Pearson
none	mclr	Pearson
pseudo	clr	Spearman
pseudo	VST	Spearman
none	mclr	Spearman
none	mclr	latentcor
pseudo	clr	proportionality

Table 6 shows the method combinations used for calculating the associations *r*_*ij*_ for later use in network-based clustering. That is, these methods were used for generating weighted networks. The method combinations are very similar to the methods in Table 5 for generating (dis)similarity matrices. Indeed, weighted networks are also based on (dis)similarity matrices, but the generation contains an additional sparsification step, as explained below. Again, the Pearson and Spearman correlations were used with the respective normalization and/or zero handling methods. We also used the SPRING method [44], which combines the latentcor correlation estimation with sparse graphical modeling techniques, namely by using the neighborhood selection technique [71] for sparse estimation of partial correlations. Finally, we used the proportionality measure.

**Table 6:** Method combinations for generating weighted microbial association networks. The networks were used as input for fast greedy modularity optimization, Louvain community detection, and manta.

Zero handling	Normalization	Association estimation	Sparsification
pseudo	clr	Pearson	*t*-test
pseudo	clr	Pearson	threshold
pseudo	VST	Pearson	*t*-test
pseudo	VST	Pearson	threshold
none	mclr	Pearson	*t*-test
none	mclr	Pearson	threshold
pseudo	clr	Spearman	*t*-test
pseudo	clr	Spearman	threshold
pseudo	VST	Spearman	*t*-test
pseudo	VST	Spearman	threshold
none	mclr	Spearman	*t*-test
none	mclr	Spearman	threshold
none	mclr	SPRING	neighborhood selection
pseudo	clr	proportionality	threshold

To generate a weighted network, the associations *r*_*ij*_ (which are usually different from zero) were not directly used as an adjacency matrix—otherwise, the network would be dense. Therefore, the associations *r*_*ij*_ were transformed into sparsified values $r_{ij}^{*}$ by setting some $r_{ij}^{*}$ to zero to indicate that *i* and *j* are not connected, $r_{ij}^{*}=r_{ij}$ otherwise. For sparsification of the Pearson and Spearman correlations *r*_*ij*_, we used either Student’s *t*-test or the threshold method. The former sets $r_{ij}^{*}=0$ if the association *r*_*ij*_ is not significantly different from 0 according to the *t*-test. The *p*-values were adjusted for multiple testing via the local false discovery rate [72]. For the threshold method, we set $r_{ij}^{*}=0$ if *r*_*ij*_ < *c* for some fixed threshold value *c* (we use *c* = 0.15 which gave reasonable results in preliminary analyses, not shown). For the proportionality measure, we used threshold sparsification. SPRING already comes with inbuilt sparsification given by the neighborhood selection method.

After calculating the associations as in Tables 5 and 6, they were then transformed as follows (the pipeline and notations are taken from [9]):

- For (dis)similarity based clustering (Table 5): A dissimilarity matrix *D* = (*d*_*ij*_) for hierarchical clustering is calculated via $d_{ij}=\sqrt{0.5(1-r_{ij})}$. A similarity matrix *S* = (*s*_*ij*_) for spectral clustering is obtained by setting *s*_*ij*_ = 1 − *d*_*ij*_.
- For network-based clustering (Table 6): A weighted network is constructed as follows. For the edges *ij* with $r_{ij}^{*}\neq 0$ (i.e., the edges that remain after sparsification), the distances *d*_*ij*_ and similarities *s*_*ij*_ are calculated as in a). Finally, the weighted network is represented as an adjacency matrix *A* = (*a*_*ij*_) with *a*_*ij*_ = *s*_*ij*_ for *ij* with $r_{ij}^{*}\neq 0$, and *a*_*ij*_ = 0 otherwise.

The (dis)similarity matrices and networks were then used as input for clustering. For hierarchical and spectral clustering, we fixed the number of clusters at *k* = 10, which was inspired by the ten different taxonomic classes in the data. Also, *k* = 10 tends to yield better ARI results than *k*s lower than ten (preliminary analysis, not shown). *k*s higher than ten were not tried because we aimed to emulate a researcher who wants to find an interpretable, handy clustering (there are 34 different taxonomic families, but 34 clusters are not easily interpretable). The other clustering algorithms all have inbuilt mechanisms for determining *k*. Forcing *k* to be 10 for these methods generally did not improve the results (not shown). However, *k* can be indirectly influenced via the sparsification: The sparser the network, the more clusters tend to be found. This is one of the reasons we set the threshold for threshold sparsification at *c* = 0.15 because this value generally yielded sufficiently high *k*s to find good results, but only rarely *k*s that are so high that the clusters are difficult to interpret.

Overall, the method combinations yielded 58 different clustering results on the discovery data: 16 based on (dis)similarity clustering (eight rows in Table 5 times two cluster algorithms), and 42 based on network clustering (fourteen rows in Table 6 times three cluster algorithms). The best one out of the 58 clustering results was chosen, i.e., the clustering with the highest ARI regarding the taxonomic categorization into families. The corresponding method combination was applied to the validation data. The ARI between the clustering on the validation data and the taxonomic categorization was computed and compared with the best ARI on the discovery data. If the ARI on the validation data was lower, this was an indication that the best ARI on the discovery data was over-optimistic.

As an additional stability analysis, we compared the chosen clustering on the discovery data with the clustering on the validation data, again using the ARI.

#### 4.3.2 Research task 2: Hub detection

Here, we wanted to generate sparse weighted microbial association networks. For this purpose, we used the same methods as in Table 6. Thus, 14 method combinations were tried on the discovery data.

For hub detection in the resulting networks, hubs were defined as nodes that have the highest degree, betweenness, and closeness centrality [25]. More precisely, we determined the hubs as the nodes with centrality values above the 95% empirical quantile, for each of the three centrality measures simultaneously. The centralities are defined as follows [73]: The degree centrality denotes the number of adjacent nodes. The betweenness centrality measures the fraction of times a node lies on the shortest path between all other nodes. The closeness centrality of a node is the reciprocal of the sum of shortest paths between this node and all other nodes. All centrality measures were normalized to be comparable between networks of different sizes (see [9] for details). The centralities were only calculated for the largest connected component of each network (i.e., the largest subgraph of the network in which all nodes are connected); centrality values of nodes in the disconnected component were set to zero. We assumed that “hubs” in small parts of the network that are disconnected from the majority of the nodes are of less interest to researchers. Moreover, the betweenness and closeness centrality depend on shortest paths, which are not well-defined for nodes in different unconnected sub-graphs.

After applying the 14 method combinations and calculating the hubs for each resulting network, the method combination that yielded the highest number of hubs was chosen. If there were multiple method combinations that attained the maximal number of hubs, we chose the combination that yielded higher mean centrality values of the hubs. More specifically, for each set of hubs that corresponds to a method combination, the mean values of the three centrality measures were calculated over the hubs. Then for each centrality measure separately, the sets of hubs were ranked according to these mean values. Finally, the set of hubs (and thus the corresponding method combination) that yielded the highest mean rank over all three centrality measures was chosen.

The “best” method combination was then applied to the validation data. The number of hubs in the microbial network on the validation data was calculated and compared with the highest number of hubs on the discovery data. Over-optimism was indicated if the number of hubs was lower on the validation data.

Additionally, we reported the similarity of the sets of hubs determined on the discovery vs. validation data with the Jaccard index [74]: let *H*_*discov*_, *H*_*valid*_ be the sets of hubs for the discovery resp. validation data, then

$$\text{Jacc}\left(H_{discov},H_{valid}\right)=\frac{|H_{discov}\cap H_{valid}|}{|H_{discov}\cup H_{valid}|}.$$

The Jaccard index takes values in [0, 1], and is closer to 1 the more similar the sets are. The similarity between the sets of hubs was also assessed on the higher taxonomic level of families with the cosine similarity index. More precisely, assume that the hubs (genera) in the union *H*_*discov*_∪*H*_*valid*_ belong to *l* distinct families overall. Let $f^{\left(d\right)}=(f_{1}^{\left(d\right)},\ldots ,f_{l}^{\left(d\right)})$ be the family frequency vector for *H*_*discov*_, that is, each entry $f_{j}^{\left(d\right)}$ counts how many hubs in *H*_*discov*_ belong to family *j*. Analogously, let **f**^(*v*)^ be the family frequency vector for *H*_*valid*_. The vectors **f**^(*d*)^ and **f**^(*v*)^ are then compared with the cosine similarity index:

$$\text{cos}\;\text{sim}\left(f^{\left(d\right)},f^{\left(v\right)}\right)=\frac{\sum_{j=1}^{l}f_{j}^{\left(d\right)}f_{j}^{\left(v\right)}}{\sqrt{\sum_{j=1}^{l}{\left(f_{j}^{\left(d\right)}\right)}^{2}}\sqrt{\sum_{j=1}^{l}{\left(f_{j}^{\left(v\right)}\right)}^{2}}}$$

The cosine similarity index ranges in [0, 1], with higher values indicating higher similarity.

#### 4.3.3 Research task 3: Differential network analysis

As described in Section 4.2, the discovery and validation datasets each consisted of two halves: persons who did not take antibiotics in the last year (“non-antibiotics samples”), and persons who took antibiotics in the last month (“antibiotics samples”). The methods for generating weighted microbial association networks as in Table 6 were applied separately to the antibiotics and non-antibiotics samples of the discovery data.

The resulting networks were compared with the Graphlet Correlation Distance (GCD, [31]). This distance measures the similarity of the networks based on small induced subgraphs, so-called graphlets. All graphlets composed of up to four nodes are considered, and the automorphism orbits of these graphlets are enumerated (orbits represent the “roles” that nodes can play in the graphlets). For each node in a given network, one can count how often the node participates in each graphlet at the respective orbits. Only 11 non-redundant orbits are considered here. Based on these orbit counts across all nodes, the 11 × 11 Spearman correlation matrix among the 11 orbits is calculated, which represents a robust and size independent network summary statistics. For comparing two networks, the Spearman correlation matrix is calculated for each network in turn. Then the Euclidean distance between the upper triangular parts of these matrices is calculated, resulting in the GCD.

In our study, the network generation method that yielded the largest GCD between the antibiotics network and the non-antibiotics network was chosen as the “best” one and applied to the antibiotics and non-antibiotics samples in the validation data. Again, the resulting networks were compared with the GCD. If the GCD on the validation data was smaller (i.e., the antibiotics vs. non-antibiotics networks were more similar than on the discovery data), this indicated over-optimism.

#### 4.3.4 Research task 4: Clustering of samples

Similar to the first research task, both (dis)similarity-based and network-based cluster algorithms were applied to the discovery data (resulting in 31 clusterings overall). In contrast to the first task, dissimilarities between samples instead of microbes were calculated, and sample networks instead of microbial association networks were estimated (i.e., networks in which nodes correspond to samples, not taxa).

We considered partitioning around medoids (PAM) [56] as well as spectral clustering as instances of (dis)similarity-based clustering algorithms. We chose PAM since it has been frequently used in enterotype studies [35, 37, 41, 68]. For this research task, we excluded hierarchical clustering because this algorithm frequently resulted in clusters with nearly all samples contained in one cluster and only a few samples in other clusters (this phenomenon did not occur to the same extent in the clustering of bacterial genera). Presumably, researchers would be less interested in such clustering results.

From the category of network-based cluster algorithms, we chose fast greedy modularity optimization and the Louvain method for community detection. The manta algorithm was not chosen because it was explicitly developed for clustering taxa, not samples.

We also included clustering based on Dirichlet multinomial mixtures (DMM) [55]. In contrast to the cluster algorithms listed above, DMM does not require calculation of dissimilarities between samples and can be applied directly to the microbial count matrix. The DMM method has been used in several studies to detect enterotypes [55, 68, 75].

Table 7 presents the different methods for calculating dissimilarities (*d*_*ij*_)_*i*,*j*_ between the samples, which are then used as input for PAM and spectral clustering. We used the Aitchison distance [52] which is defined as the Euclidean distance between clr-transformed compositions. We also combined the Euclidean distance with the VST and mclr normalization. Moreover, we applied the compositional Kullback-Leibler divergence (cKLD) [53]. The cKLD measure is suitable for application on compositional data; thus, the counts are merely transformed into fractions (relative abundances) before the measure is applied. Finally, we applied the Bray-Curtis dissimilarity measure [54], which requires non-negative values as input and is therefore combined with the mclr normalization.

**Table 7:** Method combinations for dissimilarity calculation. The dissimilarity matrices were used as input for PAM and spectral clustering.

Zero handling	Normalization	Association estimation
pseudo	clr	Aitchison
pseudo	VST	Euclidean
none	mclr	Euclidean
pseudo	fractions	cKLD
none	mclr	Bray-Curtis

The dissimilarities *d*_*ij*_ were scaled to [0, 1], resulting in values $d_{ij}^{scale}$ (see [9] for details). The scaled dissimilarities were used as input for PAM. Similarities *s*_*ij*_ for spectral clustering were obtained by setting $s_{ij}=1-d_{ij}^{scale}$.

For network-based clustering, the same methods for calculating dissimilarities as in Table 7 were used, but with an additional sparsification step. This is displayed in Table 8. The scaled dissimilarities $d_{ij}^{scale}$ were transformed into sparsified values $d_{ij}^{*}$, either with the threshold method (by setting $d_{ij}^{*}$ to 1, i.e., the maximum dissimilarity, if $d_{ij}^{scale}>0.85$), or with the *K*-nearest neighbor method (each node is connected to the *K* = 3 nodes with minimum dissimilarity; if nodes *i* and *j* are not connected after this procedure, $d_{ij}^{*}$ is set to 1). The weighted sample network is then represented as an adjacency matrix *A* = (*a*_*ij*_) with $a_{ij}=s_{ij}=1-d_{ij}^{*}$, with *a*_*ij*_ = 0 for sparsified edges.

**Table 8:** Method combinations for generating weighted sample networks. The networks were used as input for fast greedy modularity optimization and Louvain community detection.

Zero handling	Normalization	Association estimation	Sparsification
pseudo	clr	Aitchison	threshold
pseudo	clr	Aitchison	*K*-NN
pseudo	VST	Euclidean	threshold
pseudo	VST	Euclidean	*K*-NN
none	mclr	Euclidean	threshold
none	mclr	Euclidean	*K*-NN
pseudo	fractions	cKLD	threshold
pseudo	fractions	cKLD	*K*-NN
none	mclr	Bray-Curtis	threshold
none	mclr	Bray-Curtis	*K*-NN

DMM clustering, fast greedy modularity optimization, and Louvain commmunity detection all have inbuilt mechanisms for determining the number of clusters *k*. For PAM and spectral clustering, we tried different values *k* ∈ {2, 3, …, 10} and chose the *k* that maximized the ASW of the clustering.

For calculating the ASW of a clustering, a corresponding dissimilarity matrix is required. For most clustering results, we used the dissimilarity matrix that was calculated one step before applying the cluster algorithm. The only exception are clustering results obtained by DMM which does not require prior calculation of dissimilarities. We calculated the ASW values for DMM clustering results based on the Bray-Curtis dissimilarity matrix since the authors of the DMM method used this dissimilarity measure to visualize their clustering results [55].

Overall, the considered method combinations led to 31 different clustering results on the discovery data: one based on DMM clustering, ten based on (dis)similarity clustering (five rows in Table 7 times two cluster algorithms), and 20 based on network clustering (ten rows in Table 8 times two cluster algorithms). The method combination that yielded the clustering with the highest ASW value was chosen and applied to the validation data, with over-optimistic bias indicated by lower ASW values on the validation data.

### 4.4 Technical implementation

All analyses were performed with R, version 4.0.4 and Python, version 3.6.13. Our fully reproducible code is available at https://github.com/thullmann/overoptimism-microbiome. (Dis)similarity matrices and weighted networks were generated with the R package NetCoMi [9]. Spectral clustering was performed with a previously published R implementation [23]. For fast greedy modularity optimization and the Louvain method for community detection, we used the R package igraph [76]. For clustering with manta, we accessed the Python implementation [51] with the reticulate interface for R [77]. We used the R package cluster [78] for PAM clustering, and the R package DirichletMultinomial [79] for DMM clustering. Orbit counts for the calculation of the GCD were generated with the R package orca [80].

## Supporting information

S1 Text Full results and plots for research task 1 (clustering of bacterial genera). (PDF)

S2 Text Full results and plots for research task 2 (hub detection). (PDF)

S3 Text Full results and plots for research task 3 (differential network analysis). (PDF)

S4 Text Full results and plots for research task 4 (clustering of samples). (PDF)

S5 Text Analyses with a reduced number of method combinations. (PDF)

## References

[1] Zmora N, Soffer E, Elinav E. Transforming medicine with the microbiome. Science Translational Medicine. 2019;11(477):eaaw1815. doi: 10.1126/scitranslmed.aaw1815 30700573

[2] Kuntz TM, Gilbert JA. Introducing the microbiome into precision medicine. Trends in Pharmacological Sciences. 2017;38(1):81–91. doi: 10.1016/j.tips.2016.10.001 27814885

[3] Kim D, Hofstaedter CE, Zhao C, Mattei L, Tanes C, Clarke E, et al. Optimizing methods and dodging pitfalls in microbiome research. Microbiome. 2017;5(1):52. doi: 10.1186/s40168-017-0267-5 28476139 PMC5420141

[4] Schloss PD. Identifying and overcoming threats to reproducibility, replicability, robustness, and generalizability in microbiome research. mBio. 2018;9(3):e00525–18. doi: 10.1128/mBio.00525-18 29871915 PMC5989067

[5] Open Science Collaboration. Estimating the reproducibility of psychological science. Science. 2015;349(6251):aac4716. doi: 10.1126/science.aac4716 26315443

[6] Hoffmann S, Schönbrodt F, Elsas R, Wilson R, Strasser U, Boulesteix AL. The multiplicity of analysis strategies jeopardizes replicability: lessons learned across disciplines. Royal Society Open Science. 2021;8:201925. doi: 10.1098/rsos.201925 33996122 PMC8059606

[7] Simmons JP, Nelson LD, Simonsohn U. False-positive psychology: undisclosed flexibility in data collection and analysis allows presenting anything as significant. Psychological Science. 2011;22(11):1359–1366. doi: 10.1177/0956797611417632 22006061

[8] Klau S, Martin-Magniette ML, Boulesteix AL, Hoffmann S. Sampling uncertainty versus method uncertainty: A general framework with applications to omics biomarker selection. Biometrical Journal. 2020;62(3):670–687. doi: 10.1002/bimj.201800309 31099917

[9] Peschel S, Müller CL, von Mutius E, Boulesteix AL, Depner M. NetCoMi: network construction and comparison for microbiome data in R. Briefings in Bioinformatics. 2020;22(4):bbaa290. doi: 10.1093/bib/bbaa290 PMC8293835 33264391

[10] Nosek BA, Errington TM. What is replication? PLoS Biology. 2020;18(3):e3000691. doi: 10.1371/journal.pbio.3000691 32218571 PMC7100931

[11] Ullmann T, Hennig C, Boulesteix AL. Validation of cluster analysis results on validation data: A systematic framework. Wiley Interdisciplinary Reviews: Data Mining and Knowledge Discovery. 2022;12(3):e1444. doi: 10.1002/widm.1444

[12] Ioannidis JP. Why most published research findings are false. PLoS Medicine. 2005;2(8):e124. doi: 10.1371/journal.pmed.0020124 16060722 PMC1182327

[13] Gelman A, Loken E. The statistical crisis in science. American Scientist. 2014;102(6):460. doi: 10.1511/2014.111.460

[14] Head ML, Holman L, Lanfear R, Kahn AT, Jennions MD. The extent and consequences of p-hacking in science. PLoS Biology. 2015;13(3):e1002106. doi: 10.1371/journal.pbio.1002106 25768323 PMC4359000

[15] McDonald D, Hyde E, Debelius JW, Morton JT, Gonzalez A, Ackermann G, et al. American gut: an open platform for citizen science microbiome research. Msystems. 2018;3(3):e00031–18. doi: 10.1128/mSystems.00031-18 29795809 PMC5954204

[16] Sinha R, Abu-Ali G, Vogtmann E, Fodor AA, Ren B, Amir A, et al. Assessment of variation in microbial community amplicon sequencing by the Microbiome Quality Control (MBQC) project consortium. Nature Biotechnology. 2017;35(11):1077–1086. doi: 10.1038/nbt.3981 28967885 PMC5839636

[17] Allali I, Arnold JW, Roach J, Cadenas MB, Butz N, Hassan HM, et al. A comparison of sequencing platforms and bioinformatics pipelines for compositional analysis of the gut microbiome. BMC Microbiology. 2017;17(1):194. doi: 10.1186/s12866-017-1101-8 28903732 PMC5598039

[18] Clausen DS, Willis AD. Evaluating replicability in microbiome data. Biostatistics. 2021;kxab048. doi: 10.1093/biostatistics/kxab048 PMC9566336 34969071

[19] Tierney BT, Tan Y, Yang Z, Shui B, Walker MJ, Kent BM, et al. Systematically assessing microbiome–disease associations identifies drivers of inconsistency in metagenomic research. PLoS Biology. 2022;20(3):1–18. doi: 10.1371/journal.pbio.3001556 35235560 PMC8890741

[20] Nearing JT, Douglas GM, Hayes MG, MacDonald J, Desai DK, Allward N, et al. Microbiome differential abundance methods produce different results across 38 datasets. Nature Communications. 2022;13(1):1–16. doi: 10.1038/s41467-022-28034-z PMC8763921 35039521

[21] Khomich M, Måge I, Rud I, Berget I. Analysing microbiome intervention design studies: Comparison of alternative multivariate statistical methods. PLoS One. 2021;16(11):1–20. doi: 10.1371/journal.pone.0259973 34793531 PMC8601541

[22] Hubert L, Arabie P. Comparing partitions. Journal of Classification. 1985;2(1):193–218. doi: 10.1007/BF01908075

[23] Badri M, Kurtz ZD, Bonneau R, Müller CL. Shrinkage improves estimation of microbial associations under different normalization methods. NAR Genomics and Bioinformatics. 2020;2(4):lqaa100. doi: 10.1093/nargab/lqaa100 33575644 PMC7745771

[24] Berry D, Widder S. Deciphering microbial interactions and detecting keystone species with co-occurrence networks. Frontiers in Microbiology. 2014;5:219. doi: 10.3389/fmicb.2014.00219 24904535 PMC4033041

[25] Agler MT, Ruhe J, Kroll S, Morhenn C, Kim ST, Weigel D, et al. Microbial hub taxa link host and abiotic factors to plant microbiome variation. PLoS Biology. 2016;14(1):e1002352. doi: 10.1371/journal.pbio.1002352 26788878 PMC4720289

[26] Banerjee S, Schlaeppi K, van der Heijden MG. Keystone taxa as drivers of microbiome structure and functioning. Nature Reviews Microbiology. 2018;16(9):567–576. doi: 10.1038/s41579-018-0024-1 29789680

[27] Röttjers L, Faust K. From hairballs to hypotheses–biological insights from microbial networks. FEMS Microbiology Reviews. 2018;42(6):761–780. doi: 10.1093/femsre/fuy030 30085090 PMC6199531

[28] Zamkovaya T, Foster JS, de Crécy-Lagard V, Conesa A. A network approach to elucidate and prioritize microbial dark matter in microbial communities. The ISME Journal. 2021;15(1):228–244. doi: 10.1038/s41396-020-00777-x 32963345 PMC7852563

[29] Francino M. Antibiotics and the human gut microbiome: dysbioses and accumulation of resistances. Frontiers in microbiology. 2016;6:1543. doi: 10.3389/fmicb.2015.01543 26793178 PMC4709861

[30] Costello EK, Stagaman K, Dethlefsen L, Bohannan BJ, Relman DA. The application of ecological theory toward an understanding of the human microbiome. Science. 2012;336(6086):1255–1262. doi: 10.1126/science.1224203 22674335 PMC4208626

[31] Yaveroğlu ÖN, Malod-Dognin N, Davis D, Levnajic Z, Janjic V, Karapandza R, et al. Revealing the hidden language of complex networks. Scientific Reports. 2014;4(1):1–9. doi: 10.1038/srep04547 24686408 PMC3971399

[32] Mahana D, Trent CM, Kurtz ZD, Bokulich NA, Battaglia T, Chung J, et al. Antibiotic perturbation of the murine gut microbiome enhances the adiposity, insulin resistance, and liver disease associated with high-fat diet. Genome Medicine. 2016;8(1):1–20. doi: 10.1186/s13073-016-0297-9 27124954 PMC4847194

[33] Ruiz VE, Battaglia T, Kurtz ZD, Bijnens L, Ou A, Engstrand I, et al. A single early-in-life macrolide course has lasting effects on murine microbial network topology and immunity. Nature Communications. 2017;8(1):1–14. doi: 10.1038/s41467-017-00531-6 28894149 PMC5593929

[34] Leung MH, Tong X, Wilkins D, Cheung HH, Lee PK. Individual and household attributes influence the dynamics of the personal skin microbiota and its association network. Microbiome. 2018;6(1):1–15. doi: 10.1186/s40168-018-0412-9 29394957 PMC5797343

[35] Arumugam M, Raes J, Pelletier E, Le Paslier D, Yamada T, Mende DR, et al. Enterotypes of the human gut microbiome. Nature. 2011;473:174–180. doi: 10.1038/nature09944 21508958 PMC3728647

[36] Jeffery IB, Claesson MJ, O’Toole PW, Shanahan F. Categorization of the gut microbiota: enterotypes or gradients? Nature Reviews Microbiology. 2012;10(9):591–592. doi: 10.1038/nrmicro2859 23066529

[37] Koren O, Knights D, Gonzalez A, Waldron L, Segata N, Knight R, et al. A guide to enterotypes across the human body: meta-analysis of microbial community structures in human microbiome datasets. PLoS Computational Biology. 2013;9(1):e1002863. doi: 10.1371/journal.pcbi.1002863 23326225 PMC3542080

[38] Knights D, Ward TL, McKinlay CE, Miller H, Gonzalez A, McDonald D, et al. Rethinking “enterotypes”. Cell Host & Microbe. 2014;16(4):433–437. doi: 10.1016/j.chom.2014.09.013 25299329 PMC5558460

[39] Costea PI, Hildebrand F, Arumugam M, Bäckhed F, Blaser MJ, Bushman FD, et al. Enterotypes in the landscape of gut microbial community composition. Nature Microbiology. 2018;3:8–16. doi: 10.1038/s41564-017-0072-8 29255284 PMC5832044

[40] Cheng M, Ning K. Stereotypes about enterotype: the old and new ideas. Genomics, Proteomics & Bioinformatics. 2019;17(1):4–12. doi: 10.1016/j.gpb.2018.02.004 31026581 PMC6521238

[41] Wu GD, Chen J, Hoffmann C, Bittinger K, Chen YY, Keilbaugh SA, et al. Linking long-term dietary patterns with gut microbial enterotypes. Science. 2011;334(6052):105–108. doi: 10.1126/science.1208344 21885731 PMC3368382

[42] Rousseeuw PJ. Silhouettes: a graphical aid to the interpretation and validation of cluster analysis. Journal of Computational and Applied Mathematics. 1987;20:53–65. doi: 10.1016/0377-0427(87)90125-7

[43] Aitchison J. The statistical analysis of compositional data. Journal of the Royal Statistical Society: Series B (Methodological). 1982;44(2):139–160.

[44] Yoon G, Gaynanova I, Müller CL. Microbial networks in SPRING—Semi-parametric rank-based correlation and partial correlation estimation for quantitative microbiome data. Frontiers in Genetics. 2019;10:516. doi: 10.3389/fgene.2019.00516 31244881 PMC6563871

[45] Anders S, Huber W. Differential expression analysis for sequence count data. Genome Biology. 2010;11:R106. doi: 10.1186/gb-2010-11-10-r106 20979621 PMC3218662

[46] Yoon G, Carroll RJ, Gaynanova I. Sparse semiparametric canonical correlation analysis for data of mixed types. Biometrika. 2020;107(3):609–625. doi: 10.1093/biomet/asaa007 34621080 PMC8494134

[47] Lovell D, Pawlowsky-Glahn V, Egozcue JJ, Marguerat S, Bähler J. Proportionality: a valid alternative to correlation for relative data. PLoS Computational Biology. 2015;11(3):e1004075. doi: 10.1371/journal.pcbi.1004075 25775355 PMC4361748

[48] Ng A, Jordan M, Weiss Y. On spectral clustering: analysis and an algorithm. Advances in Neural Information Processing Systems. 2001;14:849–856.

[49] Clauset A, Newman ME, Moore C. Finding community structure in very large networks. Physical Review E. 2004;70(6):066111. doi: 10.1103/PhysRevE.70.066111 15697438

[50] Blondel VD, Guillaume JL, Lambiotte R, Lefebvre E. Fast unfolding of communities in large networks. Journal of Statistical Mechanics: Theory and Experiment. 2008;2008(10):P10008. doi: 10.1088/1742-5468/2008/10/P10008

[51] Röttjers L, Faust K. Manta: A clustering algorithm for weighted ecological networks. Msystems. 2020;5(1):e00903–19. doi: 10.1128/mSystems.00903-19 32071163 PMC7029223

[52] Aitchison J. On criteria for measures of compositional difference. Mathematical Geology. 1992;24(4):365–379. doi: 10.1007/BF00891269

[53] Martín-Fernández JA, Bren M, Barceló-Vidal C, Pawlowsky-Glahn V. A measure of difference for compositional data based on measures of divergence. In: Proceedings of the Fifth Annual Conference of the International Association for Mathematical Geology. vol. 1; 1999. p. 211–215.

[54] Bray JR, Curtis JT. An ordination of the upland forest communities of southern Wisconsin. Ecological Monographs. 1957;27(4):326–349. doi: 10.2307/1942268

[55] Holmes I, Harris K, Quince C. Dirichlet multinomial mixtures: generative models for microbial metagenomics. PloS One. 2012;7(2):e30126. doi: 10.1371/journal.pone.0030126 22319561 PMC3272020

[56] Kaufman L, Rousseeuw PJ. Finding Groups in Data. John Wiley & Sons, Ltd; 1990.

[57] Boulesteix AL, Lauer S, Eugster MJ. A plea for neutral comparison studies in computational sciences. PloS One. 2013;8(4):e61562. doi: 10.1371/journal.pone.0061562 23637855 PMC3634809

[58] Boulesteix AL, Wilson R, Hapfelmeier A. Towards evidence-based computational statistics: lessons from clinical research on the role and design of real-data benchmark studies. BMC Medical Research Methodology. 2017;17:138. doi: 10.1186/s12874-017-0417-2 28888225 PMC5591542

[59] Wallen ZD. Comparison study of differential abundance testing methods using two large Parkinson disease gut microbiome datasets derived from 16S amplicon sequencing. BMC Bioinformatics. 2021;22(1):1–29. doi: 10.1186/s12859-021-04193-6 34034646 PMC8147401

[60] Bokulich NA, Ziemski M, Robeson MS II, Kaehler BD. Measuring the microbiome: Best practices for developing and benchmarking microbiomics methods. Computational and Structural Biotechnology Journal. 2020;18:4048–4062. doi: 10.1016/j.csbj.2020.11.049 33363701 PMC7744638

[61] Nosek BA, Ebersole CR, DeHaven AC, Mellor DT. The preregistration revolution. Proceedings of the National Academy of Sciences. 2018;115(11):2600–2606. doi: 10.1073/pnas.1708274114 29531091 PMC5856500

[62] Wirbel J, Zych K, Essex M, Karcher N, Kartal E, Salazar G, et al. Microbiome meta-analysis and cross-disease comparison enabled by the SIAMCAT machine learning toolbox. Genome Biology. 2021;22:93. doi: 10.1186/s13059-021-02306-1 33785070 PMC8008609

[63] Bien J, Yan X, Simpson L, Müller CL. Tree-aggregated predictive modeling of microbiome data. Scientific Reports. 2021;11(1):1–13. doi: 10.1038/s41598-021-93645-3 34267244 PMC8282688

[64] Fromentin S, Forslund SK, Chechi K, Aron-Wisnewsky J, Chakaroun R, Nielsen T, et al. Microbiome and metabolome features of the cardiometabolic disease spectrum. Nature Medicine. 2022;28:303–314. doi: 10.1038/s41591-022-01688-4 35177860 PMC8863577

[65] Palmer C, Bik EM, DiGiulio DB, Relman DA, Brown PO. Development of the human infant intestinal microbiota. PLoS Biology. 2007;5(7):e177. doi: 10.1371/journal.pbio.0050177 17594176 PMC1896187

[66] Claesson MJ, Cusack S, O’Sullivan O, Greene-Diniz R, de Weerd H, Flannery E, et al. Composition, variability, and temporal stability of the intestinal microbiota of the elderly. Proceedings of the National Academy of Sciences. 2011;108:4586–4591. doi: 10.1073/pnas.1000097107 20571116 PMC3063589

[67] Derrien M, Alvarez AS, de Vos WM. The gut microbiota in the first decade of life. Trends in Microbiology. 2019;27(12):997–1010. doi: 10.1016/j.tim.2019.08.001 31474424

[68] Zhong H, Penders J, Shi Z, Ren H, Cai K, Fang C, et al. Impact of early events and lifestyle on the gut microbiota and metabolic phenotypes in young school-age children. Microbiome. 2019;7:2. doi: 10.1186/s40168-018-0608-z 30609941 PMC6320620

[69] Yoon G, Müller CL, Gaynanova I. Fast computation of latent correlations. Journal of Computational and Graphical Statistics. 2021;30(4):1249–1256. doi: 10.1080/10618600.2021.1882468 35280976 PMC8916743

[70] Quinn TP, Richardson MF, Lovell D, Crowley TM. propr: an R-package for identifying proportionally abundant features using compositional data analysis. Scientific Reports. 2017;7(1):1–9. doi: 10.1038/s41598-017-16520-0 29176663 PMC5701231

[71] Meinshausen N, Bühlmann P. High-dimensional graphs and variable selection with the lasso. Annals of Statistics. 2006;34(3):1436–1462. doi: 10.1214/009053606000000281

[72] Efron B. Local False Discovery Rates. Stanford University; 2005.

[73] Freeman LC. Centrality in social networks conceptual clarification. Social networks. 1978;1(3):215–239. doi: 10.1016/0378-8733(78)90021-7

[74] Jaccard P. The distribution of the flora in the alpine zone. New Phytologist. 1912;11(2):37–50. doi: 10.1111/j.1469-8137.1912.tb05611.x

[75] Ding T, Schloss PD. Dynamics and associations of microbial community types across the human body. Nature. 2014;509(7500):357–360. doi: 10.1038/nature13178 24739969 PMC4139711

[76] Csárdi G, Nepusz T. The igraph software package for complex network research. InterJournal. 2006;Complex Systems:1695.

[77] Ushey K, Allaire J, Tang Y. reticulate: interface to’Python’; 2022. Available from: https://rstudio.github.io/reticulate/.

[78] Maechler M, Rousseeuw P, Struyf A, Hubert M, Hornik K. cluster: cluster analysis basics and extensions; 2022. Available from: https://CRAN.R-project.org/package=cluster.

[79] Morgan M. DirichletMultinomial: Dirichlet-multinomial mixture model machine learning for microbiome data; 2022. Available from: https://www.bioconductor.org/packages/release/bioc/html/DirichletMultinomial.html.

[80] Hočevar T, Demšar J. Computation of graphlet orbits for nodes and edges in sparse graphs. Journal of Statistical Software. 2016;71(10):1–24.

