# Supplementary material for: Over-optimism in unsupervised microbiome analysis: Insights from network learning and clustering
Source: PLoS Comput Biol. 2023 Jan 6;19(1):e1010820. doi: 10.1371/journal.pcbi.1010820 (PMC9873197; doi:10.1371/journal.pcbi.1010820)
Supplement: S1 Text — (PDF) [file pcbi.1010820.s001.pdf]

S1: Full results and plots for research task 1 (clustering of bacterial genera)

List of Figures

A Results for clustering bacterial genera on the discovery data, $n = 100$ . . . 2
B Results for clustering bacterial genera on the discovery data, $n = 250$ . . . 3
C Results for clustering bacterial genera on the discovery data, $n = 500$ . . . 3
D Results for clustering bacterial genera on the discovery data, $n = 1000$ . . . 4
E Results for clustering bacterial genera on the discovery data, $n = 4000$ . . . 4
F Results for network-based clustering of bacterial genera on the discovery data, separated by sparsification methods, $n = 100$ . . . 5
G Results for network-based clustering of bacterial genera on the discovery data, separated by sparsification methods, $n = 250$ . . . 6
H Results for network-based clustering of bacterial genera on the discovery data, separated by sparsification methods, $n = 500$ . . . 6
I Results for network-based clustering of bacterial genera on the discovery data, separated by sparsification methods, $n = 1000$ . . . 7
J Results for network-based clustering of bacterial genera on the discovery data, separated by sparsification methods, $n = 4000$ . . . 7
K Best ARIs for the clustering of bacterial genera on the discovery data, compared with the results on validation data, $n = 100$ . . . 8
L Best ARIs for the clustering of bacterial genera on the discovery data, compared with the results on validation data, $n = 250$ . . . 8
M Best ARIs for the clustering of bacterial genera on the discovery data, compared with the results on validation data, $n = 500$ . . . 9
N Best ARIs for the clustering of bacterial genera on the discovery data, compared with the results on validation data, $n = 1000$ . . . 9
O Best ARIs for the clustering of bacterial genera on the discovery data, compared with the results on validation data, $n = 4000$ . . . 10

Fig A-E show the results of applying different clustering methods to the discovery data for sample sizes $n \in \{100, 250, 500, 1000, 4000\}$. For each method combination on the x-axis, the resulting ARIs, which measure agreement with the taxonomic categorization into families, are summarized over the 50 samplings by boxplots. Outliers are indicated by black crosses. Additionally, all results are shown as colored dots, with the color indicating the number k of clusters in the respective clustering result. Results that were picked as the "best result" in one of the 50 samplings are marked by red square edges. For the network-based clustering methods with the networks generated by either the Pearson or Spearman correlation, the results for t-test and threshold sparsification are displayed together, i.e., $50*2 = 100$ results are shown for these method combinations.

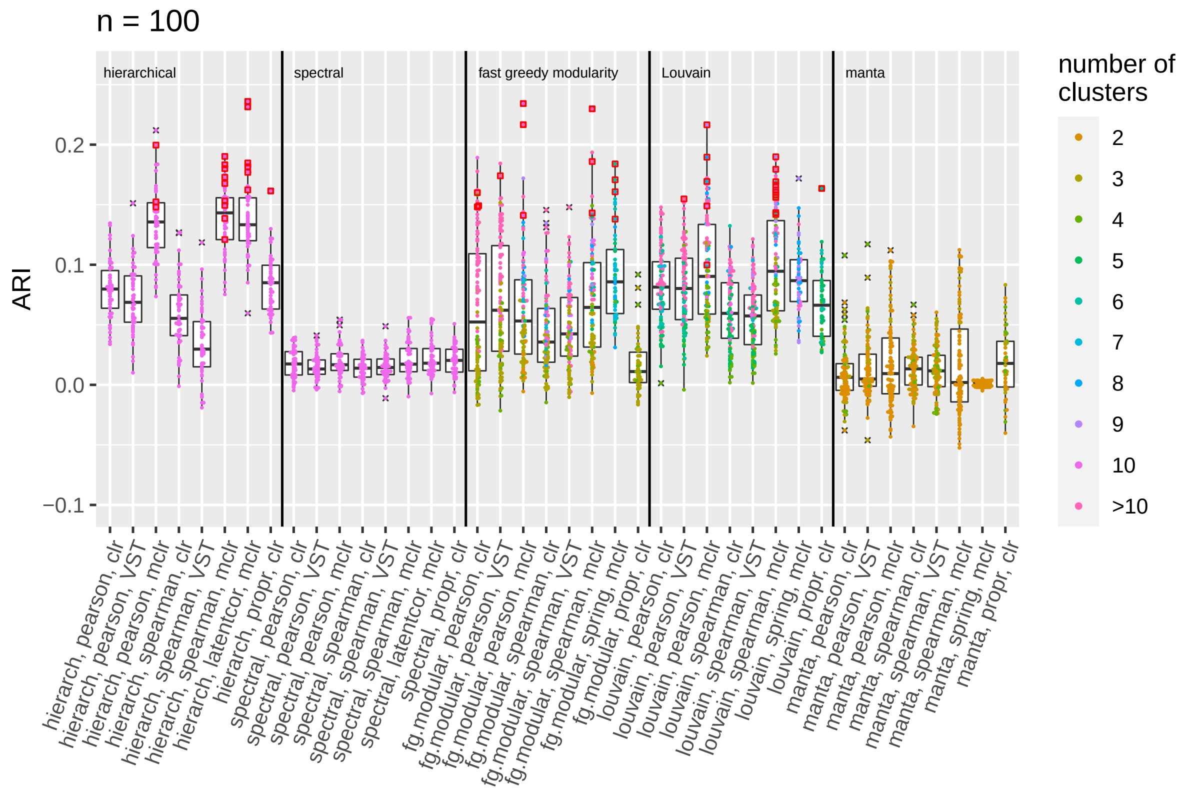

Fig A. Results for clustering bacterial genera on the discovery data, $n = 100$

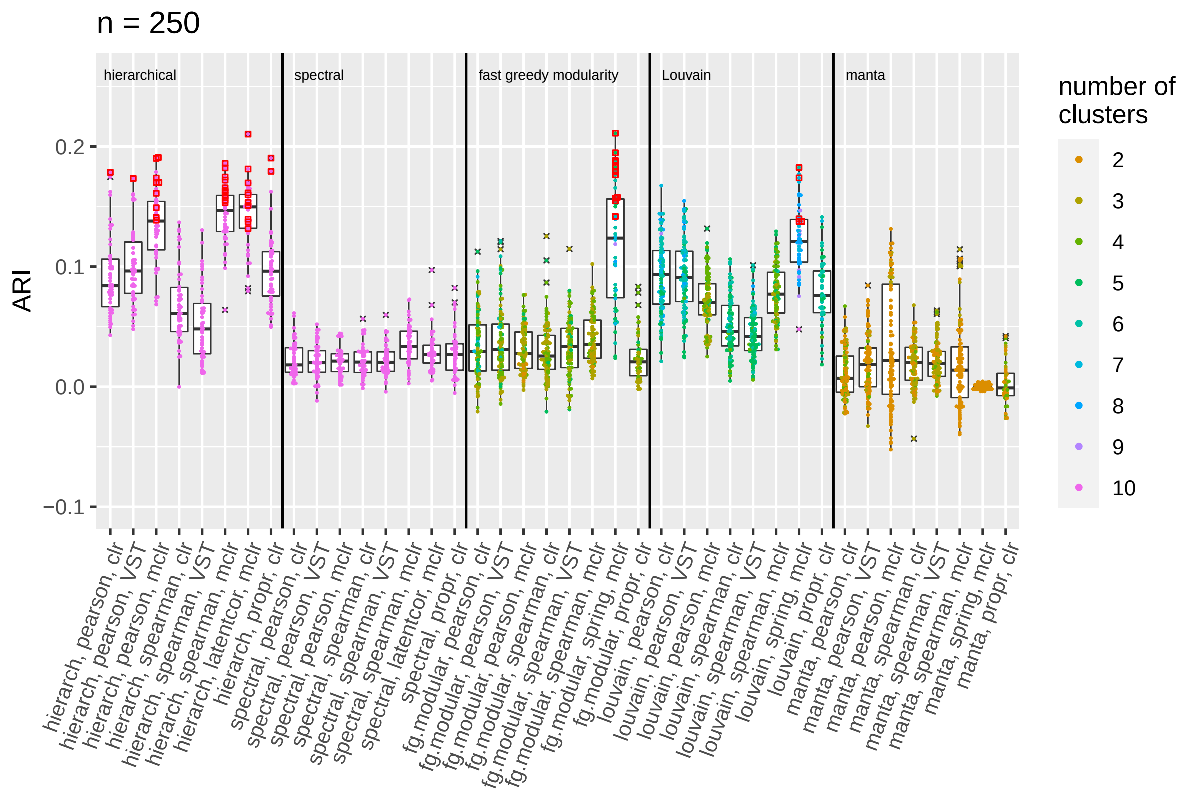

Fig B. Results for clustering bacterial genera on the discovery data, $n = 250$

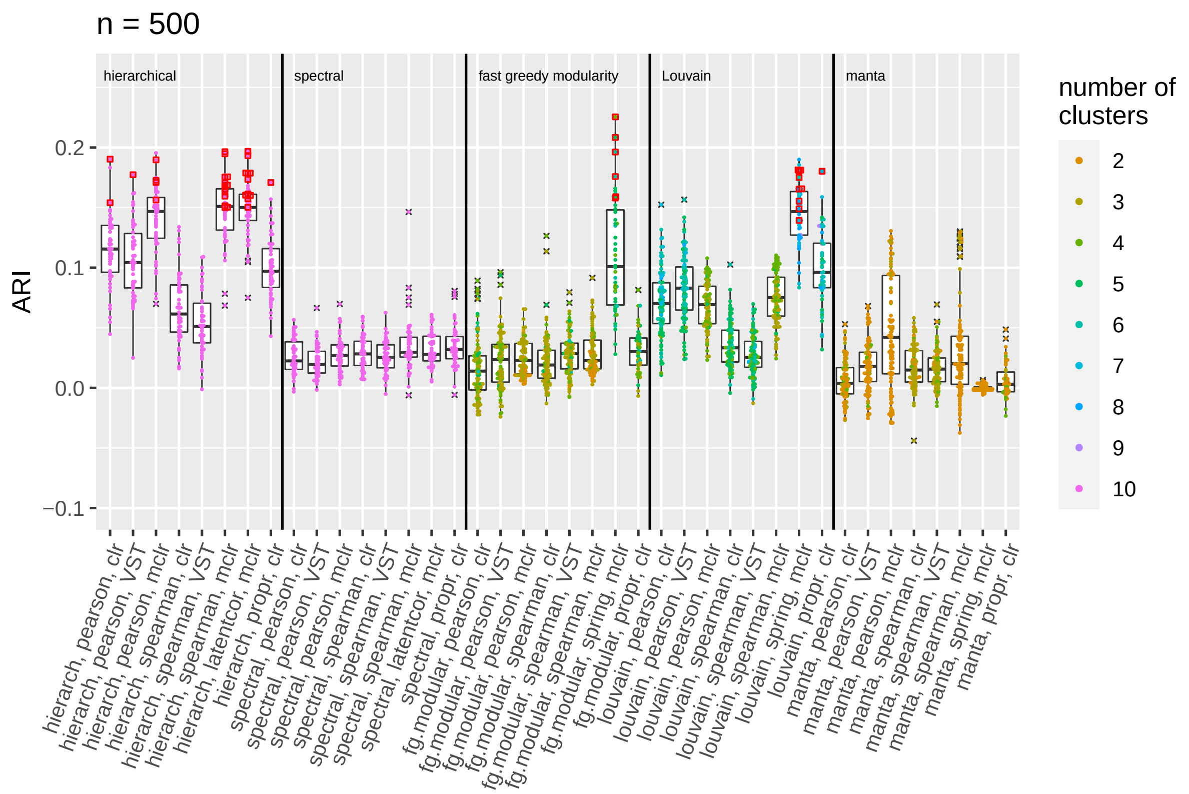

Fig C. Results for clustering bacterial genera on the discovery data, $n = 500$

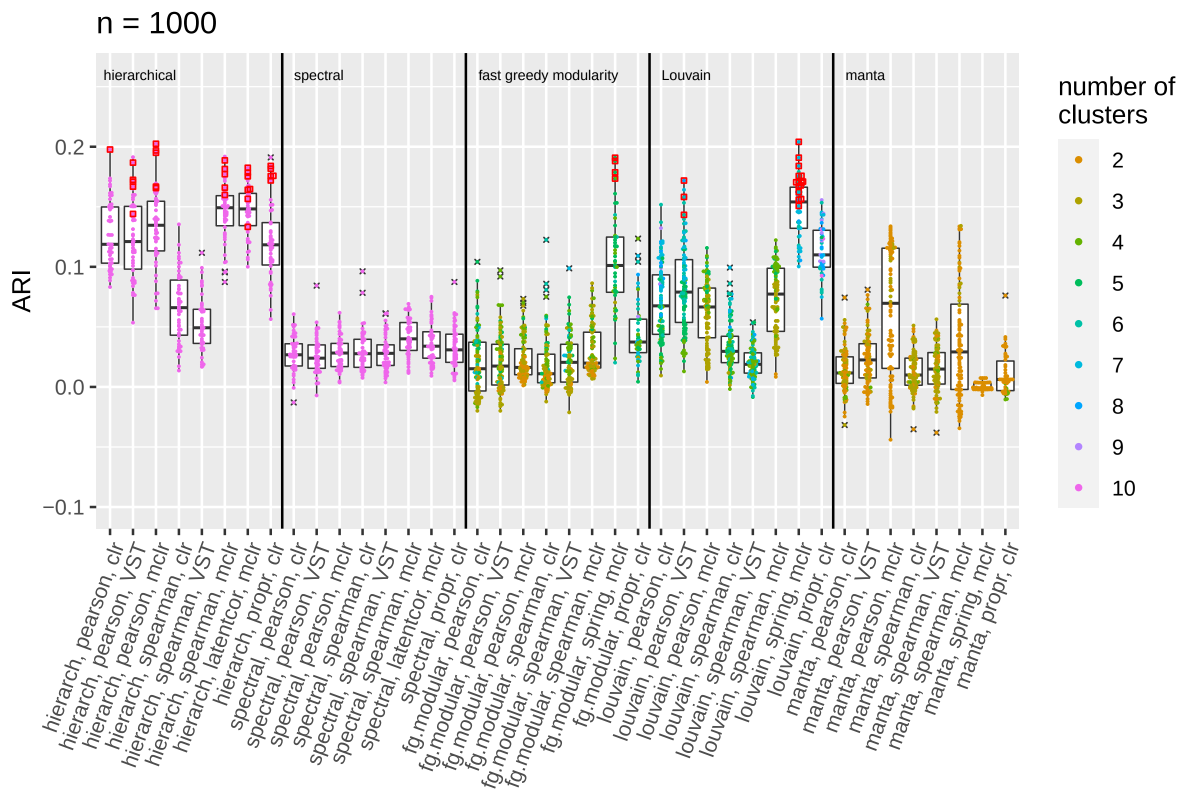

Fig D. Results for clustering bacterial genera on the discovery data, $n = 1000$

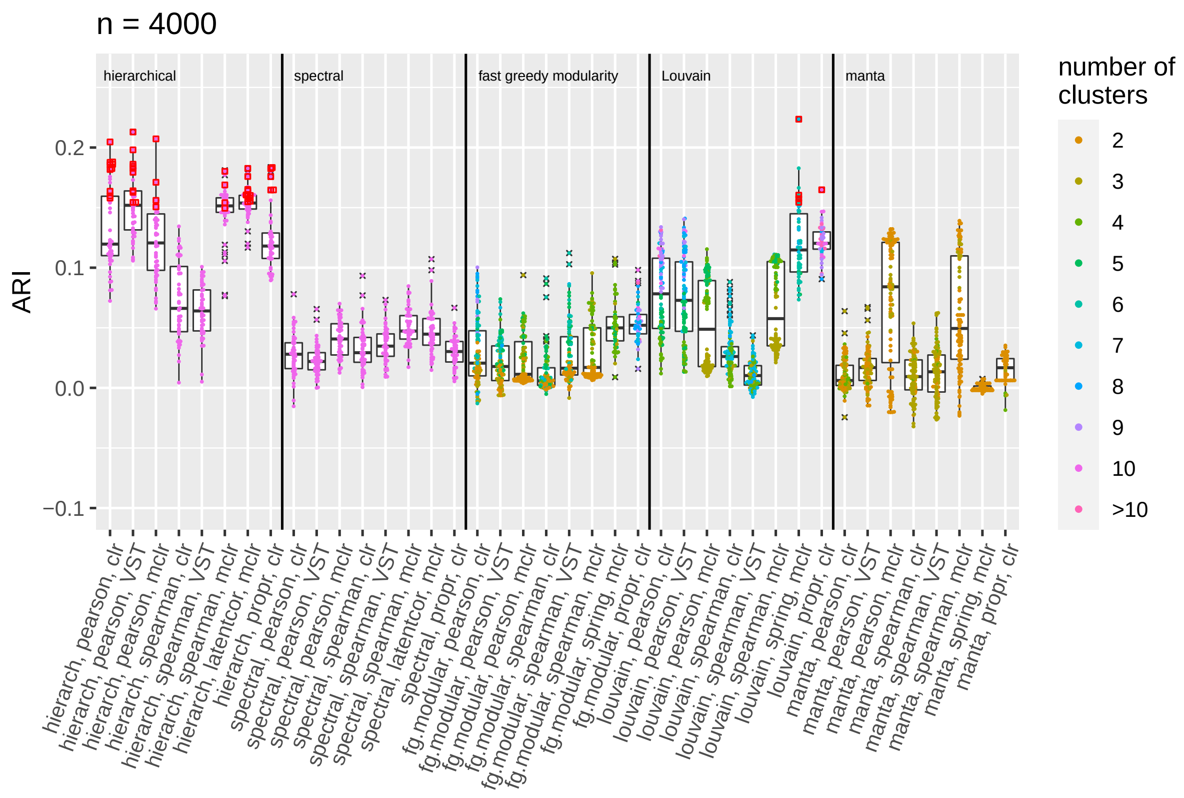

Fig E. Results for clustering bacterial genera on the discovery data, $n = 4000$

As can be seen in Fig A-E, the best ARI results stem either from hierarchical clustering, the Louvain method or fast greedy modularity optimization. Spectral clustering and manta are never selected. There is some change in the selected “best” methods with respect to sample size. For example, for $n = 100$, fast greedy modularity clustering performs well in several of the 50 samplings, but this cluster method does not yield very good ARI results for $n = 4000$. At $n = 4000$, hierarchical clustering is chosen as the best method in 45 of the 50 samplings.

In Fig F-J, results for the network-based clustering are shown separately for both sparsification methods (t-test and threshold). Results that were picked as the “best result” in one of the 50 samplings are marked by red square edges.

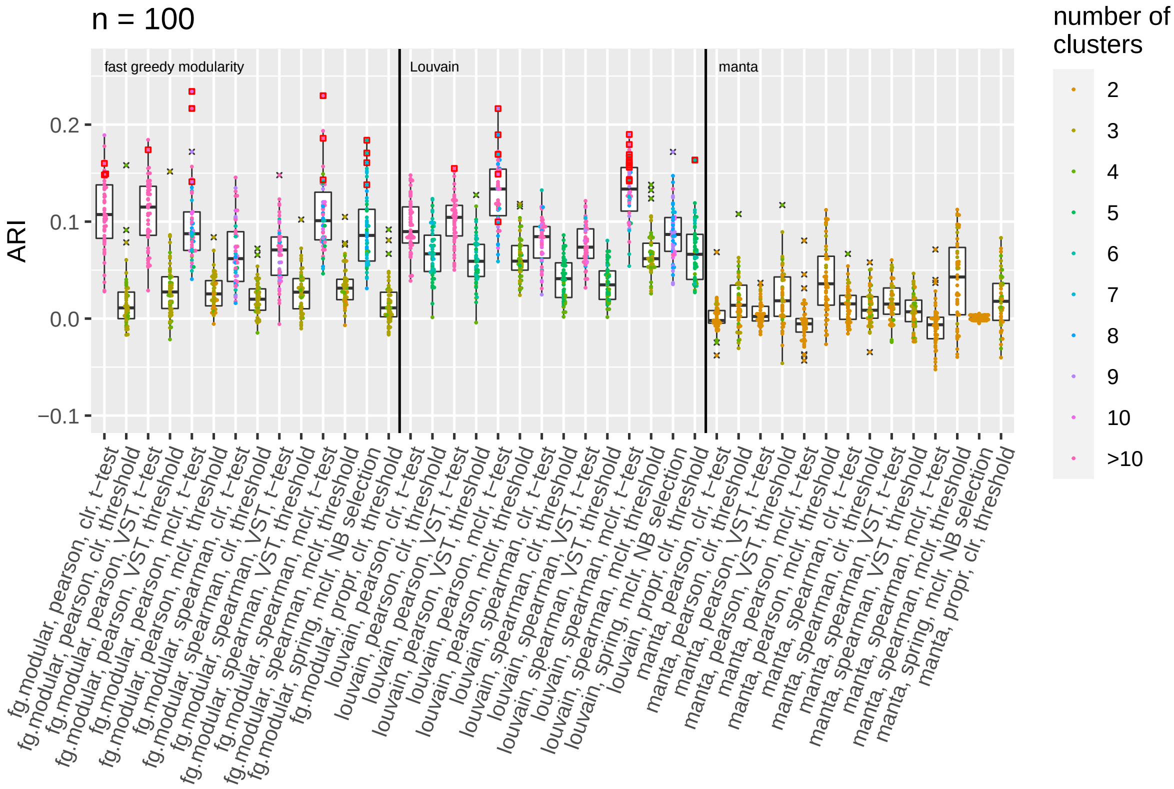

Fig F. Results for network-based clustering of bacterial genera on the discovery data, separated by sparsification methods, $n = 100$

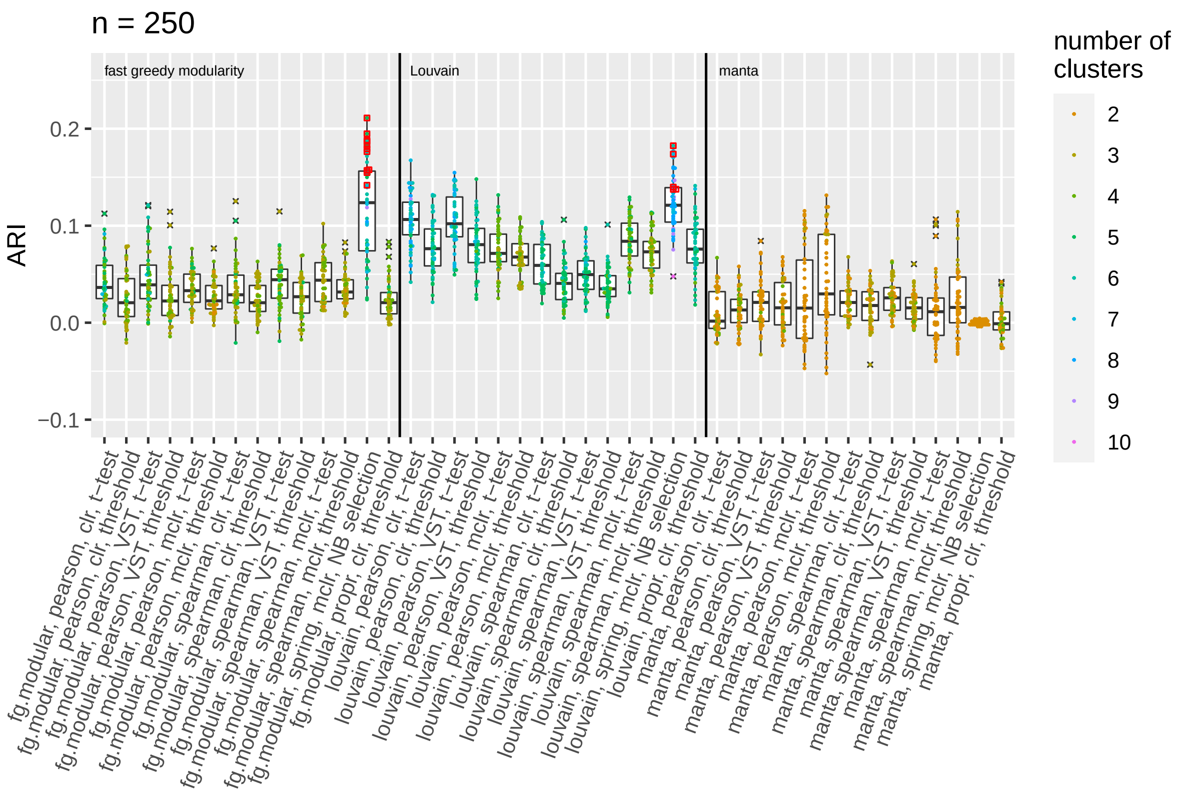

Fig G. Results for network-based clustering of bacterial genera on the discovery data, separated by sparsification methods, $n = 250$

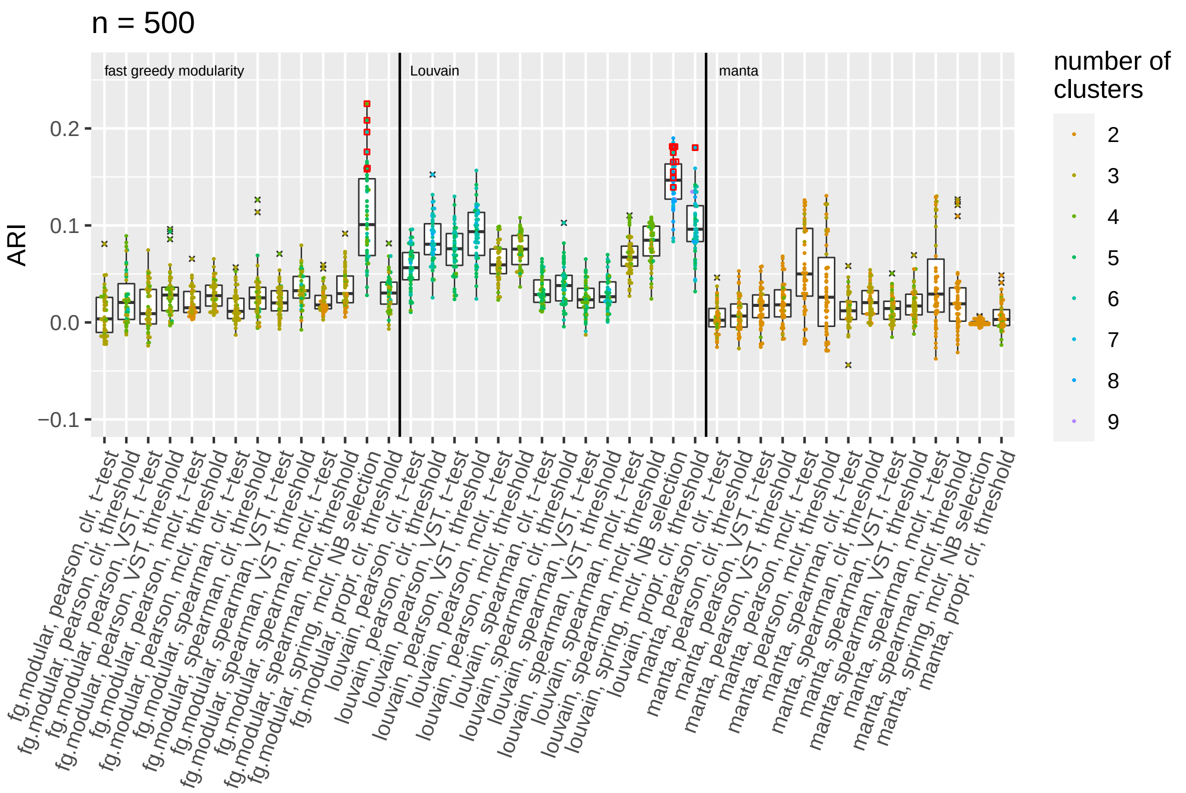

Fig H. Results for network-based clustering of bacterial genera on the discovery data, separated by sparsification methods, $n = 500$

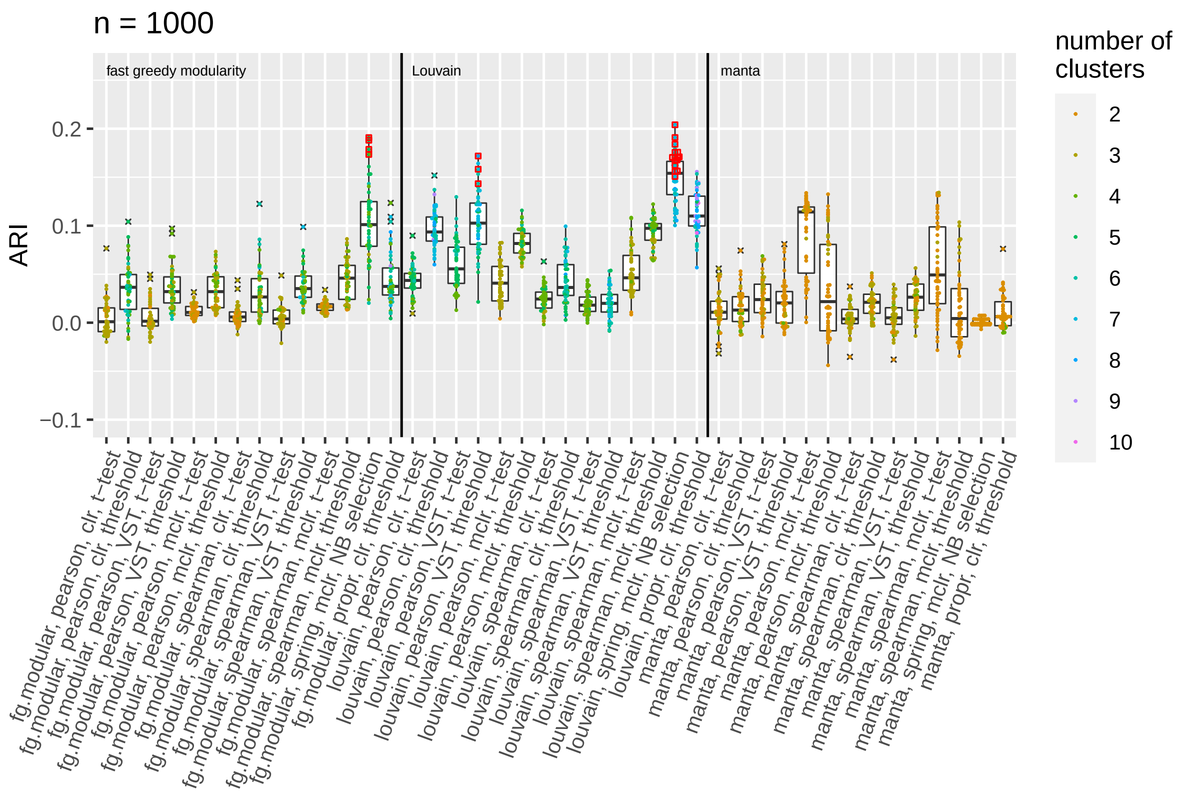

Fig I. Results for network-based clustering of bacterial genera on the discovery data, separated by sparsification methods, $n = 1000$

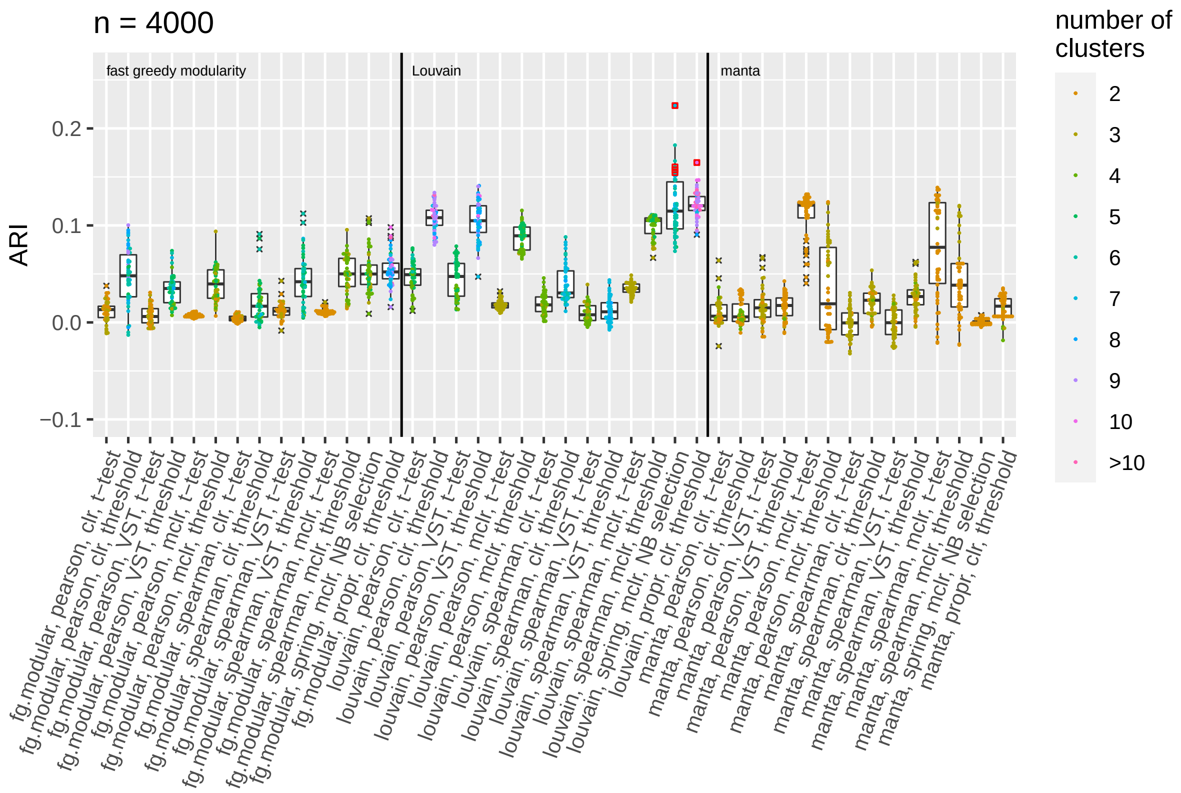

Fig J. Results for network-based clustering of bacterial genera on the discovery data, separated by sparsification methods, $n = 4000$

Our main interest lies in applying the "best" method to the validation data and checking whether the ARI result can be validated. The results are shown in Fig K-O. On the x-axis, the method combinations that were best in at least one of the 50 samplings are shown. The ARI values are shown as colored dots, with the color indicating the number k of clusters in the respective clustering result.

For each of the 50 samplings, the respective best method combination is applied to the validation data. The ARI value on the discovery data (belonging to the best method combination) and the corresponding ARI on the validation data are connected by lines. The lines point downwards in most cases, i.e., the results for the validation data are usually slightly worse than for the discovery data.

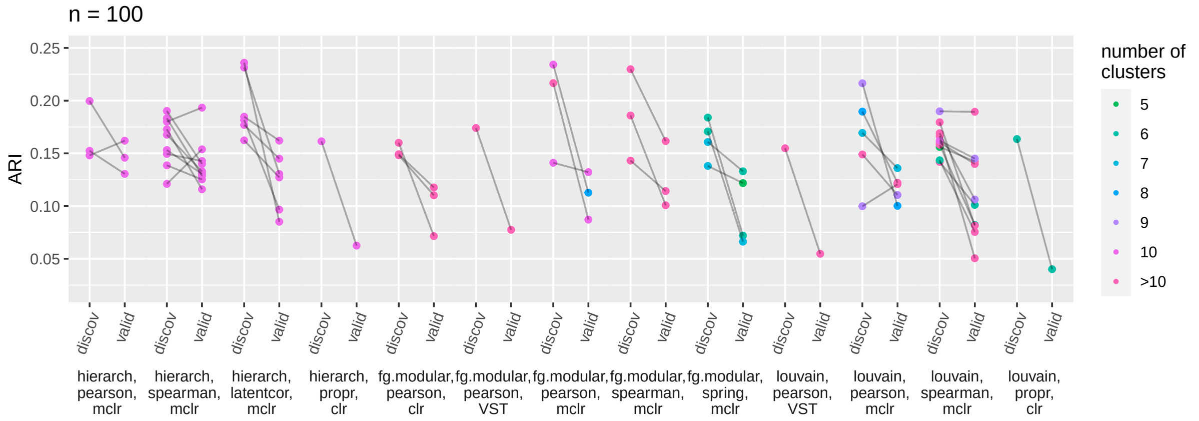

Fig K. Best ARIs for the clustering of bacterial genera on the discovery data, compared with the results on validation data, $n = 100$

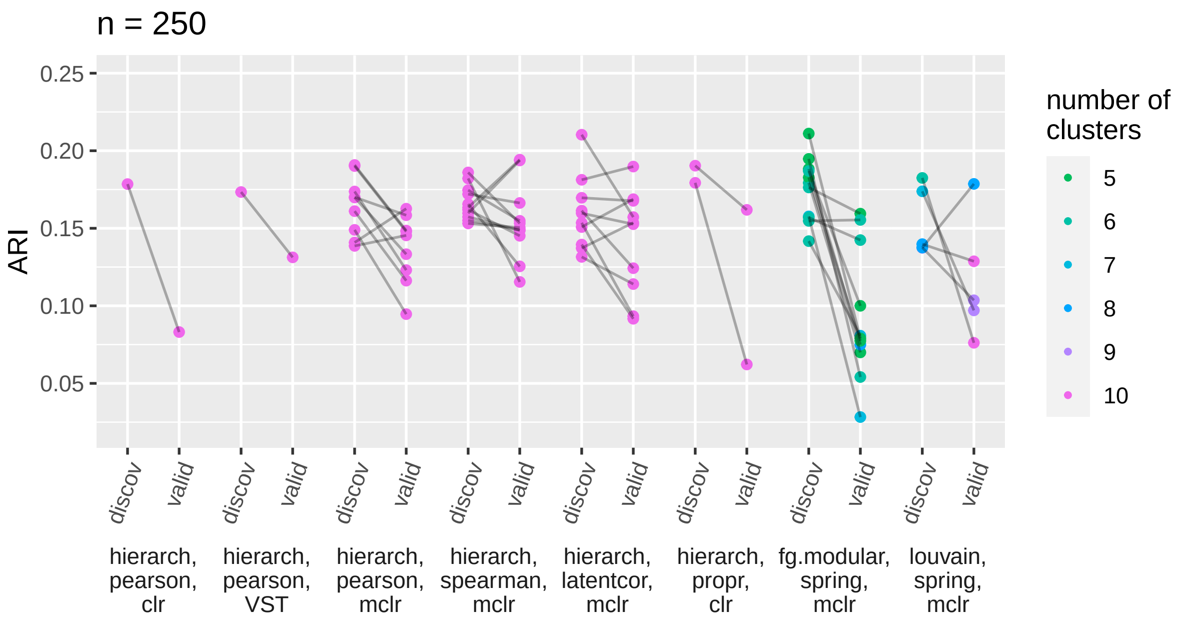

Fig L. Best ARIs for the clustering of bacterial genera on the discovery data, compared with the results on validation data, $n = 250$

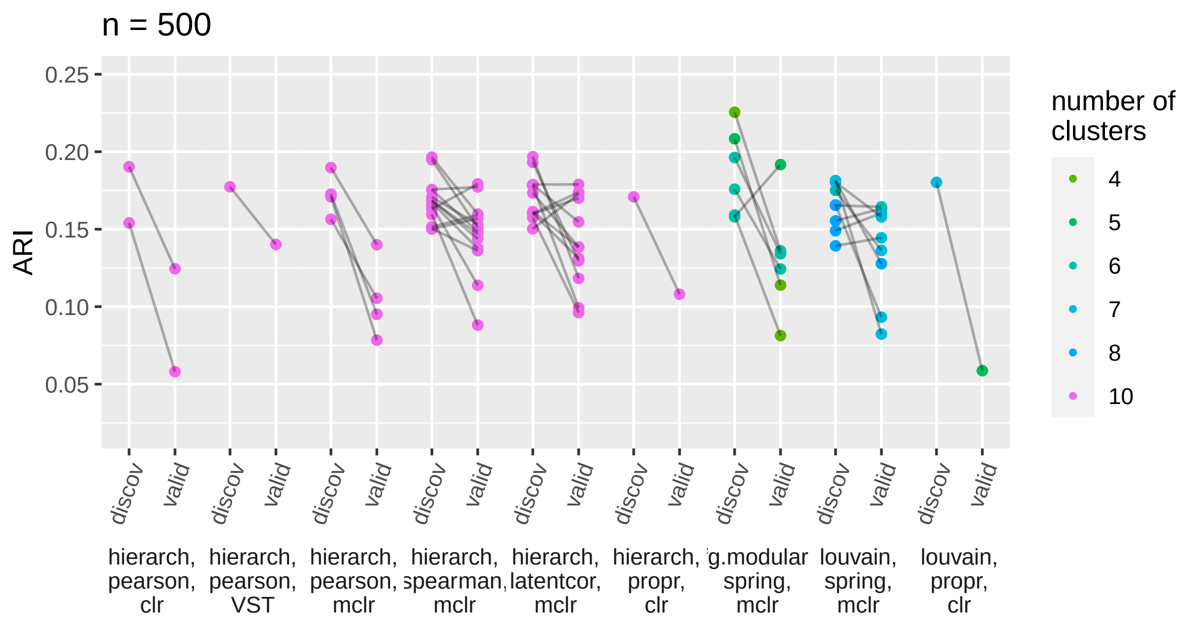

Fig M. Best ARIs for the clustering of bacterial genera on the discovery data, compared with the results on validation data, $n = 500$

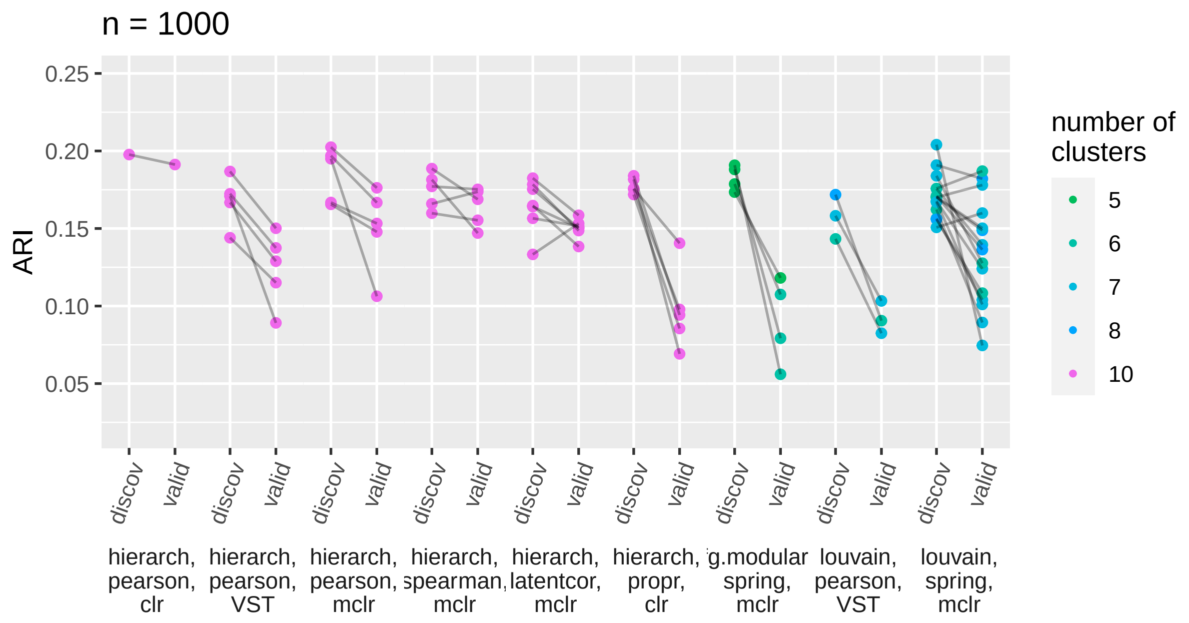

Fig N. Best ARIs for the clustering of bacterial genera on the discovery data, compared with the results on validation data, $n = 1000$

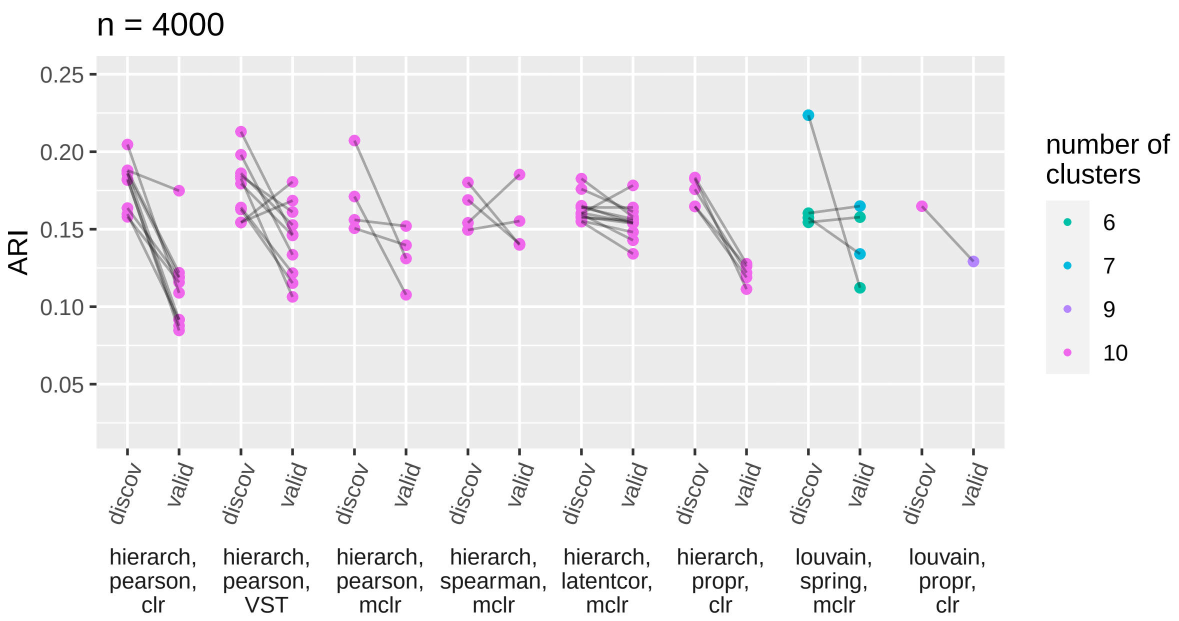

Fig O. Best ARIs for the clustering of bacterial genera on the discovery data, compared with the results on validation data, $n = 4000$
