# Supplementary material for: Over-optimism in unsupervised microbiome analysis: Insights from network learning and clustering
Source: PLoS Comput Biol. 2023 Jan 6;19(1):e1010820. doi: 10.1371/journal.pcbi.1010820 (PMC9873197; doi:10.1371/journal.pcbi.1010820)
Supplement: S2 Text — (PDF) [file pcbi.1010820.s002.pdf]

S2: Full results and plots for research task 2 (hub detection)

List of Figures

A Results for hub detection on the discovery data, $n = 100$ 2
B Results for hub detection on the discovery data, $n = 250$ 3
C Results for hub detection on the discovery data, $n = 500$ 4
D Results for hub detection on the discovery data, $n = 1000$ 5
E Results for hub detection on the discovery data, $n = 4000$ 6
F Highest numbers of hubs for the hub detection on the discovery data, compared with the results on validation data, $n = 100$ 7
G Highest numbers of hubs for the hub detection on the discovery data, compared with the results on validation data, $n = 250$ 7
H Highest numbers of hubs for the hub detection on the discovery data, compared with the results on validation data, $n = 500$ 8
I Highest numbers of hubs for the hub detection on the discovery data, compared with the results on validation data, $n = 1000$ 8
J Highest numbers of hubs for the hub detection on the discovery data, compared with the results on validation data, $n = 4000$ 9

Fig A-E display the results of the hub detection applied to the discovery data. For each method combination on the x-axis, the 50 results obtained from 50 different discovery datasets are summarized as boxplots, indicating the number of detected hubs. Outliers are marked by black crosses. Results that were picked as the "best result" in one of the 50 samplings are marked by red squares.

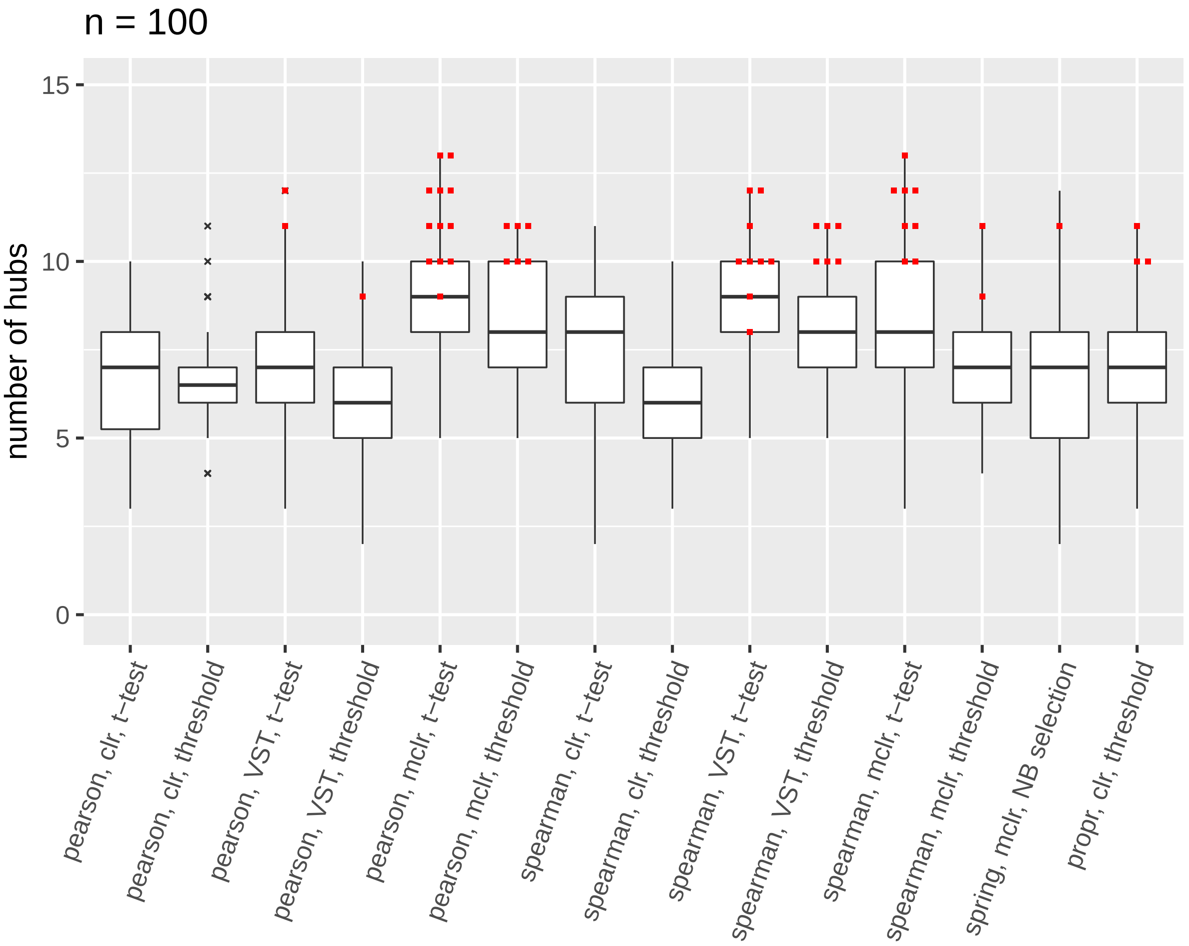

Fig A. Results for hub detection on the discovery data, $n = 100$

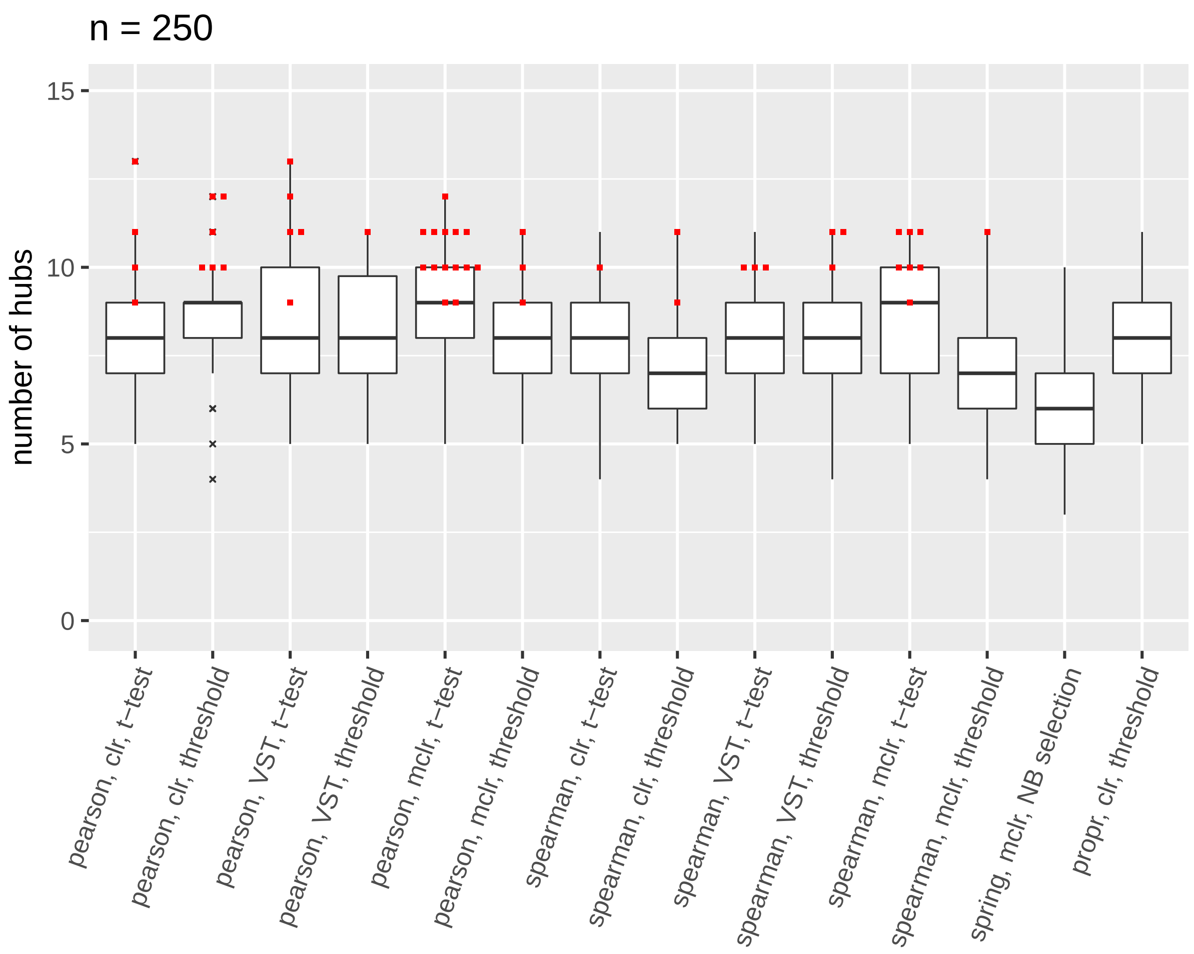

Fig B. Results for hub detection on the discovery data, $n = 250$

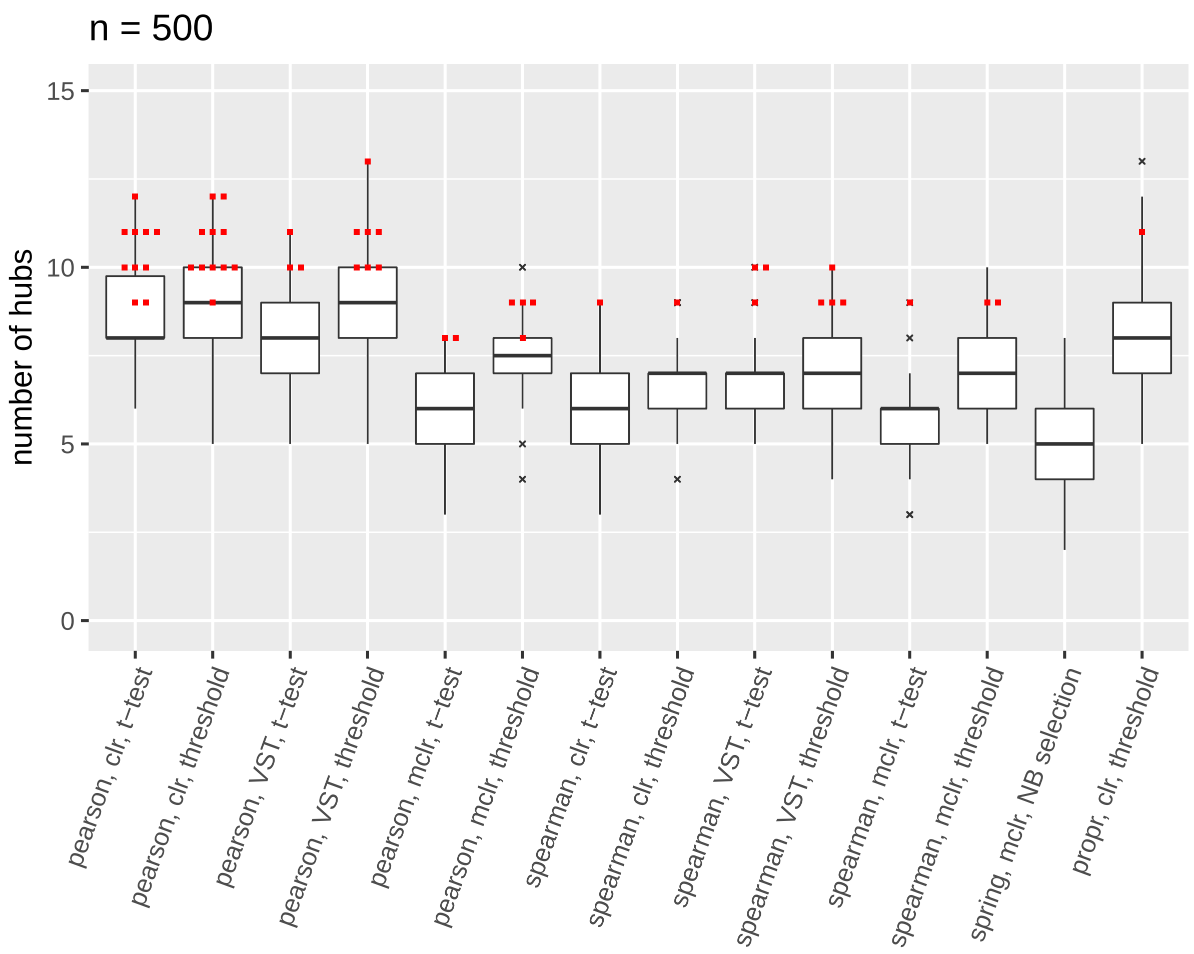

Fig C. Results for hub detection on the discovery data, $n = 500$

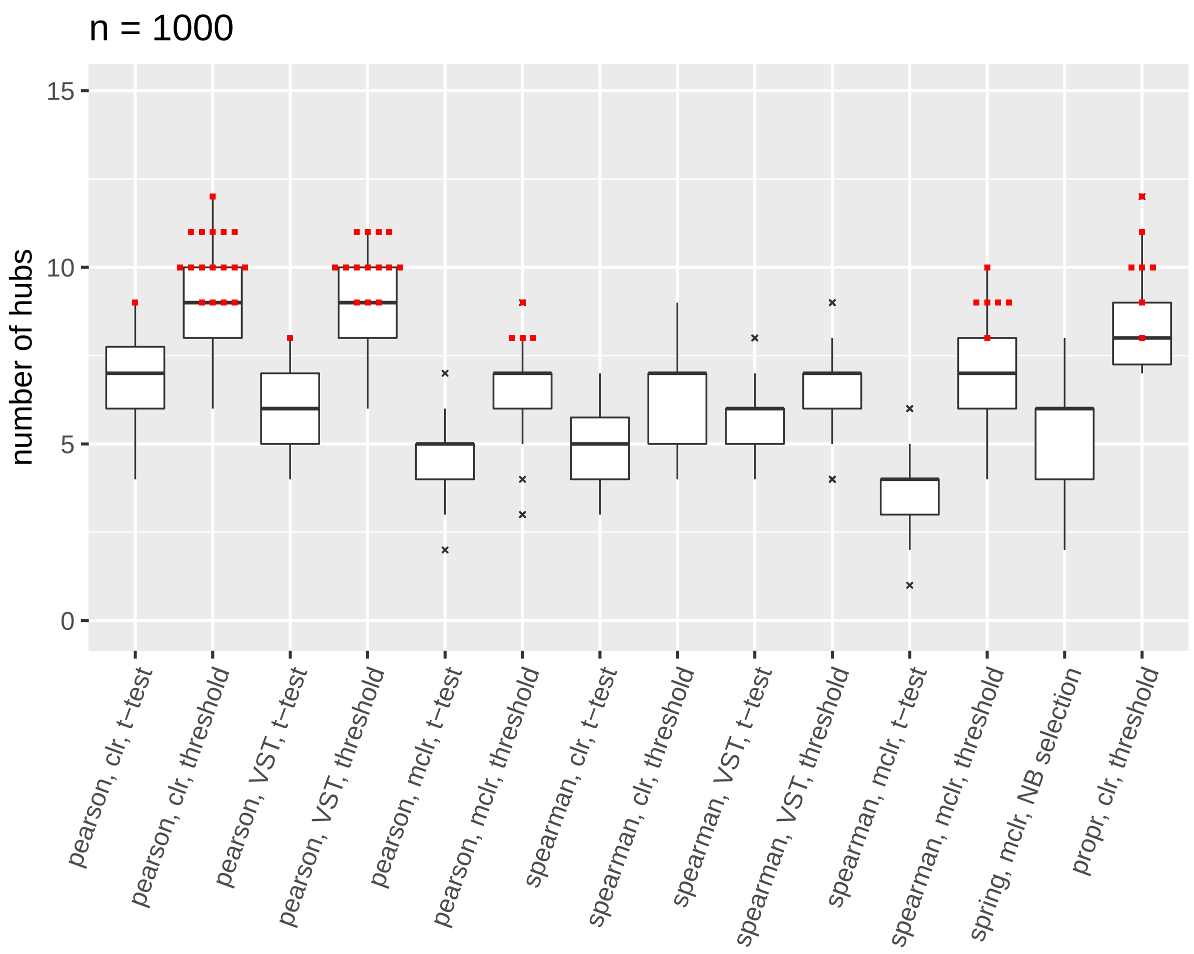

Fig D. Results for hub detection on the discovery data, $n = 1000$

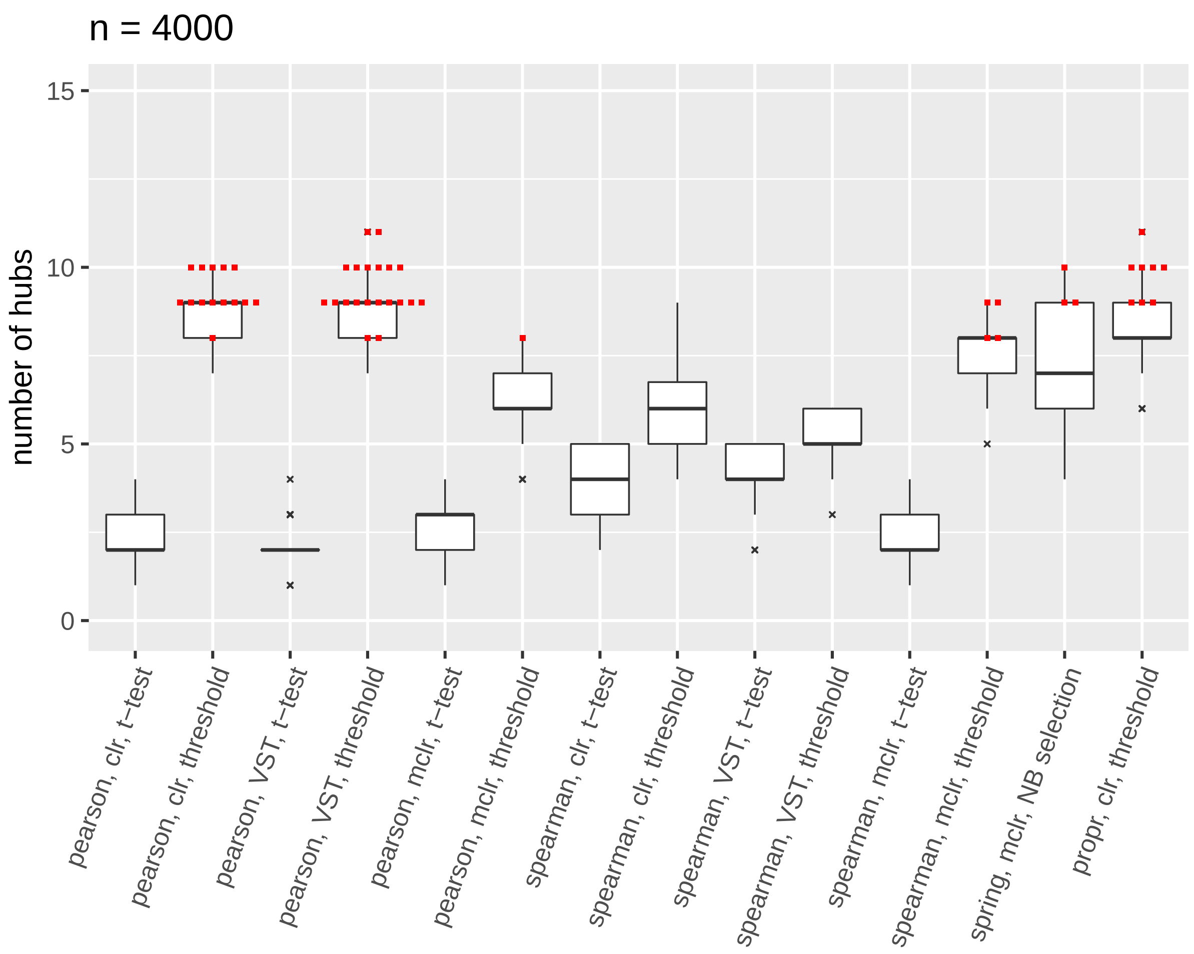

Fig E. Results for hub detection on the discovery data, $n = 4000$

There is not one single method combination that always yields the highest number of hubs. At $n = 100$, the best results are often found by Pearson correlation with mclr normalization, and Spearman correlation with VST or mclr normalization. With increasing sample size, Pearson correlation with clr or VST normalization frequently yields high number of hubs. As Fig D and Fig E show, for $n = 1000$ and $n = 4000$, sparsification of the network with the t-test generally leads to lower number of hubs compared to sparsification with the threshold method. At these sample sizes, the threshold method has a stronger sparsification effect than the t-test (given the chosen threshold of 0.15) and sparser networks tend to have more hubs for the chosen hub definition.

We consider the results of applying the chosen method combinations to the validation data. For each method combination that was chosen at least once as the "best" one, Fig F-J display the number of hubs obtained by the method on the discovery data vs. the number obtained by the same method on the validation data, where each square-dot combination corresponds to one of the 50 samplings. The results on discovery and validation data are connected by lines.

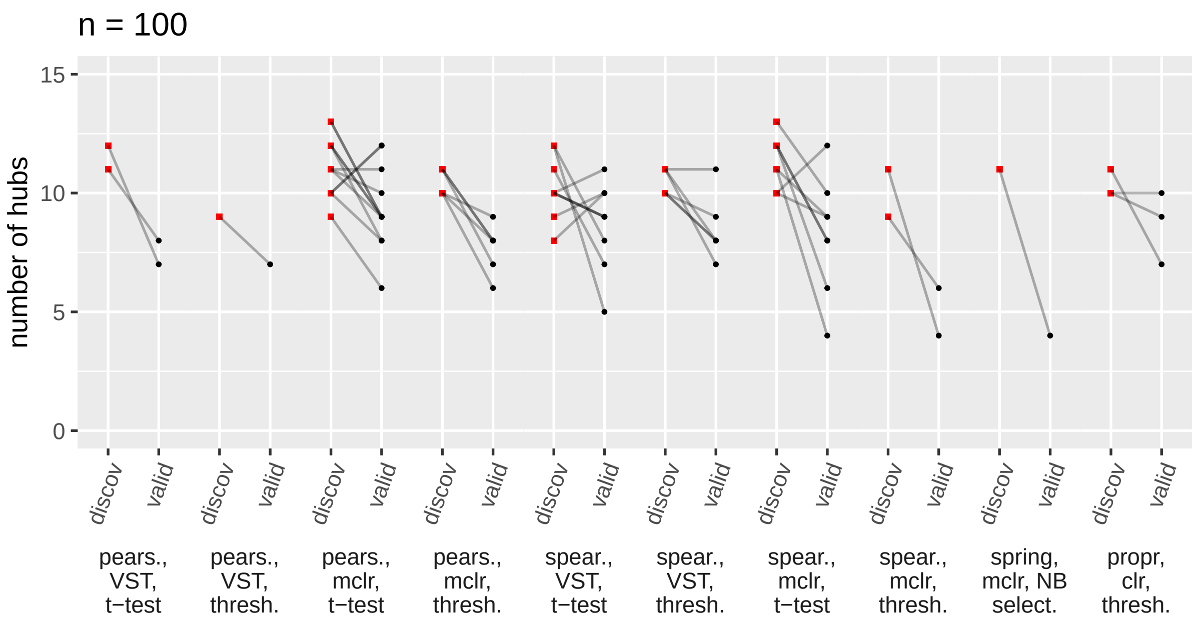

Fig F. Highest numbers of hubs for the hub detection on the discovery data, compared with the results on validation data, $n = 100$

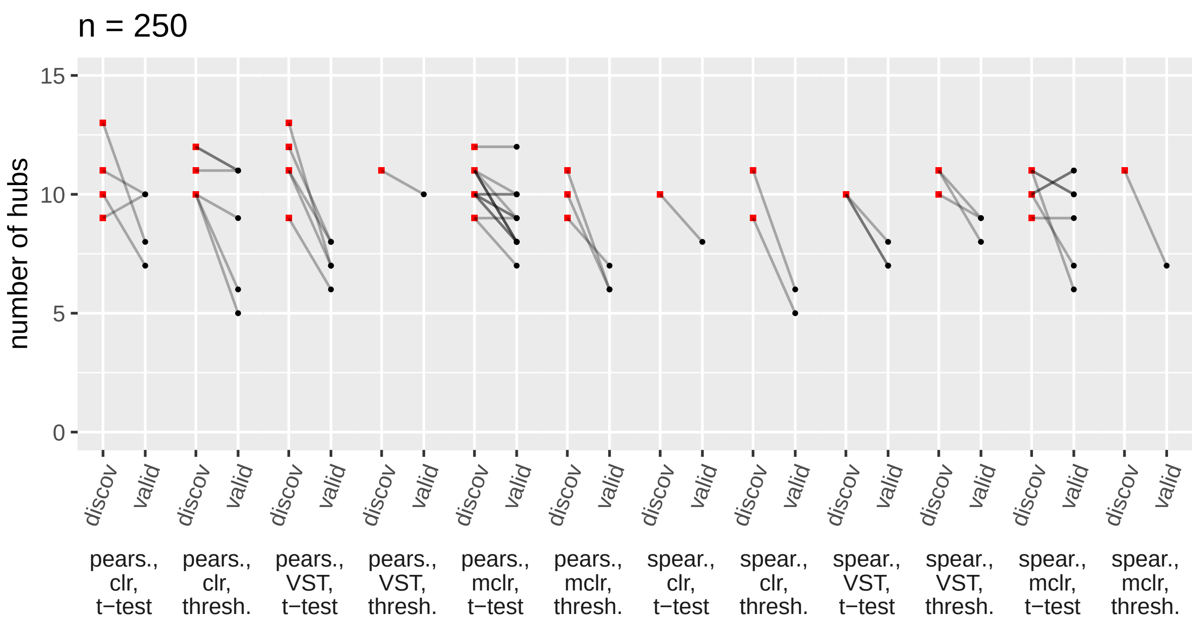

Fig G. Highest numbers of hubs for the hub detection on the discovery data, compared with the results on validation data, $n = 250$

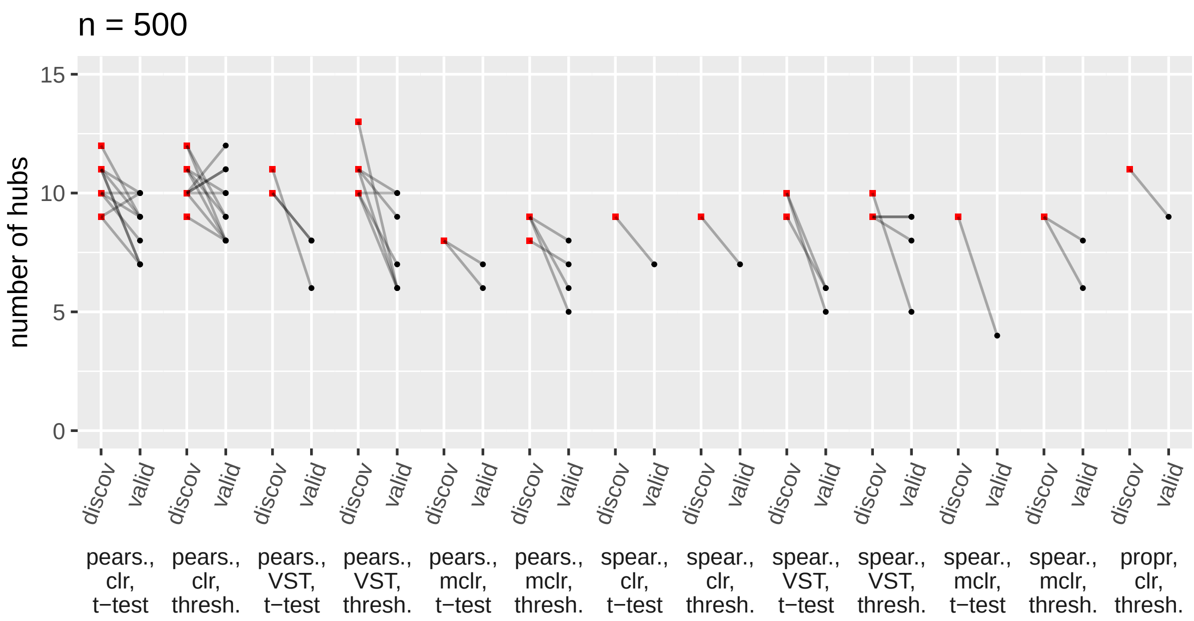

Fig H. Highest numbers of hubs for the hub detection on the discovery data, compared with the results on validation data, $n = 500$

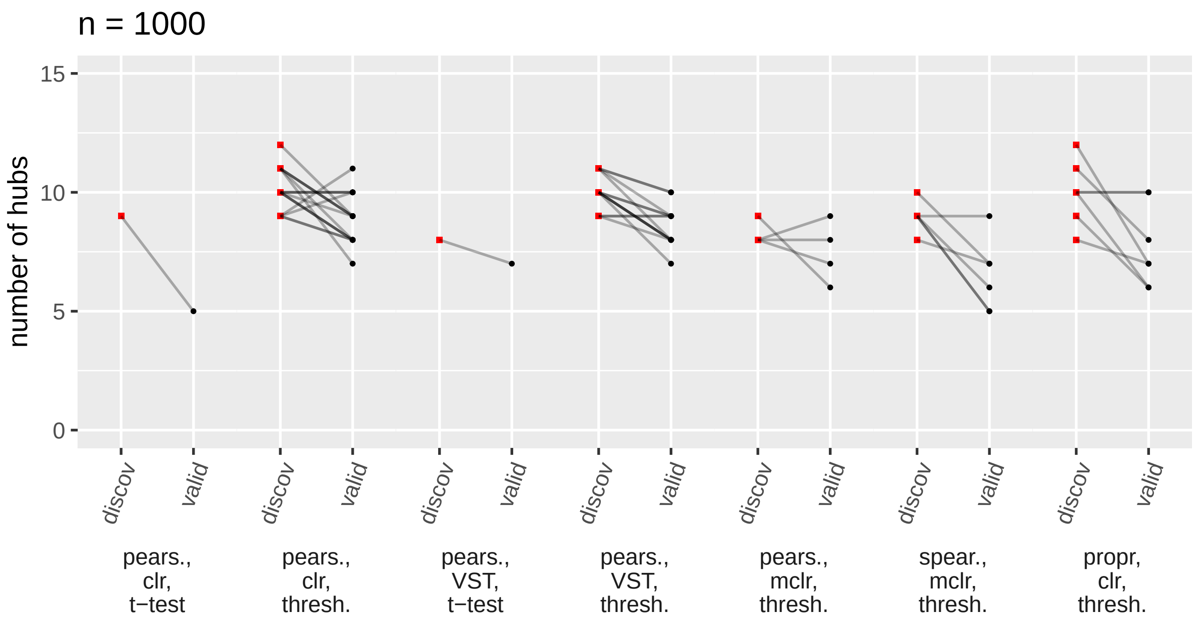

Fig I. Highest numbers of hubs for the hub detection on the discovery data, compared with the results on validation data, $n = 1000$

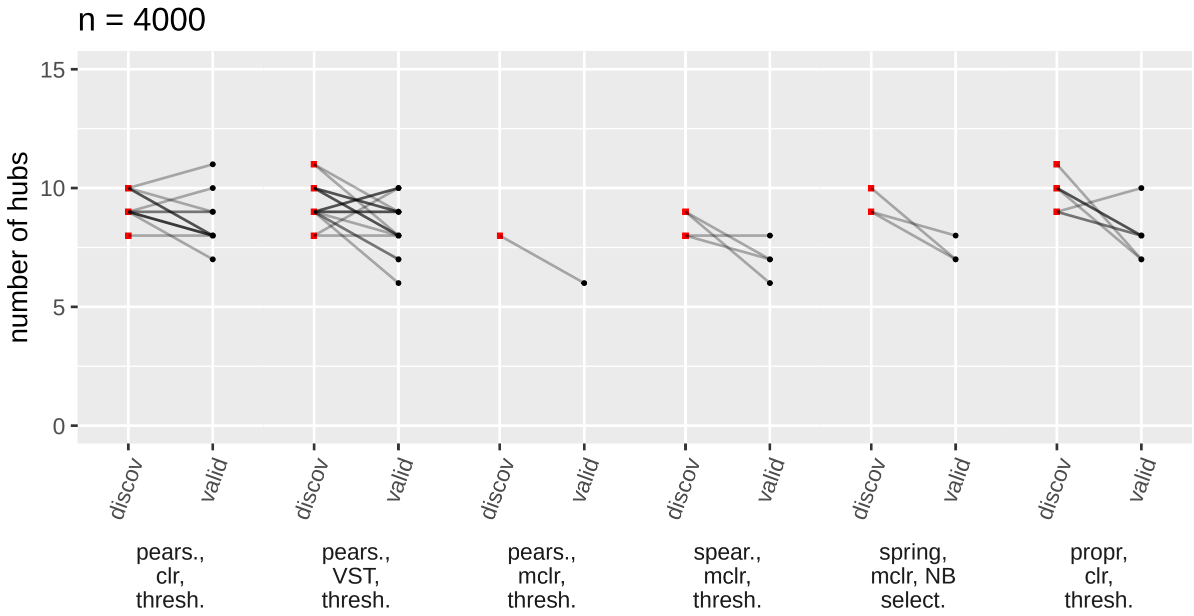

Fig J. Highest numbers of hubs for the hub detection on the discovery data, compared with the results on validation data, $n = 4000$

The lines point downwards in the majority of the 50 samplings, indicating worse results regarding the network's hubbiness on the validation data.
