# Supplementary material for: Over-optimism in unsupervised microbiome analysis: Insights from network learning and clustering
Source: PLoS Comput Biol. 2023 Jan 6;19(1):e1010820. doi: 10.1371/journal.pcbi.1010820 (PMC9873197; doi:10.1371/journal.pcbi.1010820)
Supplement: S3 Text — (PDF) [file pcbi.1010820.s003.pdf]

S3: Full results and plots for research task 3 (differential network analysis)

List of Figures

A Results for differential network analysis on the discovery data, $n = 100$ . . 2
B Results for differential network analysis on the discovery data, $n = 250$ . . 3
C Results for differential network analysis on the discovery data, $n = 500$ . . 4
D Largest GCDs for the differential network analysis on the discovery data, compared with the results on validation data, $n = 100$ 5
E Largest GCDs for the differential network analysis on the discovery data, compared with the results on validation data, $n = 250$ 5
F Largest GCDs for the differential network analysis on the discovery data, compared with the results on validation data, $n = 500$ 6

Fig A-C show the results of the differential network analysis on the discovery data over 50 samplings. Boxplots summarize the GCDs between the microbial network based on the non-antibiotics samples vs. the network based on the antibiotics samples. GCDs that were picked as the "best" results are marked by red squares.

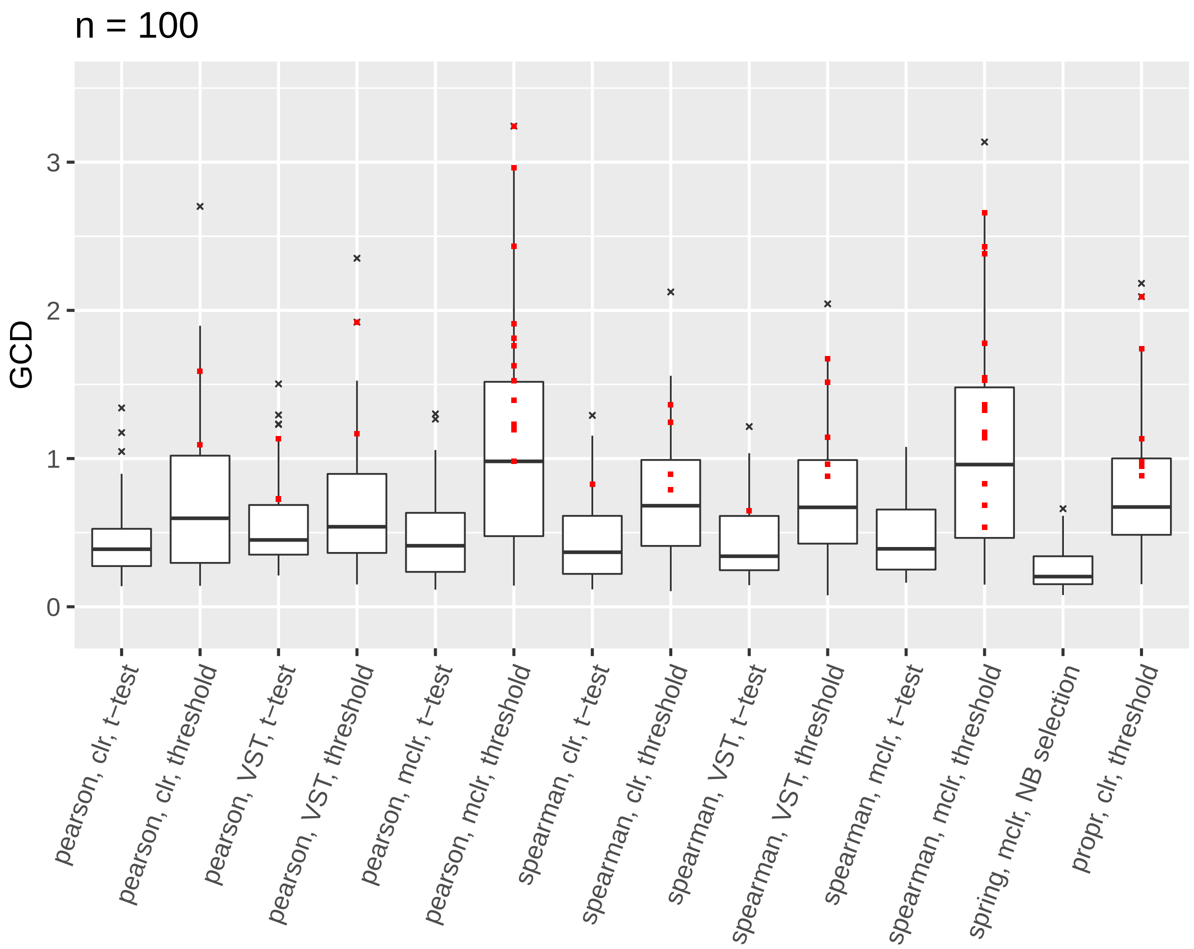

Fig A. Results for differential network analysis on the discovery data, $n = 100$

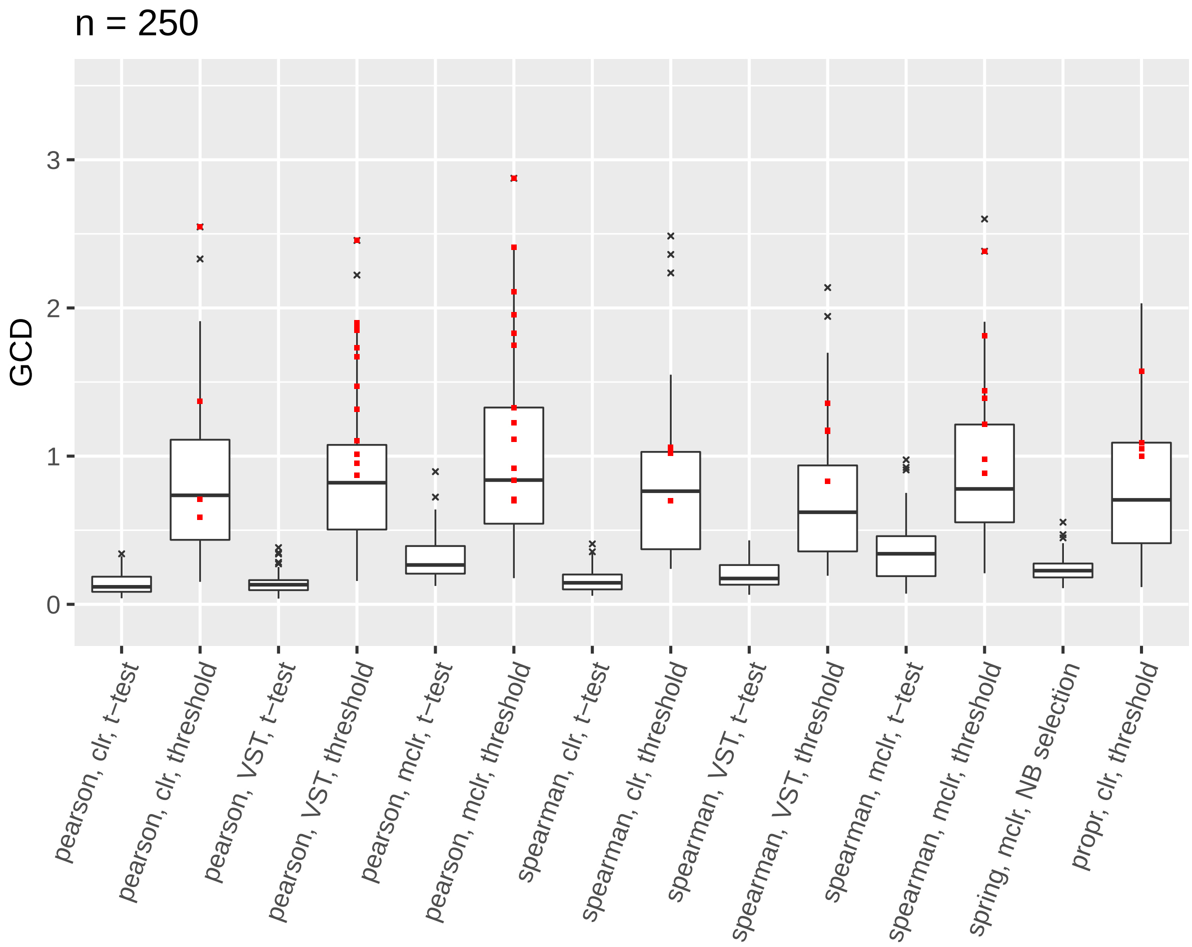

Fig B. Results for differential network analysis on the discovery data, $n = 250$

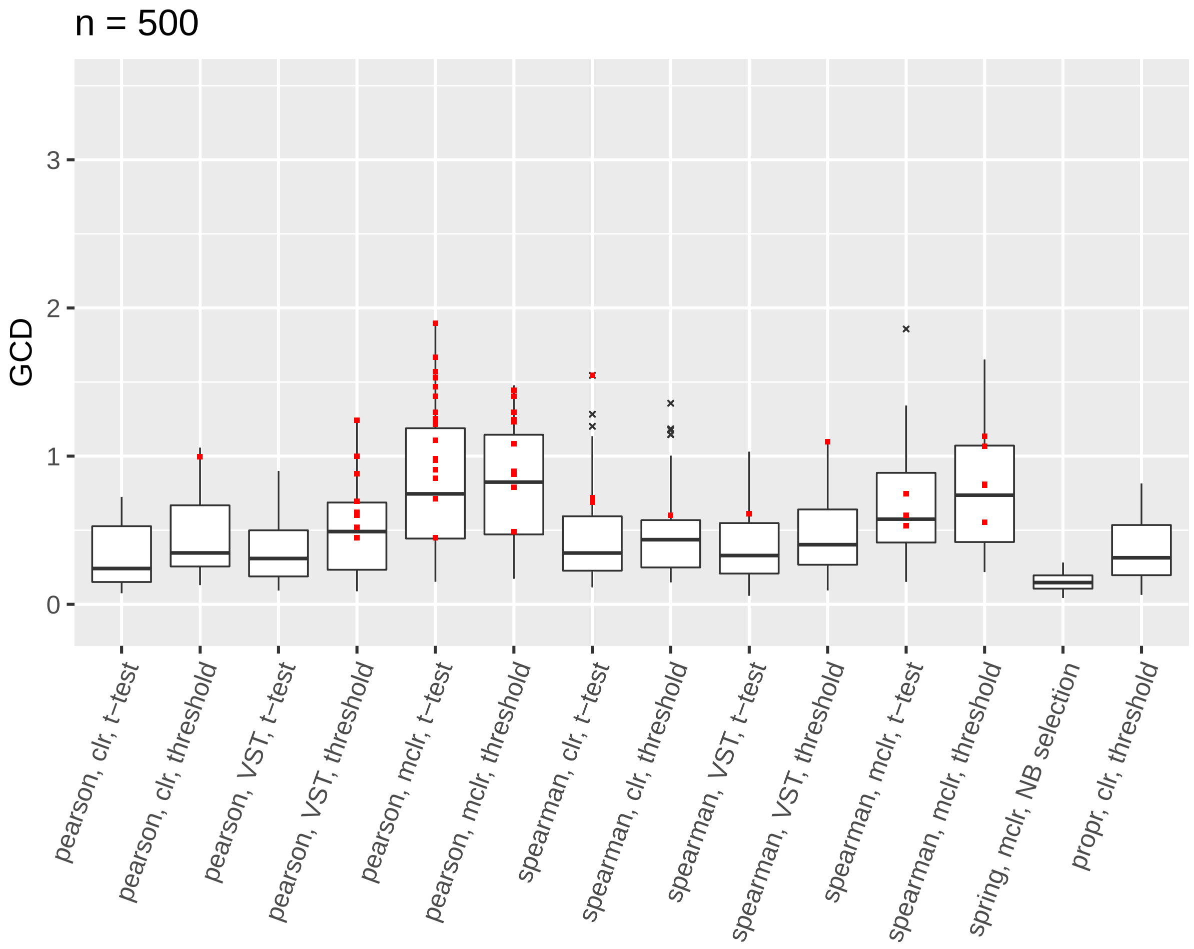

Fig C. Results for differential network analysis on the discovery data, $n = 500$

Similar to hub detection, there is no superior method combination that always leads to best results, i.e., highest GCD values. Notably, sparsification via t-test never leads to best results for $n = 100$ and $n = 250$, but only for $n = 500$. However, a general trend cannot be confirmed due to the limited sample size in this research task.

Fig D-F show the results of applying the best method combinations to the validation data and compare these to the results on the discovery data. Over-optimism is indicated by downward lines, which is the case in about 75% of the 50 samplings.

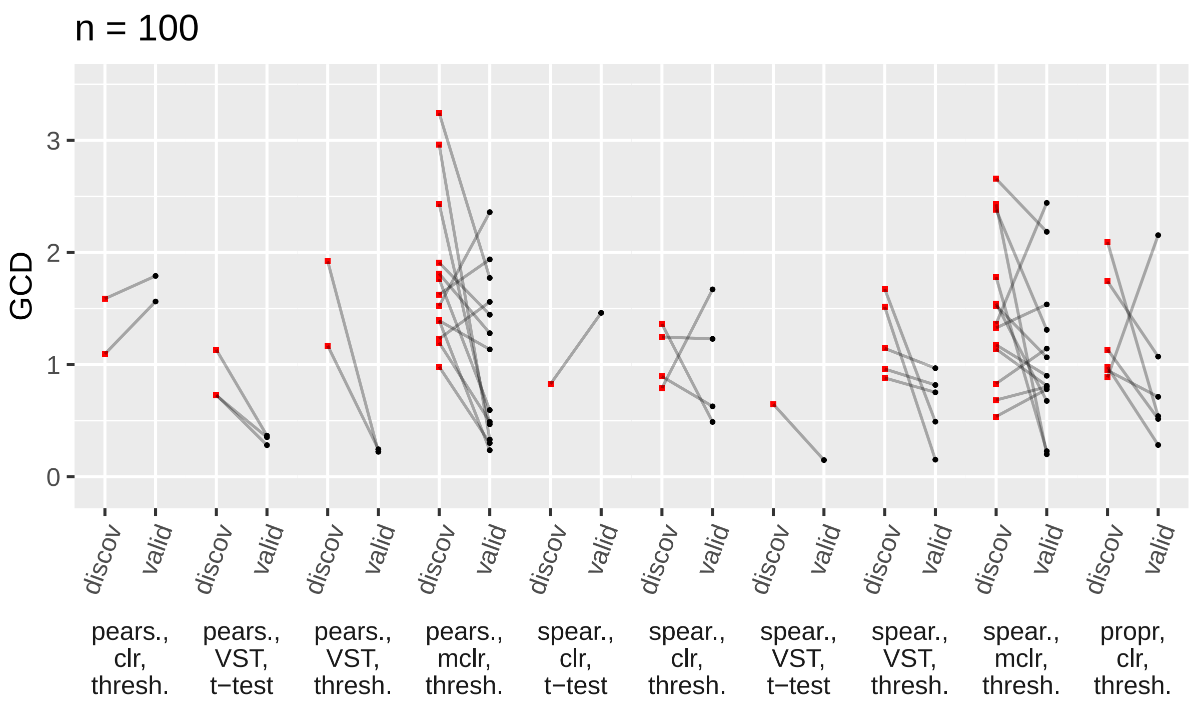

Fig D. Largest GCDs for the differential network analysis on the discovery data, compared with the results on validation data, $n = 100$

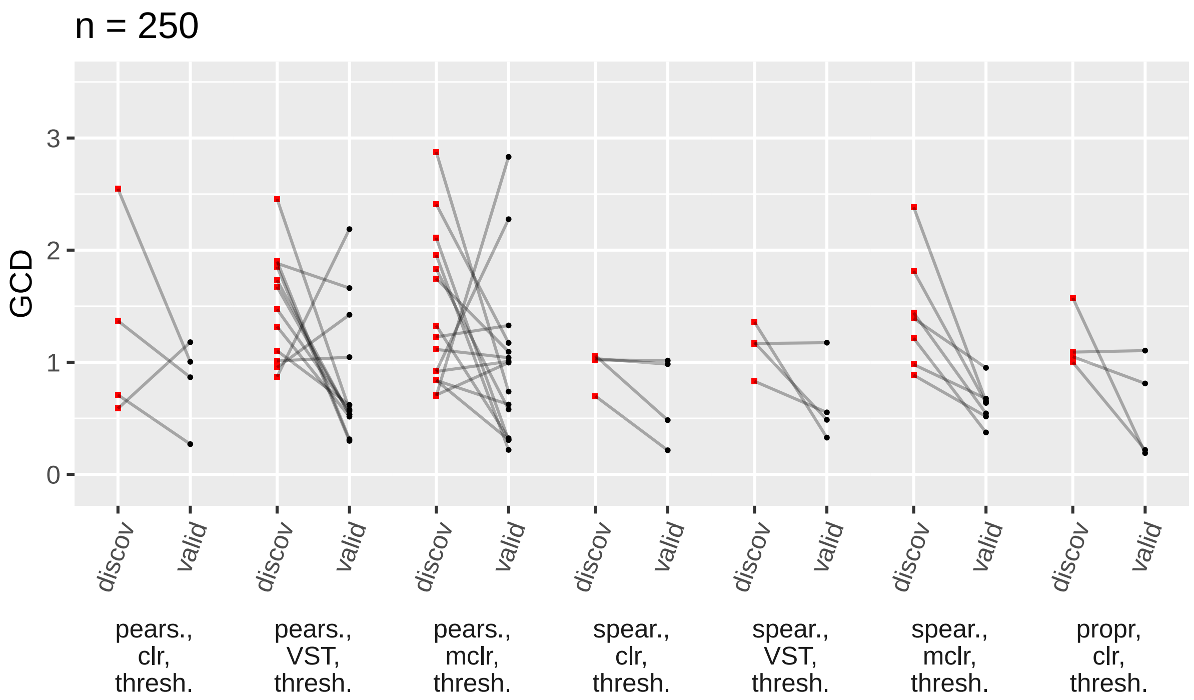

Fig E. Largest GCDs for the differential network analysis on the discovery data, compared with the results on validation data, $n = 250$

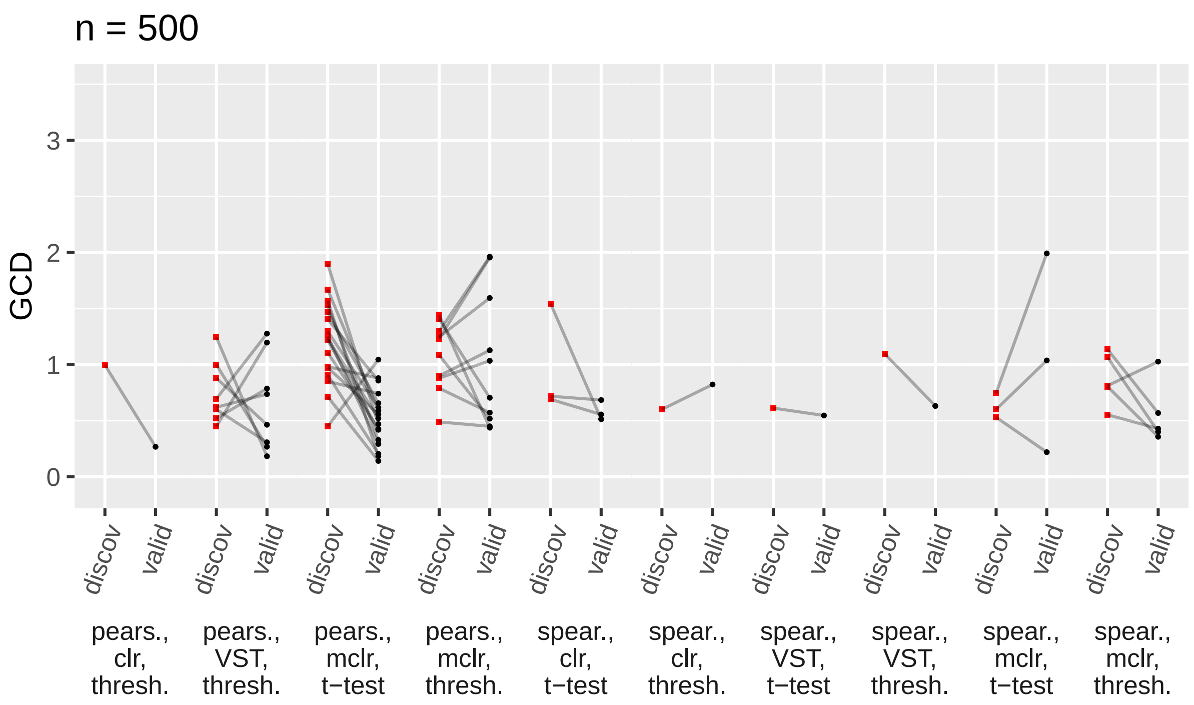

Fig F. Largest GCDs for the differential network analysis on the discovery data, compared with the results on validation data, $n = 500$
