# Supplementary material for: Over-optimism in unsupervised microbiome analysis: Insights from network learning and clustering
Source: PLoS Comput Biol. 2023 Jan 6;19(1):e1010820. doi: 10.1371/journal.pcbi.1010820 (PMC9873197; doi:10.1371/journal.pcbi.1010820)
Supplement: S4 Text — (PDF) [file pcbi.1010820.s004.pdf]

S4: Full results and plots for research task 4 (clustering of samples)

List of Figures

A Results for clustering samples on the discovery data, $n = 100$ 2
B Results for clustering samples on the discovery data, $n = 250$ 3
C Results for clustering samples on the discovery data, $n = 500$ 3
D Results for clustering samples on the discovery data, $n = 1000$ 4
E Results for clustering samples on the discovery data, $n = 3500$ 4
F Results for network-based clustering of samples on the discovery data, separated by sparsification methods, $n = 100$ 6
G Results for network-based clustering of samples on the discovery data, separated by sparsification methods, $n = 250$ 6
H Results for network-based clustering of samples on the discovery data, separated by sparsification methods, $n = 500$ 7
I Results for network-based clustering of samples on the discovery data, separated by sparsification methods, $n = 1000$ 7
J Results for network-based clustering of samples on the discovery data, separated by sparsification methods, $n = 3500$ 8
K Best ASWs for the clustering of samples on the discovery data, compared with the results on validation data, $n = 100$ 9
L Best ASWs for the clustering of samples on the discovery data, compared with the results on validation data, $n = 250$ 9
M Best ASWs for the clustering of samples on the discovery data, compared with the results on validation data, $n = 500$ 10
N Best ASWs for the clustering of samples on the discovery data, compared with the results on validation data, $n = 1000$ 10
O Best ASWs for the clustering of samples on the discovery data, compared with the results on validation data, $n = 3500$ 11

Fig A-E display the results of the sample clustering on the discovery data over 50 samplings. The ASW results are summarized by boxplots and are additionally shown as colored dots, with the color indicating the number k of clusters in the respective clustering result. Results picked as the "best result" in one of the 50 samplings are marked by red square edges. For the network-based clustering methods (fast greedy modularity optimization and the Louvain method), the results for threshold and K-nearest neighbor sparsification are displayed together, i.e., $50^*2 = 100$ results are shown for these method combinations.

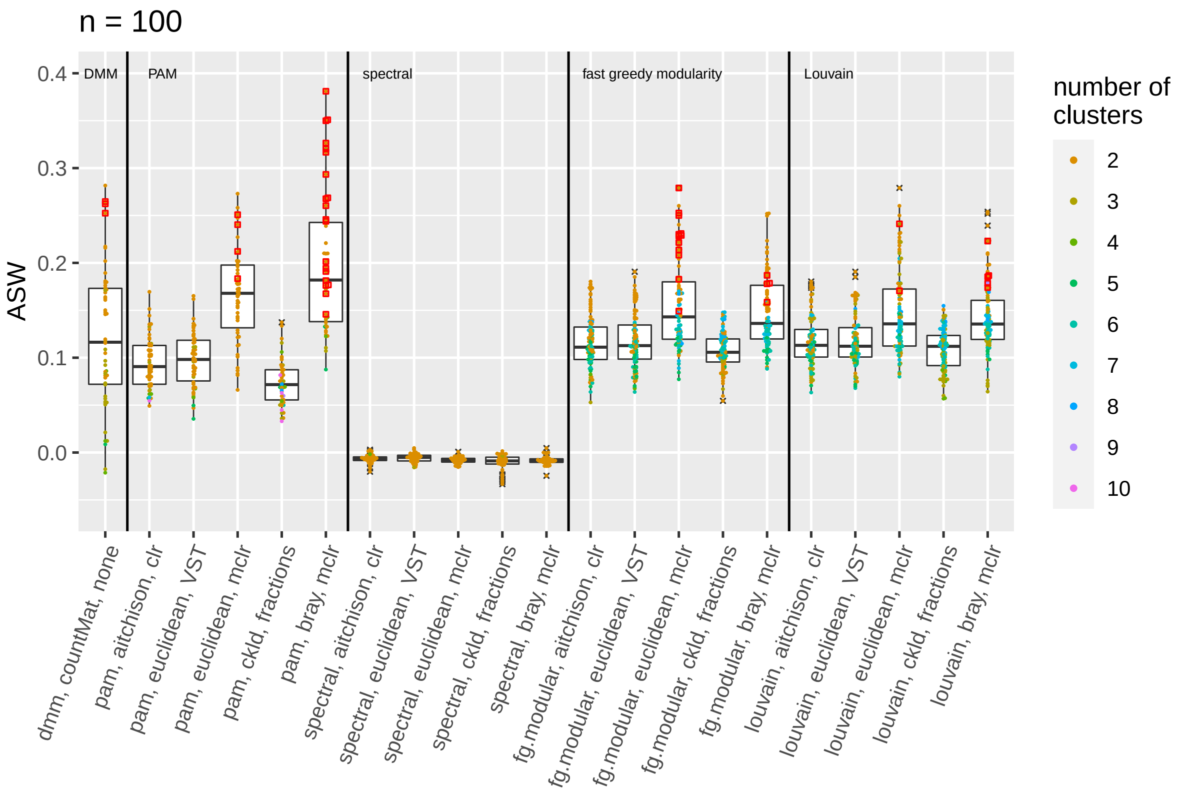

Fig A. Results for clustering samples on the discovery data, $n = 100$

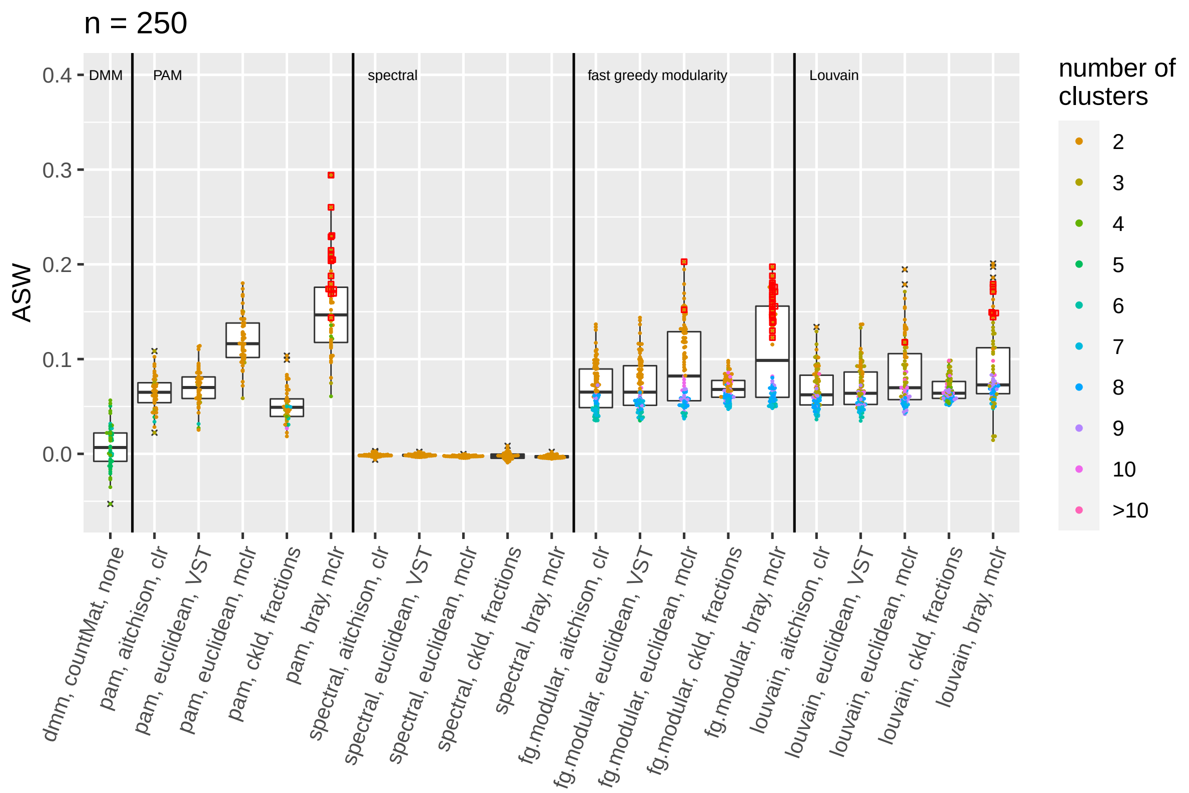

Fig B. Results for clustering samples on the discovery data, $n = 250$

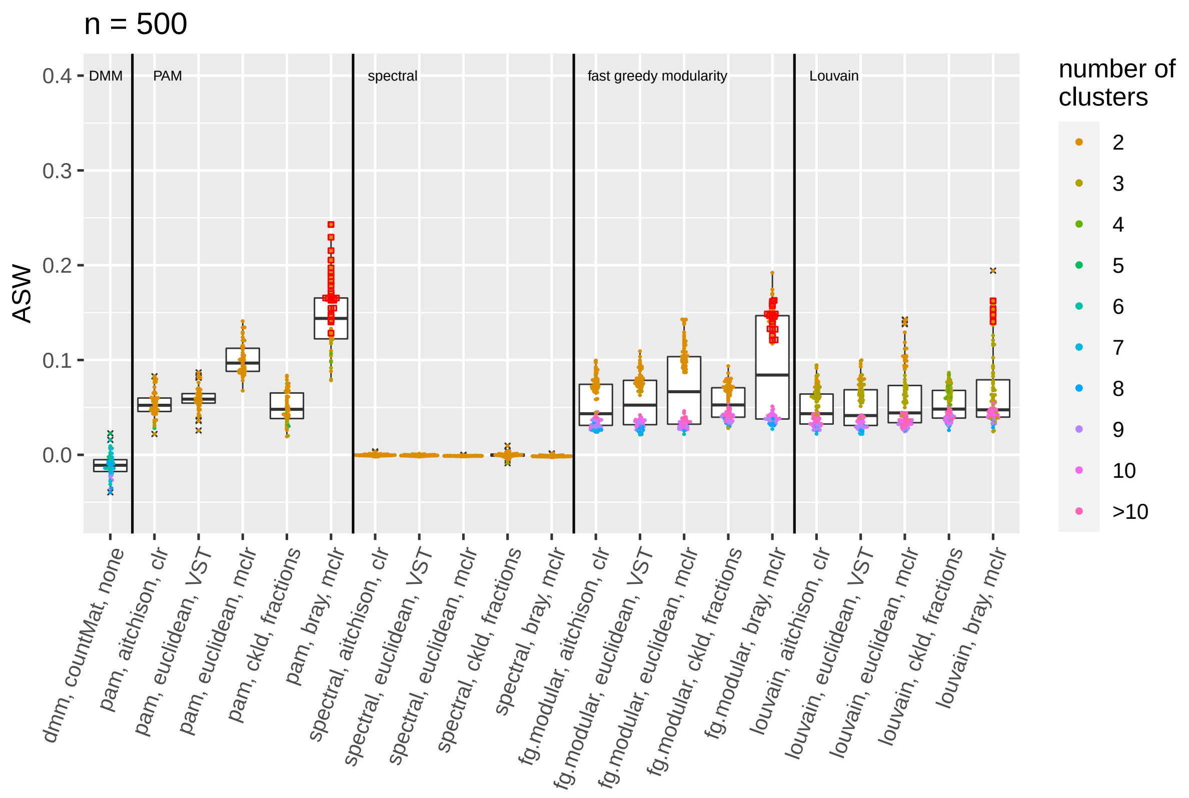

Fig C. Results for clustering samples on the discovery data, $n = 500$

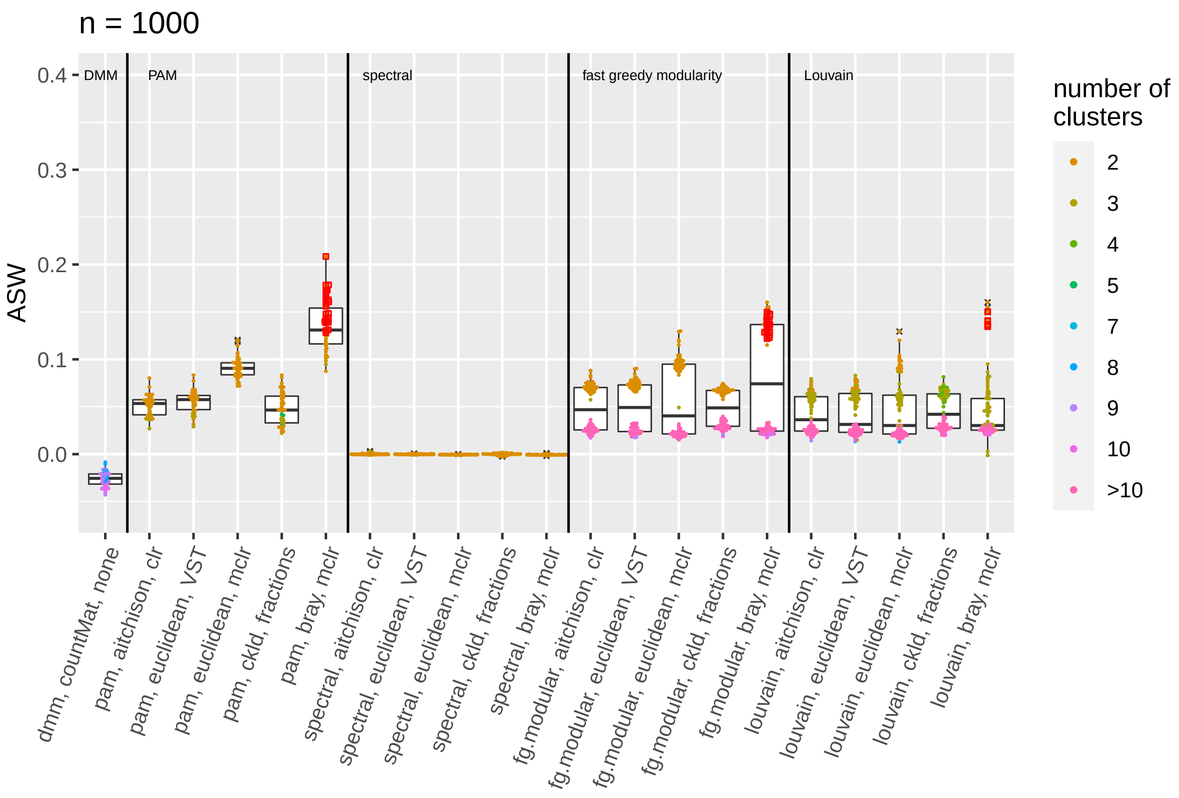

Fig D. Results for clustering samples on the discovery data, $n = 1000$

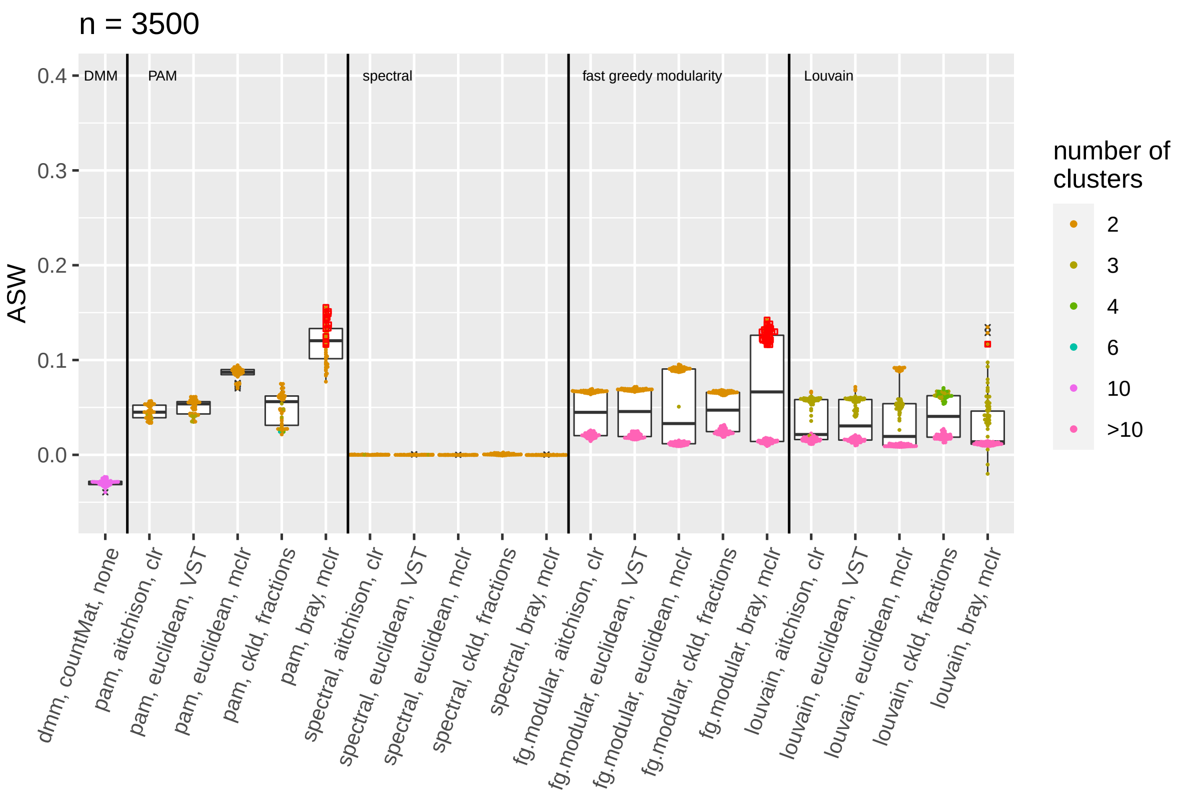

Fig E. Results for clustering samples on the discovery data, $n = 3500$

Overall, the ASW values are not particularly large, indicating at best a moderate quality of the clustering. Such results are not uncommon in enterotype research; for example, the original study about enterotypes [1] reported ASW values that were less than or equal to 0.25. From a sample size of $n = 250$ upwards, the number of clusters is mostly chosen as two or three, which fits with previous results from studies about enterotypes [1, 2, 3, 4]. Similar to the other three research tasks, there is not a single method combination that always yields the best results. PAM, fast greedy modularity optimization and the Louvain method are frequently chosen as the best clustering methods, often in combination with the Bray-Curtis dissimilarity and mclr normalization. DMM clustering performs reasonably well for $n = 100$, but does not yield good ASW values for the other sample sizes. Spectral clustering yields ASW values around zero for all sample sizes.
Fig F-J depict the results for the network-based clustering (fast greedy modularity optimization and the Louvain method) separately for both sparsification methods (threshold and K-nearest neighbors). Results that were picked as the "best result" in one of the 50 samplings are marked by red square edges. As the figures show, sparsification with the threshold method leads to smaller numbers of clusters and to larger ASW values. The threshold method has a weaker sparsification effect than the K-nearest neighbor method (given the chosen threshold of 0.85 and the number of nearest neighbors set to $K = 3$), and the cluster algorithms tend to find fewer clusters in denser (less sparse) networks. Similar to the previous research tasks, this demonstrates that network sparsification can have a notable effect on the final results.

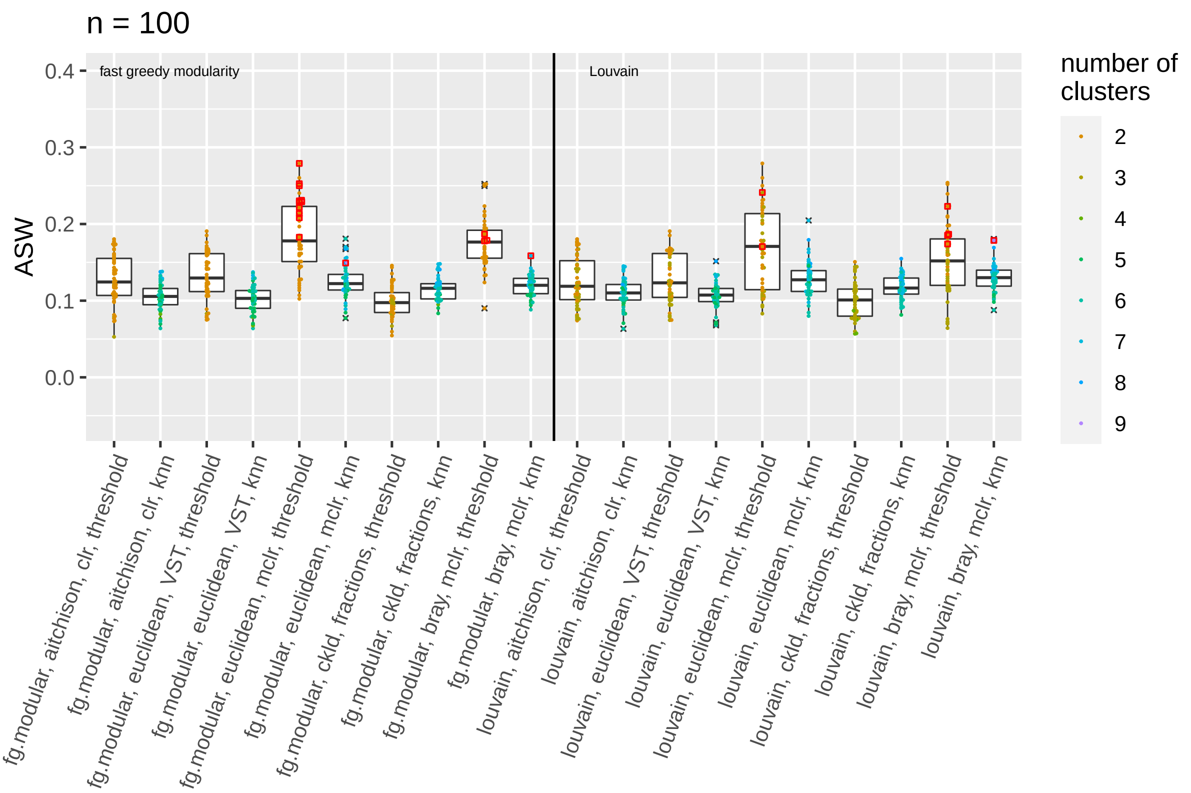

Fig F. Results for network-based clustering of samples on the discovery data, separated by sparsification methods, $n = 100$

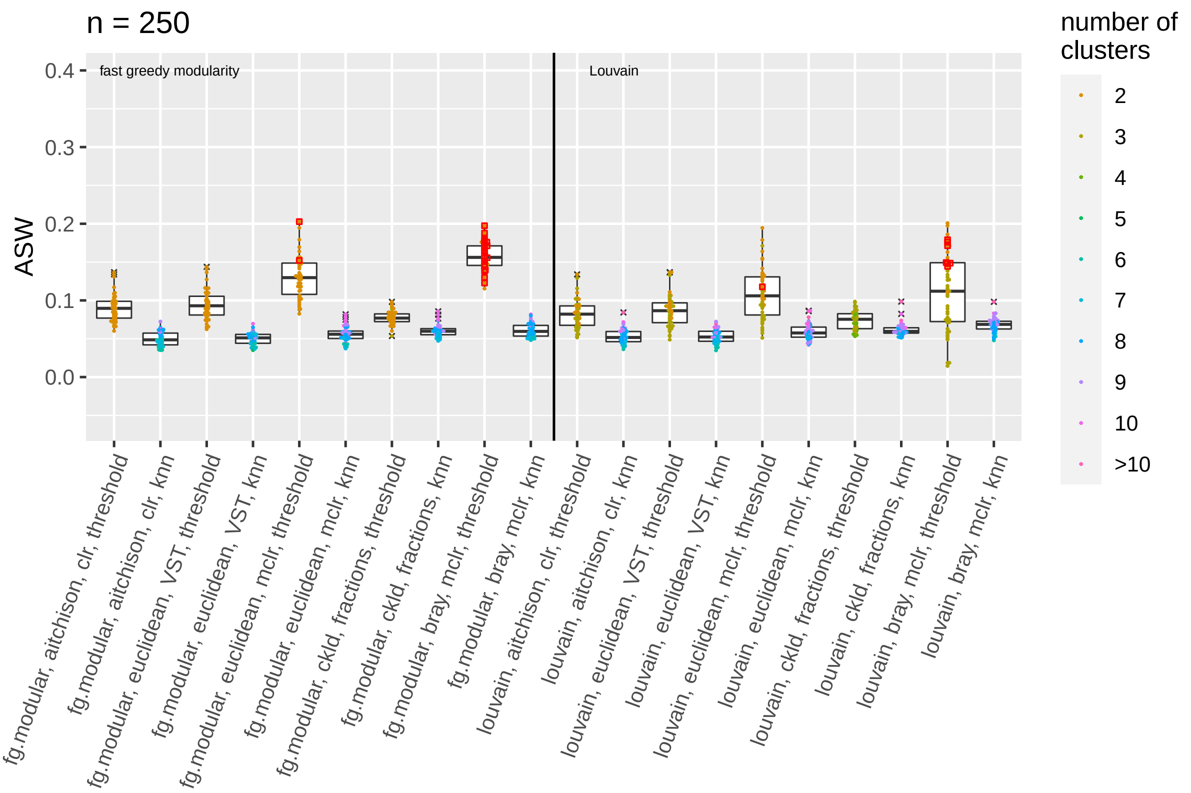

Fig G. Results for network-based clustering of samples on the discovery data, separated by sparsification methods, $n = 250$

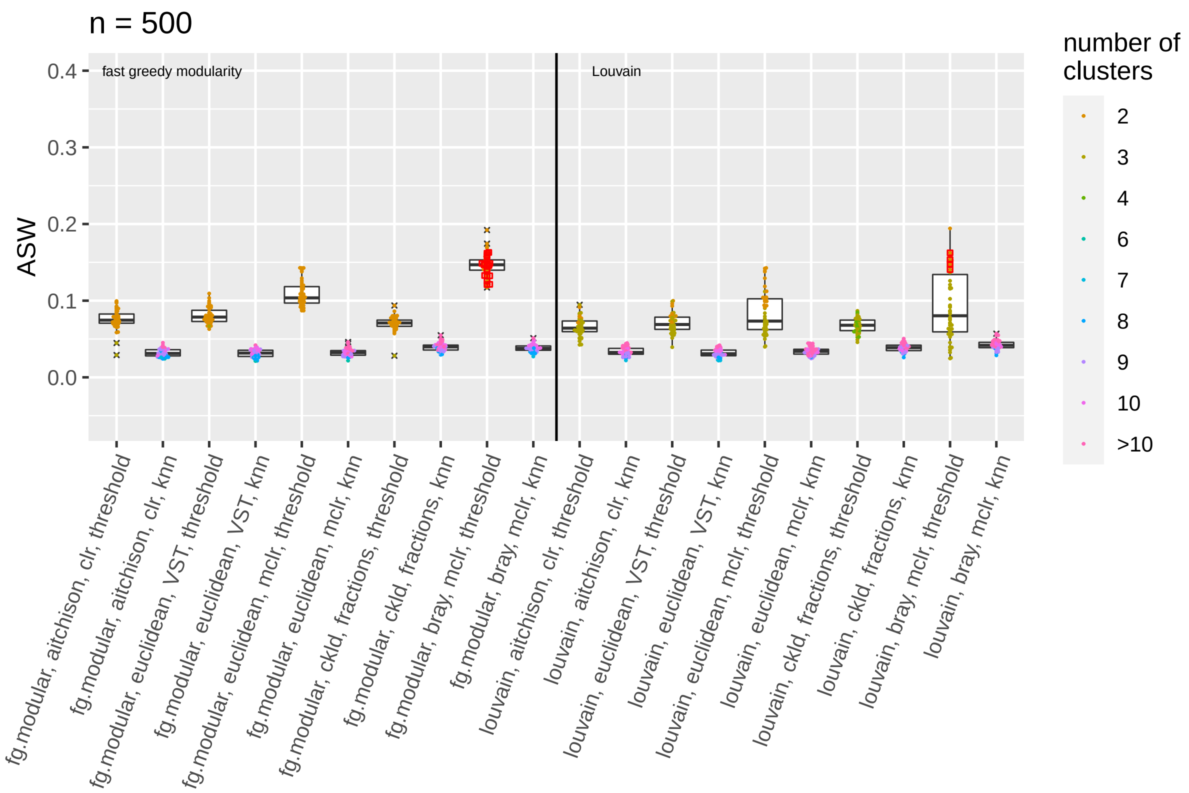

Fig H. Results for network-based clustering of samples on the discovery data, separated by sparsification methods, $n = 500$

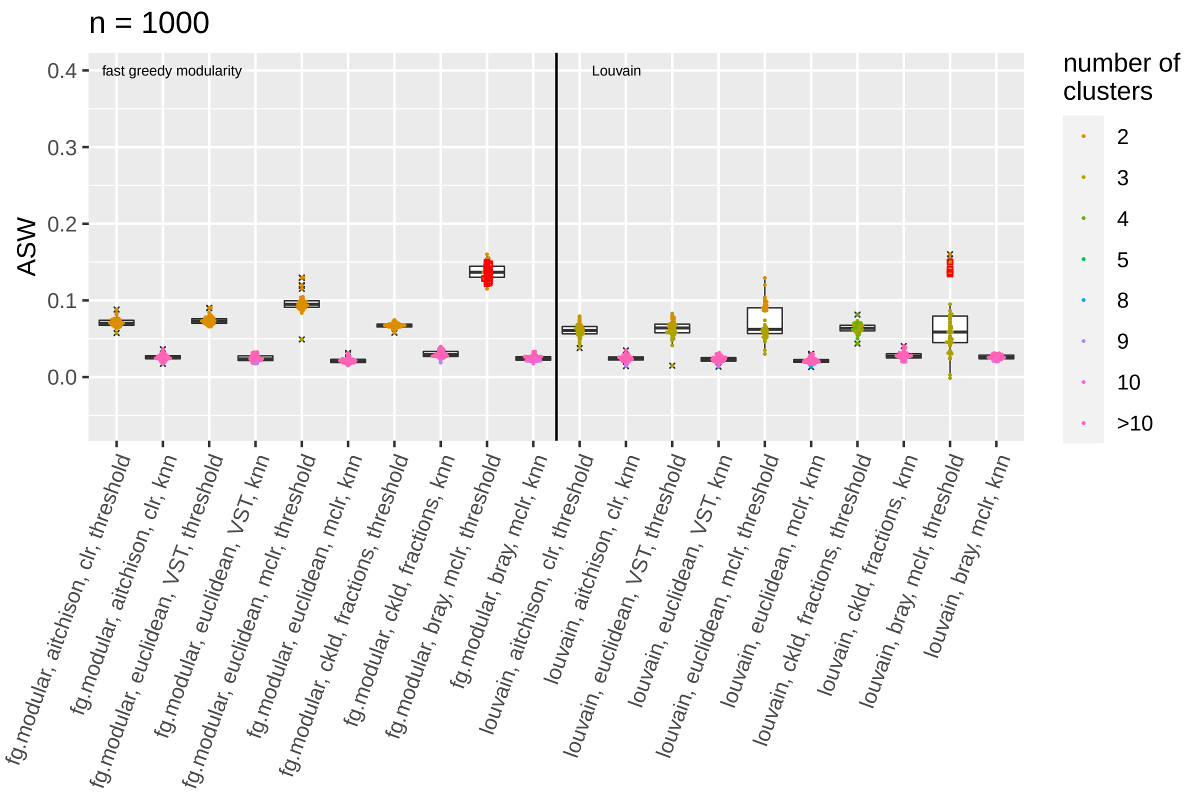

Fig I. Results for network-based clustering of samples on the discovery data, separated by sparsification methods, $n = 1000$

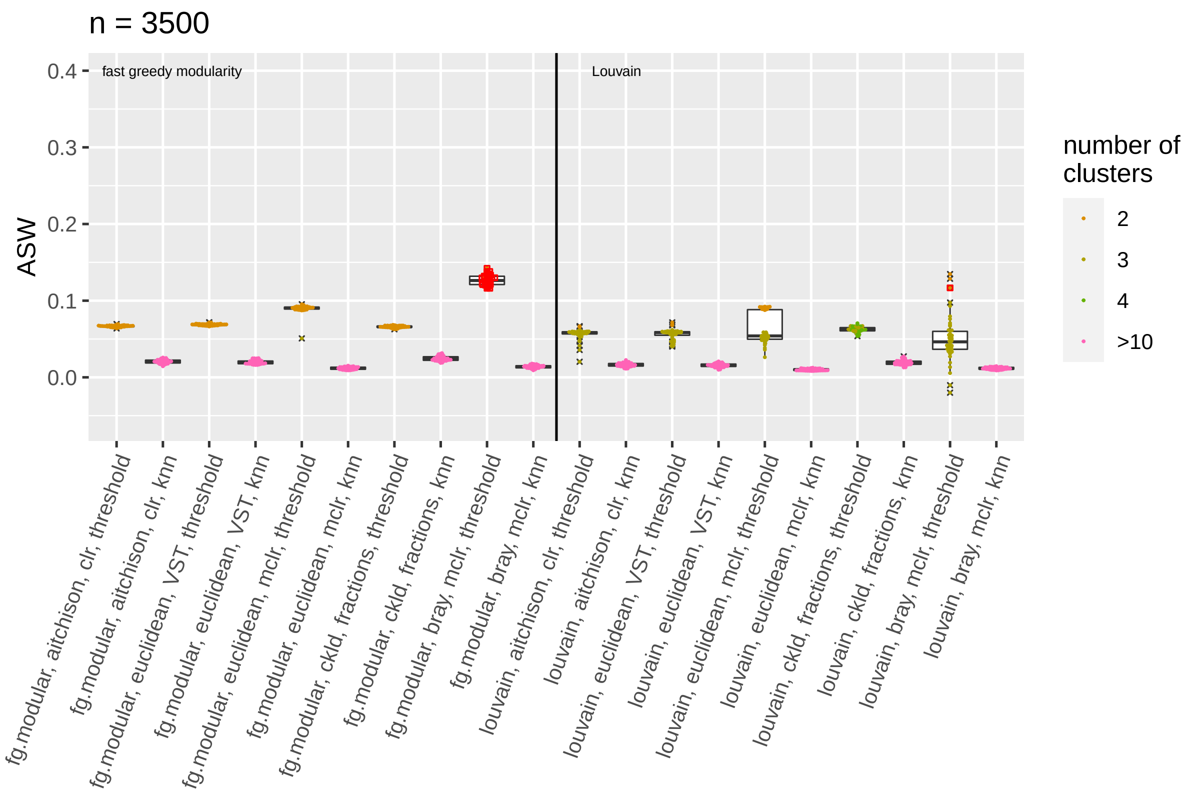

Fig J. Results for network-based clustering of samples on the discovery data, separated by sparsification methods, $n = 3500$

Fig K-O compare the ASW values resulting from the best method combinations on the discovery data to the corresponding ASW values on the validation data. Lines that point downwards indicate over-optimistic bias. For $n = 100$ and $n = 250$, this is the case in about 75% of the 50 samplings, for $n = 500$ and $n = 3500$, in about 67% of the samplings, and for $n = 1000$, in 54% of the samplings.

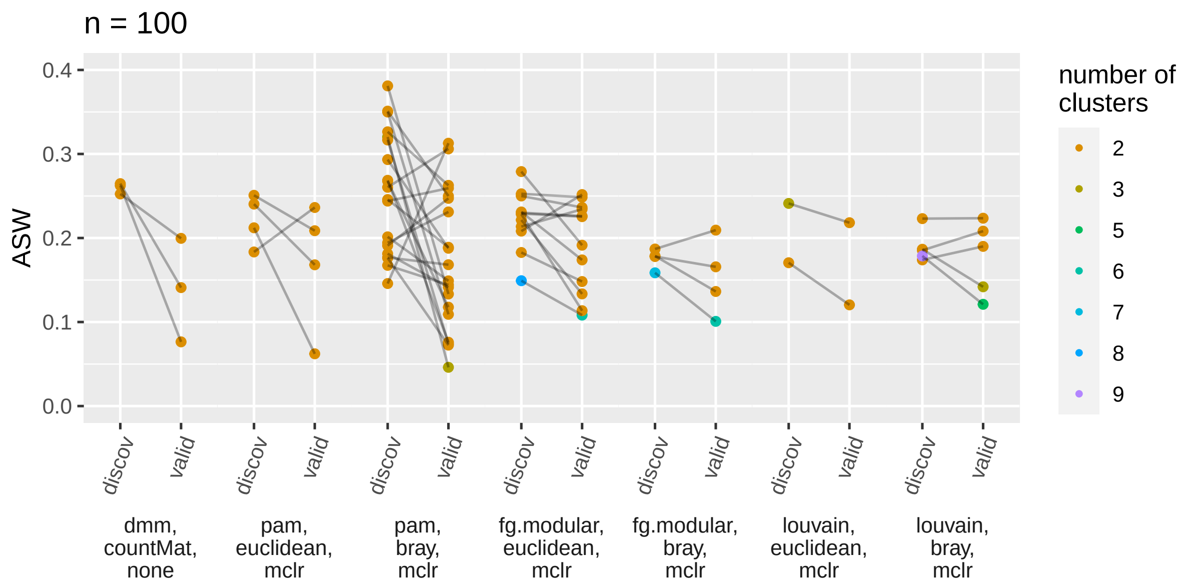

Fig K. Best ASWs for the clustering of samples on the discovery data, compared with the results on validation data, $n = 100$

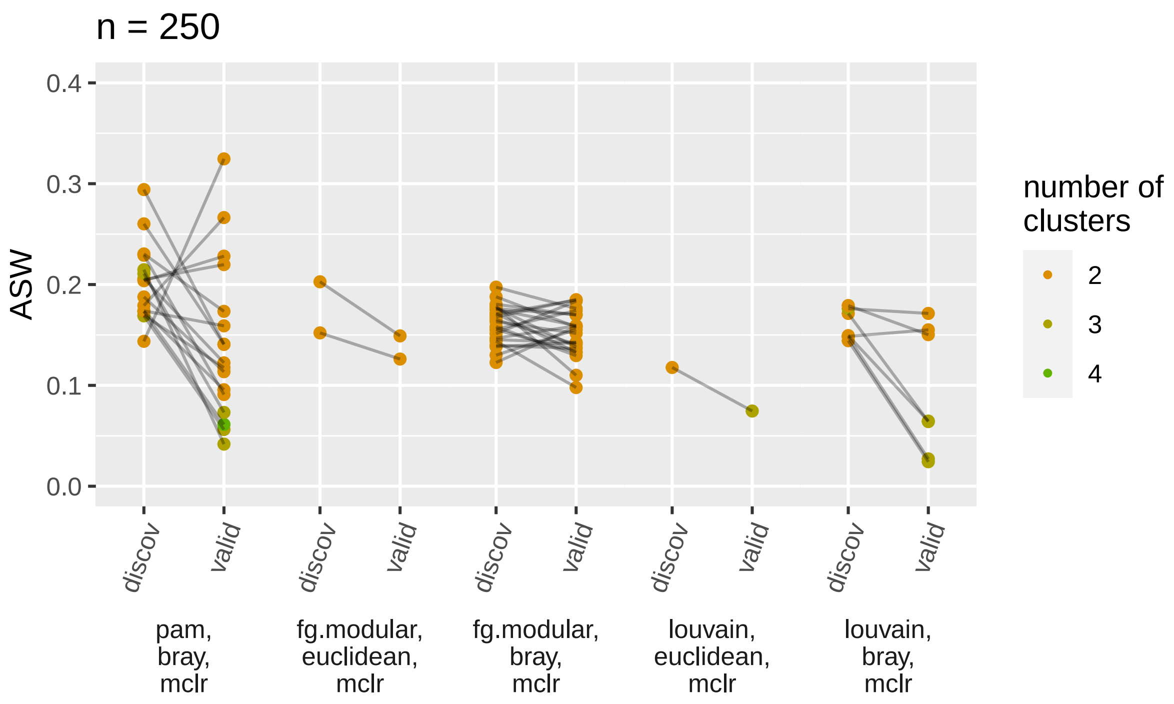

Fig L. Best ASWs for the clustering of samples on the discovery data, compared with the results on validation data, $n = 250$

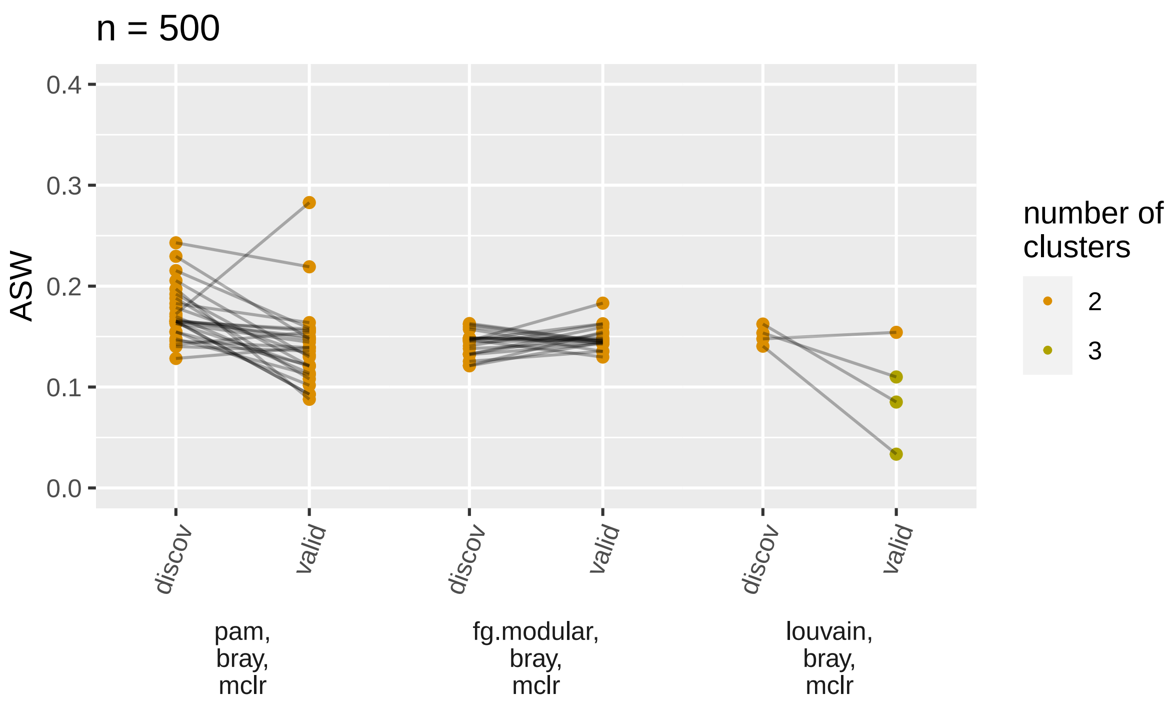

Fig M. Best ASWs for the clustering of samples on the discovery data, compared with the results on validation data, $n = 500$

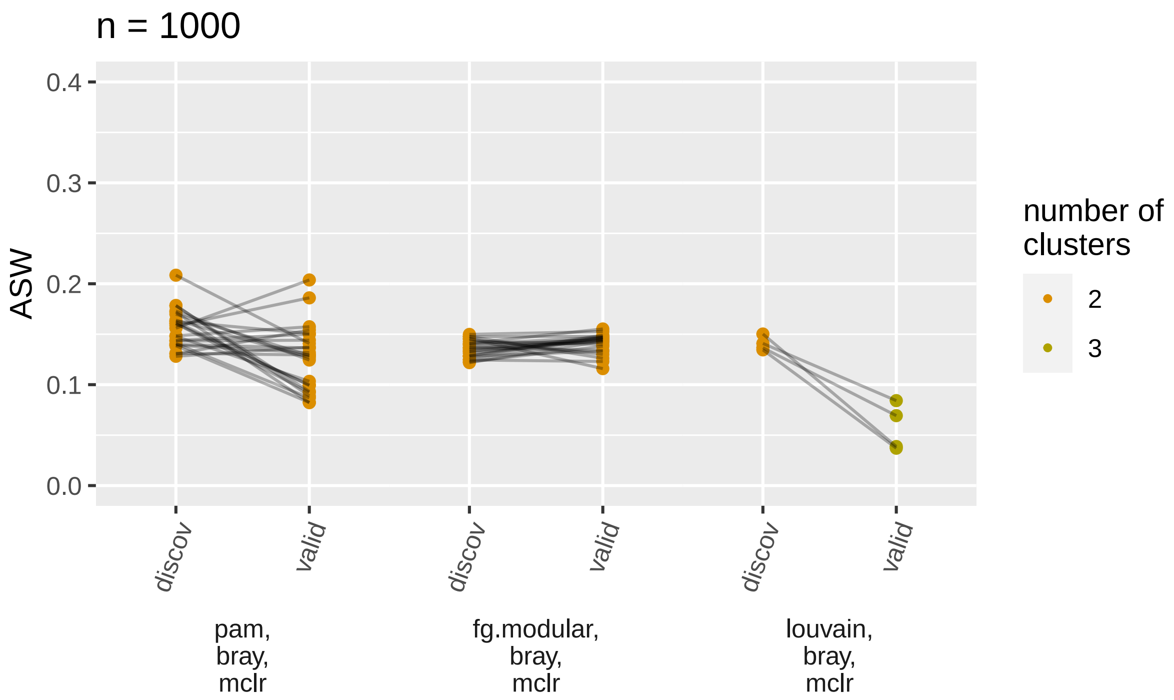

Fig N. Best ASWs for the clustering of samples on the discovery data, compared with the results on validation data, $n = 1000$

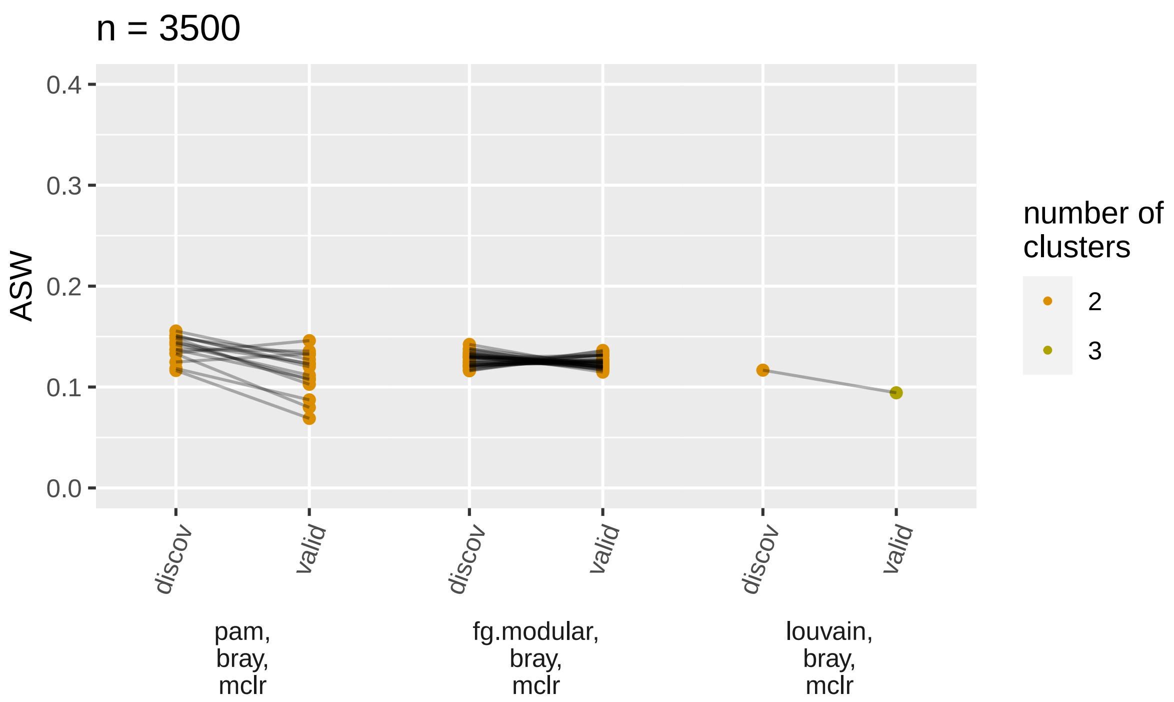

Fig O. Best ASWs for the clustering of samples on the discovery data, compared with the results on validation data, $n = 3500$

References

1. Arumugam M, Raes J, Pelletier E, Le Paslier D, Yamada T, Mende DR, et al. Enterotypes of the human gut microbiome. Nature. 2011;473:174–180.

2. Wu GD, Chen J, Hoffmann C, Bittinger K, Chen YY, Keilbaugh SA, et al. Linking long-term dietary patterns with gut microbial enterotypes. Science. 2011;334(6052):105–108.

3. Costea PI, Hildebrand F, Arumugam M, Bäckhed F, Blaser MJ, Bushman FD, et al. Enterotypes in the landscape of gut microbial community composition. Nature Microbiology. 2018;3:8–16.

4. Cheng M, Ning K. Stereotypes about enterotype: the old and new ideas. Genomics, Proteomics & Bioinformatics. 2019;17(1):4–12.
