# Supplementary material for: Over-optimism in unsupervised microbiome analysis: Insights from network learning and clustering
Source: PLoS Comput Biol. 2023 Jan 6;19(1):e1010820. doi: 10.1371/journal.pcbi.1010820 (PMC9873197; doi:10.1371/journal.pcbi.1010820)
Supplement: S5 Text — (PDF) [file pcbi.1010820.s005.pdf]

S5: Analyses with a reduced number of method combinations

We expected over-optimistic bias to decrease if fewer method combinations were tried. To investigate this hypothesis, we repeated our analyses with a reduced number of method combinations: 5 instead of 58 for the clustering of bacterial genera, 3 instead of 14 for hub detection and differential network analysis, and 5 instead of 31 for the clustering of samples.

The subsets of method combinations were chosen as follows:

Research task 1 (clustering of bacterial genera): The method of association estimation was fixed and only the type of cluster algorithm was varied (hierarchical clustering, spectral clustering [1], fast greedy modularity optimization [2], Louvain community detection [3], and manta [4]), leading to five method combinations overall. For (dis)similarity based clustering, association estimation was performed with the semi-parametric rank-based correlation (latentcor) [5, 6] combined with the mclr normalization. For network-based clustering, we used the SPRING method [7], which combines the latentcor correlation estimation with the neighborhood selection technique [8] for sparse estimation of partial correlations. The latentcor and SPRING methods were chosen because they are the most recently proposed methods and can be tentatively considered as "state of the art" among compositionally aware association estimation methods.

Research task 2 (hub detection): We chose three method combinations for network generation that represent three different classes of association estimation: Pearson correlation with clr normalization and sparsification via t-test (as an example of a simple method based on classical correlation estimation), the SPRING method (as a more advanced method that can estimate partial correlations), and the proportionality measure [9, 10] with clr normalization and sparsification via threshold (as an alternative approach that is not based on correlations).

Research task 3 (differential network analysis): The same three method combinations that were used in hub detection were selected.

Research task 4 (clustering of samples): Analogously to the first research task, the method for calculating dissimilarities between the samples was fixed and only the choice of cluster algorithm was varied, resulting in five method combinations. For DMM clustering [11], dissimilarities are not required. For the other cluster algorithms, dissimilarities were calculated with the Aitchison distance [12] which is a very well-known and popular method for this purpose. The dissimilarities were then used as input for PAM [13] and spectral clustering. Moreover, clustering with fast greedy modularity optimization and Louvain community detection was applied to the sparsified dissimilarities, where sparsification was performed with the K-nearest neighbor method.

The results are displayed in Tables A and B which have the same structure as Tables 1 and 2 in the main manuscript. They show the mean, median, and standard deviation of the difference as well as the scaled difference between the value of the evaluation criterion on the validation data and the value on the discovery data (over the 50 samplings of discovery/validation data). Additionally, the effect sizes (mean divided by standard deviation) are reported.

**Research task 1: clustering of bacterial genera**								
	$ARI_{valid} - ARI_{discov}$				$\frac{ARI_{valid}-ARI_{discov}}{ARI_{discov}}$			
n	mean	median	sd	mean/sd	mean	median	sd	mean/sd
100	-0.024	-0.021	0.045	-0.53	-13.0%	-15.7%	28.4%	-0.46
250	-0.029	-0.012	0.051	-0.57	-15.9%	-8.1%	29.7%	-0.53
500	-0.019	-0.013	0.039	-0.49	-9.9%	-8.6%	23.1%	-0.43
1000	-0.030	-0.026	0.035	-0.86	-17.1%	-16.3%	19.4%	-0.88
4000	-0.014	-0.007	0.029	-0.48	-8.2%	-4.3%	17.6%	-0.47
**Research task 2: hub detection**								
	$\#hubs_{valid} - \#hubs_{discov}$				$\frac{\#hubs_{valid}-\#hubs_{discov}}{\#hubs_{discov}}$			
n	mean	median	sd	mean/sd	mean	median	sd	mean/sd
100	-1.72	-1	2.47	-0.70	-18.2%	-14.3%	27.4%	-0.66
250	-0.70	-0.5	2.22	-0.32	-4.8%	-4.5%	25.9%	-0.19
500	-0.62	-1	1.94	-0.32	-4.8%	-9.5%	20.6%	-0.23
1000	-0.78	-1	1.97	-0.40	-7.3%	-11.1%	23.0%	-0.32
4000	-0.90	-1	1.61	-0.56	-9.4%	-11.1%	18.7%	-0.50

Table A. For research tasks 1 and 2: Mean, median, and standard deviation (over 50 samplings of discovery/validation data) of the difference (both unscaled and scaled) between the value of the evaluation criterion on the validation data and the corresponding value on the discovery data. Additionally, the effect size (mean divided by standard deviation) is reported. ARI_{discov} denotes the best ARI on the discovery data and ARI_{valid} the ARI resulting from the corresponding method combination on the validation data. The quantities $\#hubs_{discov}$, $\#hubs_{valid}$ (number of hubs) are defined analogously.

As Tables A and B show, the means and medians of the differences are negative for most research tasks and sample sizes. The only exception can be seen for the scaled GCD differences for the third research task; here, the means are all positive, indicating better results on the validation data on average. However, the corresponding standard deviations are large and the effect sizes are very small, indicating that the "improved" results on the validation data should probably not be over-interpreted. More detailed analyses show that the positive means are largely driven by a few outliers. Indeed, the *median* scaled differences are still negative, as are the mean and median unscaled differences.

Overall, the results indicate that some over-optimistic bias still exists even if fewer method combinations are tried. However, as expected, the absolute values of the mean/median

**Research task 3: differential network analysis**								
	$GCD_{valid} - GCD_{discov}$				$\frac{GCD_{valid}-GCD_{discov}}{GCD_{discov}}$			
n	mean	median	sd	mean/sd	mean	median	sd	mean/sd
100	-0.063	-0.130	0.649	-0.10	11.6%	-24.9%	101.2%	0.11
250	-0.213	-0.154	0.628	-0.34	3.3%	-21.2%	101.4%	0.03
500	-0.066	-0.025	0.289	-0.23	0.7%	-9.1%	71.6%	0.01

**Research task 4: clustering of samples**								
	$ASW_{valid} - ASW_{discov}$				$\frac{ASW_{valid}-ASW_{discov}}{ASW_{discov}}$			
n	mean	median	sd	mean/sd	mean	median	sd	mean/sd
100	-0.023	-0.017	0.068	-0.34	-9.8%	-12.4%	41.0%	-0.24
250	-0.011	-0.014	0.025	-0.45	-12.2%	-20.0%	37.8%	-0.32
500	-0.006	-0.005	0.017	-0.33	-6.3%	-9.6%	33.2%	-0.19
1000	-0.007	-0.005	0.013	-0.58	-12.3%	-10.0%	25.0%	-0.49
3500	-0.001	-0.002	0.010	-0.07	0.0%	-5.9%	25.4%	0.00

Table B. For research tasks 3 and 4: Mean, median, and standard deviation (over 50 samplings of discovery/validation data) of the difference (both unscaled and scaled) between the value of the evaluation criterion on the validation data and the corresponding value on the discovery data. Additionally, the effect size (mean divided by standard deviation) is reported. GCD_{discov} denotes the largest GCD on the discovery data and GCD_{valid} the GCD resulting from the corresponding method combination on the validation data. The quantities ASW_{discov}, ASW_{valid} (average silhouette width) are defined analogously.

differences as well as the effect sizes tend to be smaller compared to Tables 1 and 2. Put differently, over-optimistic bias is less pronounced if fewer method combinations are tried. Of course, the exact amount of over-optimistic bias depends on the chosen (subsets of) method combinations, i.e., the results might be slightly different when choosing different subsets of methods.

Tables C and D show additional stability analyses for the first and second research task based on the reduced number of tried method combinations, analogously to Tables 3 and 4 in the main manuscript. Overall, the index values are similar compared to Tables 3 and 4, i.e., the extent of stability remains roughly the same when reducing the number of tried methods. For the second research task (hub detection), the Jaccard values are somewhat smaller for the reduced number of tried methods at sample sizes of $n = 100$ and $n = 4000$. This might be explained by the following observation: at these sample sizes, the SPRING method is more frequently selected in the setting with the reduced number of methods combinations compared to the setting with the full set of method combinations; at the same time, SPRING tends to yield lower stability values. However, based on this limited analysis, we cannot determine whether SPRING generally tends to produce more unstable results with respect to hub detection.

	ARI_{stab}		
n	mean	median	sd
100	0.408	0.403	0.138
250	0.491	0.415	0.175
500	0.599	0.558	0.180
1000	0.620	0.587	0.177
4000	0.807	0.886	0.164

Table C. Mean, median, and standard deviation of ARI_{stab}, i.e., the ARI between the clusterings on discovery and validation data, over 50 samplings of discovery/validation data.

	Jaccard			Cosine similarity		
n	mean	median	sd	mean	median	sd
100	0.127	0.083	0.106	0.834	0.878	0.130
250	0.339	0.333	0.135	0.906	0.955	0.109
500	0.465	0.458	0.144	0.950	0.964	0.047
1000	0.539	0.545	0.134	0.945	0.967	0.062
4000	0.548	0.569	0.186	0.944	0.965	0.054

Table D. Mean, median, and standard deviation (over 50 samplings of discovery/validation data) of a) the Jaccard index which compares the set of hubs obtained on the discovery data with the set of hubs on the validation data, and b) the cosine similarity which compares these sets of hubs, but on the level of families.

References

1. Ng A, Jordan M, Weiss Y. On spectral clustering: Analysis and an algorithm. Advances in Neural Information Processing Systems. 2001;14:849–856.

2. Clauset A, Newman ME, Moore C. Finding community structure in very large networks. Physical Review E. 2004;70(6):066111.

3. Blondel VD, Guillaume JL, Lambiotte R, Lefebvre E. Fast unfolding of communities in large networks. Journal of Statistical Mechanics: Theory and Experiment. 2008;2008(10):P10008.

4. Röttjers L, Faust K. Manta: A clustering algorithm for weighted ecological networks. Msystems. 2020;5(1):e00903–19.

5. Yoon G, Carroll RJ, Gaynanova I. Sparse semiparametric canonical correlation analysis for data of mixed types. Biometrika. 2020;107(3):609–625.

6. Yoon G, Müller CL, Gaynanova I. Fast computation of latent correlations. Journal of Computational and Graphical Statistics. 2021;30(4):1249–1256.

7. Yoon G, Gaynanova I, Müller CL. Microbial networks in SPRING - Semi-parametric rank-based correlation and partial correlation estimation for quantitative microbiome data. Frontiers in Genetics. 2019;10:516.

8. Meinshausen N, Bühlmann P. High-dimensional graphs and variable selection with the lasso. Annals of Statistics. 2006;34(3):1436–1462.

9. Lovell D, Pawlowsky-Glahn V, Egozcue JJ, Marguerat S, Bähler J. Proportionality: a valid alternative to correlation for relative data. PLoS Computational Biology. 2015;11(3):e1004075.

10. Quinn TP, Richardson MF, Lovell D, Crowley TM. propr: an R-package for identifying proportionally abundant features using compositional data analysis. Scientific Reports. 2017;7(1):1–9.

11. Holmes I, Harris K, Quince C. Dirichlet multinomial mixtures: generative models for microbial metagenomics. PloS One. 2012;7(2):e30126.

12. Aitchison J. On criteria for measures of compositional difference. Mathematical Geology. 1992;24(4):365–379.

13. Kaufman L, Rousseeuw PJ. Finding Groups in Data. John Wiley & Sons, Ltd; 1990.
